# Harnessing the glycolysis-TCA cycle axis to boost host defense against neonatal infection

**DOI:** 10.1038/s44321-026-00463-z

**Published:** 2026-06-08

**Authors:** Ziyuan Wu, Nguyen Tran Nam Tien, Björn Klabunde, Karoline Aasmul-Olsen, Simone Margaard Offersen, Nguyen Thi Hai Yen, Tik Muk, Anna Hammerich Thysen, Susanne Brix, Nicklas Brustad, Tingting Wang, Jakob Stokholm, Klaus Bønnelykke, Anders Brunse, Nguyen Phuoc Long, Bo Chawes, Ole Bæk, Duc Ninh Nguyen

**Affiliations:** 1 Comparative Pediatrics, Department of Veterinary and Animal Sciences, University of Copenhagen, Frederiksberg, Denmark; 2 Department of Pharmacology and PharmacoGenomics Research Center, Inje University College of Medicine, Busan, Republic of Korea; 3 Department of Biotechnology and Biomedicine, Technical University of Denmark, Kongens Lyngby, Denmark; 4 COPSAC, Copenhagen Prospective Studies on Asthma in Childhood, Herlev and Gentofte Hospital, University of Copenhagen, Copenhagen, Denmark; 5 Graduate Institute of Biomedical Sciences, College of Medicine, Chang Gung University, Taoyuan, Taiwan; 6 Molecular Medicine Research Center, Chang Gung University, Taoyuan City, Taiwan; 7 Department of Clinical Medicine, Faculty of Health and Medical Sciences, University of Copenhagen, Copenhagen, Denmark; 8 BRIDGE Translational Excellence Programme, Faculty of Health Sciences, University of Copenhagen, Copenhagen, Denmark

**Keywords:** Metabolism, Microbiology, Virology & Host Pathogen Interaction

## Abstract

Preterm infants are highly susceptible to infections that can lead to sepsis, yet therapies beyond antibiotics are limited. Nutrition and host energy metabolism are known as immune modulators, but how they interact to mediate newborn host infection defense remains poorly understood. Here, we identify tricarboxylic acid (TCA) cycle metabolites as key modulators of early life infection outcomes. First, in a birth cohort of 700 children, elevated plasma TCA metabolite levels were associated with reduced infection burdens and systemic inflammation. Next, in a piglet neonatal sepsis model, sustained hepatic TCA cycle activity was associated with survival. These led us to explore clinically relevant nutritional strategies boosting TCA cycle activity. Substituting glucose in parenteral nutrition for galactose or glucogenic amino acids improved both pathogen clearance and preserved glucose homeostasis and prevented lethal sepsis. Mechanistically, these interventions promoted hepatic metabolic rewiring from glycolysis toward TCA-cycle-based oxidative phosphorylation, while mitigating excessive inflammation and organ injury. Our findings establish a clear connection between systemic energy metabolism and neonatal infection defense, suggesting clinically relevant strategies to improve outcomes in vulnerable newborns.

## Synopsis

The role of nutrition in regulating infection defense in preterm newborns remains poorly understood. The glycolysis-TCA cycle axis has been identified as a key modulator of infection outcomes, suggesting potential nutritional interventions to improve protection against neonatal sepsis.

- In a cohort of 700 infants, TCA cycle metabolites in early life were associated with decreased infection burden and systemic inflammation.
- The same metabolites were validated in infected preterm pigs, showing association of higher metabolite levels with infection survival.
- Using galactose instead of glucose in parenteral nutrition for infected preterm newborn piglets improved infection survival, mitochondrial metabolism and disease tolerance.
- Replacing part of glucose in parenteral nutrition with glucogenic amino acids during neonatal infection in preterm piglets completely prevented infection mortality, improved bacterial clearance, and enhanced mitochondrial metabolism and anti-inflammatory responses to protect against organ injury.
- In the infant metabolome, TCA intermediates correlated strongly with glucogenic amino acids, which in turn were associated with reduced infection burden and lower levels of inflammation.

## Abstract (web summary)

The role of nutrition in regulating infection defense in preterm newborns remains poorly understood. The glycolysis-TCA cycle axis has been identified as a key modulator of infection outcomes, suggesting potential nutritional interventions to improve protection against neonatal sepsis.

## Graphical abstract

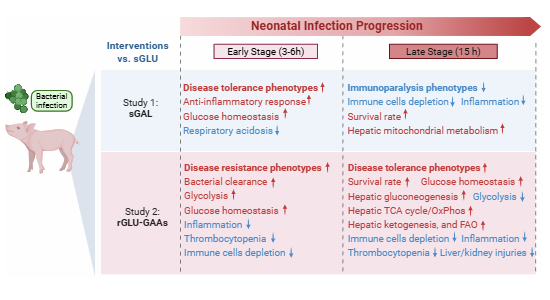

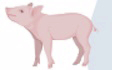

The paper explained **Problem**

Millions of preterm infants develop infection during the first month of life. If not controlled properly, infections can lead to life-threatening organ dysfunctions called sepsis, which has high mortality and long-term consequences later in life. Most of the very preterm infants have an immature gut and require glucose-rich parenteral nutrition (PN, nutrition directly into the bloodstream) for growth. But this practice leads to common hyperglycemia. Recent studies showed that high blood glucose levels drive systemic glycolysis and hyperinflammation, causing tissue damage.

**Results**

In this work, we combined studies in a human cohort of 700 infants, an in vitro model, and a neonatal sepsis model in preterm piglets to demonstrate the potential of new PN interventions that can better prevent neonatal sepsis. We identified TCA cycle activities as being critical modulators of inflammatory responses and infection outcomes. Higher levels of TCA metabolites were associated with decreased infection burdens and inflammation in both infants and preterm piglets. Enhancement of TCA cycle activities could be achieved by using PN containing galactose or glucogenic amino acids instead of glucose, which boosted host defense against infection and prevent neonatal sepsis.

**Impact**

Our findings show proof-of-concept novel PN interventions that can be instrumental to clinicians caring for infection-sensitive preterm infants. Our study can pave the way for phase-1 clinical trials testing the safety and later efficacy of using PN containing galactose and/or glucogenic amino acids instead of glucose-rich PN.

## Introduction

Newborn infants, especially preterm infants, are highly susceptible to infections that can progress to neonatal sepsis, a state of excessive inflammation causing organ damage with high risks of mortality (Stoll et al, 2015). High infection risk in newborns has conventionally been attributed to their immature immune system with a limited capacity to respond to exogenous stimuli (Collins et al, 2018). However, this hypo-responsiveness may also be influenced by the newborn’s energy metabolism (Harbeson et al, 2018). In adults with sufficient energy stores, immune cells undergo a metabolic shift from oxidative phosphorylation (OxPhos) to glycolysis upon infection to facilitate rapid ATP production, fueling inflammatory responses that eliminate invading pathogens at a cost to host fitness, a defense strategy termed *disease resistance* (Medzhitov et al, 2012). Newborns, in contrast, especially those born prematurely or with low birth weight, have limited energy reserves but high energy demands for growth. As a result, energy allocation in early life is thought to prioritize vital organ function over energetically costly inflammatory responses to infection, a defense strategy term *disease tolerance*. This strategy relies on anti-inflammatory immune activity and local tissue mechanisms that suppress inflammatory signals, processes largely supported by mitochondrial OxPhos (Medzhitov et al, 2012; Harbeson et al, 2018). However, when pathogen growth exceeds a certain threshold, the glycolysis-mediated *resistance* mechanisms occur in an excessive manner, causing hyperinflammation with collateral tissue damage and organ dysfunctions (Wu et al, 2024). In preterm infants, glucose-rich parenteral nutrition (PN) is often given during the first few weeks of life, but it increases the risk of hyperglycemia, which is linked to a higher infection rate and sepsis severity (Beardsall et al 2010; Alaedeen et al, 2006; Moltu et al, 2021). This practice may impair *disease tolerance* by skewing metabolism towards glycolysis instead of OxPhos during infections, contributing to poorer outcomes.

In a preterm pig model of a severe bloodstream infection, we have shown that *disease tolerance* is linked to improved survival and enhanced hepatic OxPhos, and that reducing parenteral glucose supply during infection further enhances survival by shifting *hepatic* metabolism from glycolysis toward OxPhos, resulting in more balanced systemic immune responses (Muk et al, 2022; Bæk et al, 2024; Wu et al, 2024). A key component of mitochondrial OxPhos is the tricarboxylic acid (TCA) cycle, which can be fueled by various intermediary metabolites, including amino acids, acetyl-CoA, and pyruvate. Further, TCA-cycle metabolites are not confined to mitochondria, and they can act as signaling molecules in immune cells to promote anti-inflammatory response (Mart**í**nez-Reyes and Chandel, 2020; Liu et al, 2020; Trauelsen et al, 2021; Dom**í**nguez-Andrés et al, 2019; Park et al, 2018). These suggest that the overall activity of the TCA cycle, in both liver and immune cells, may affect *disease tolerance* against serious infections in newborns. In addition, since energy metabolism in PN-nourished newborns can be modified by macronutrient intake (Bæk et al, 2024; Muk et al, 2022), novel PN compositions may help promote better defense strategies during serious bacterial infections, reducing inflammation-induced organ damage while allowing antibiotics to eliminate the pathogen.

In this work, we first examine the association between plasma levels of TCA cycle metabolites in a cohort of 700 infants (Bisgaard et al, 2013) and find that higher levels at birth are linked to reduced infection risk and lower inflammatory responses during early childhood. Then, using a human macrophage cell line and a preterm pig model of neonatal sepsis, we show that novel PN macronutrient compositions can enhance TCA cycle activity and improve infection outcomes by supporting *disease tolerance*. Specifically, substituting PN glucose with galactose improved glucose homeostasis and *disease tolerance*, while replacing it with glucogenic amino acids, enhanced both *disease resistance* and *tolerance*, ultimately leading to improved infection survival. These benefits were linked to hepatic metabolic rewiring and reduced organ injury. Based on these findings, we propose novel nutritional strategies based on galactose and glucogenic amino acid supplements to improve infection outcomes in early life. Further, by integrating data from in vitro studies, animal models, and human cohorts, we provide mechanistic insight and proof-of-concept on how enhancing systemic TCA cycle activity affects newborn host defense.

## Results

### Mother–child cohort characterization

**Figure 1:**
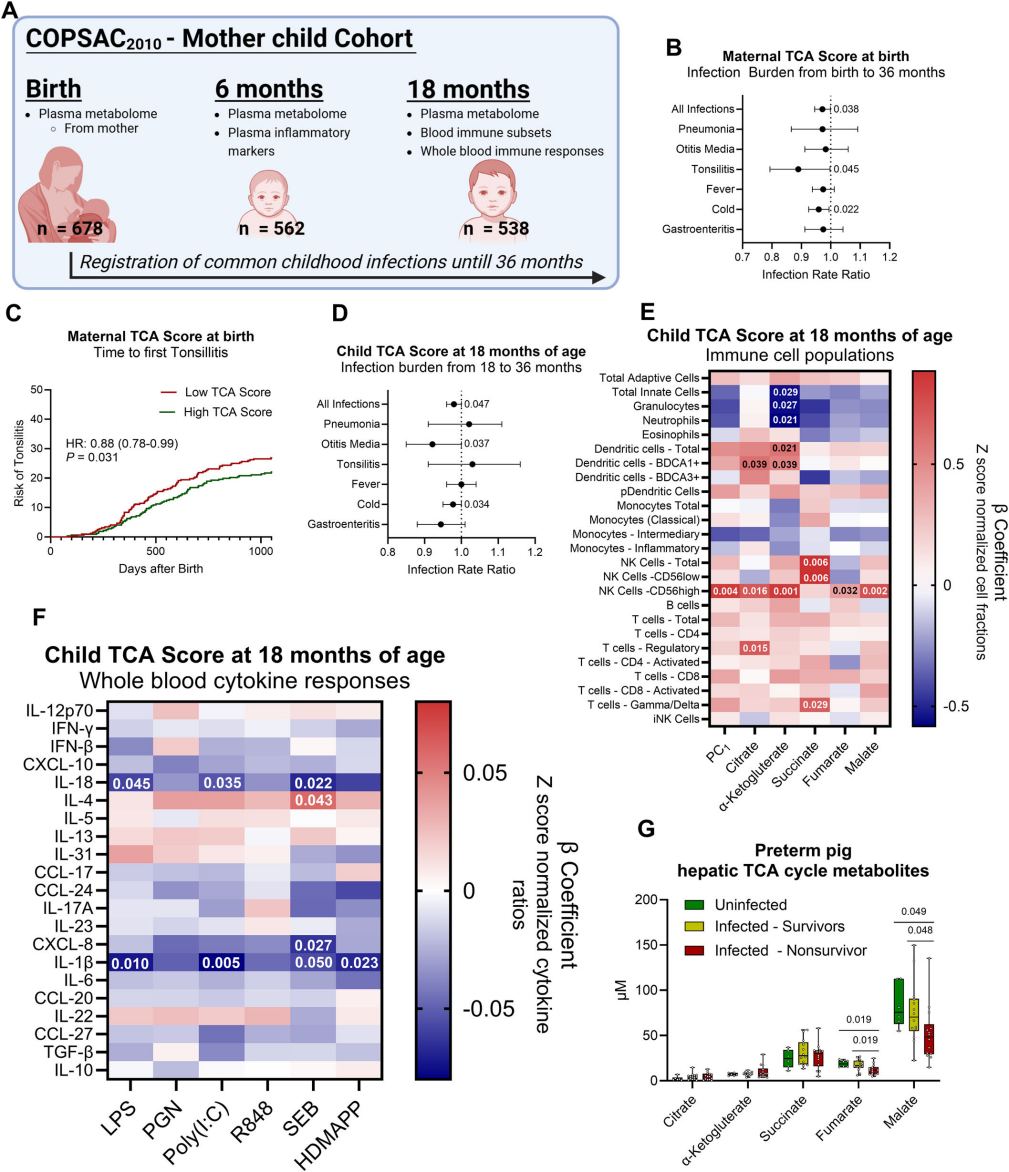
Plasma TCA cycle metabolites associate with infection burden and immune function in early childhood. (**A**) Overview of infant cohort and sampling. TCA metabolite levels (TCA Score) were linked to infection burden (0–36 months), inflammatory markers (6 months), and immune phenotypes (18 months), reported in (**B**–**F**). (**B**) Association between maternal TCA scores at 1 week postpartum (*n* = 678) and total infection counts, analyzed by quasi-Poisson regression, shown as estimated risk ratios with 95% CIs. (**C**) Kaplan–Meier plot of time to first tonsillitis episode stratified by maternal TCA scores (high vs. low, *n* = 678); Cox model results shown. (**D**) Association between 18-month TCA scores and infection counts at 36 months analyzed by quasi-Poisson regression shown as estimated risk ratios with 95% CIs (*n* = 538). (**E**) Heatmap of associations between 18-month TCA scores and immune cell fractions by flow cytometry (*n* = 538). Shown as β-coefficients between z-score normalized immune cell proportion and TCA score or relative abundance of TCA cycle metabolites following a generalized linear model, where red color indicates positive, and blue indicates negative association. (**F**) Heatmap of associations between 18-month TCA scores and cytokine responses to PRR agonists in whole blood, shown as β-coefficients between z-score normalized cytokine response and TCA score following a generalized linear model, where red color indicates positive, and blue negative association (*n* = 538). LPS lipopolysaccharide, PGN peptidoglycan, R848 imidazoquinoline, SEB staphylococcal enterotoxin B, HDMAPP 1-hydroxy-2-methyl-2-buten-4-yl 4-diphosphate. (**G**) Absolute concentrations of key TCA-cycle metabolites in hepatic tissue from newborn preterm pigs infused with control saline (*n* = 6) or infected with *Staphylococcus epidermidis*, categorized as survivors (*n* = 14) or non-survivors (*n* = 14), analyzed by generalized linear model, shown as box-and-whiskers with corresponding median and total range. Exact *P* values were indicated in each figure panel. Source data are available online for this figure.

To explore the relation between TCA cycle metabolites, infection risk, and immune function in human newborns, we analyzed data from the Copenhagen Prospective Studies on Asthma in Childhood 2010 (COPSAC_2010_) birth cohort. This cohort included 700 mother–child pairs, closely monitored from birth through the first three years of life with daily diary registration of infections (Brustad et al, 2024). Detailed characteristics of the cohort have been published previously (Chawes et al, 2016). The comprehensive documentation of common childhood infections was combined with plasma LC-MS metabolome and immune profiling to provide a detailed dataset for analysis (see Fig. 1A). At 1 week postpartum (*n* = 678), and from the children at 6 (*n* = 562) and 18 months (*n* = 538), semi-quantitative levels of citrate, α-ketoglutarate, succinate, fumarate, and malate could be assessed within quality control limits.

### TCA cycle metabolites, infection burden, and inflammatory responses in human newborns

**Figure EV1:**
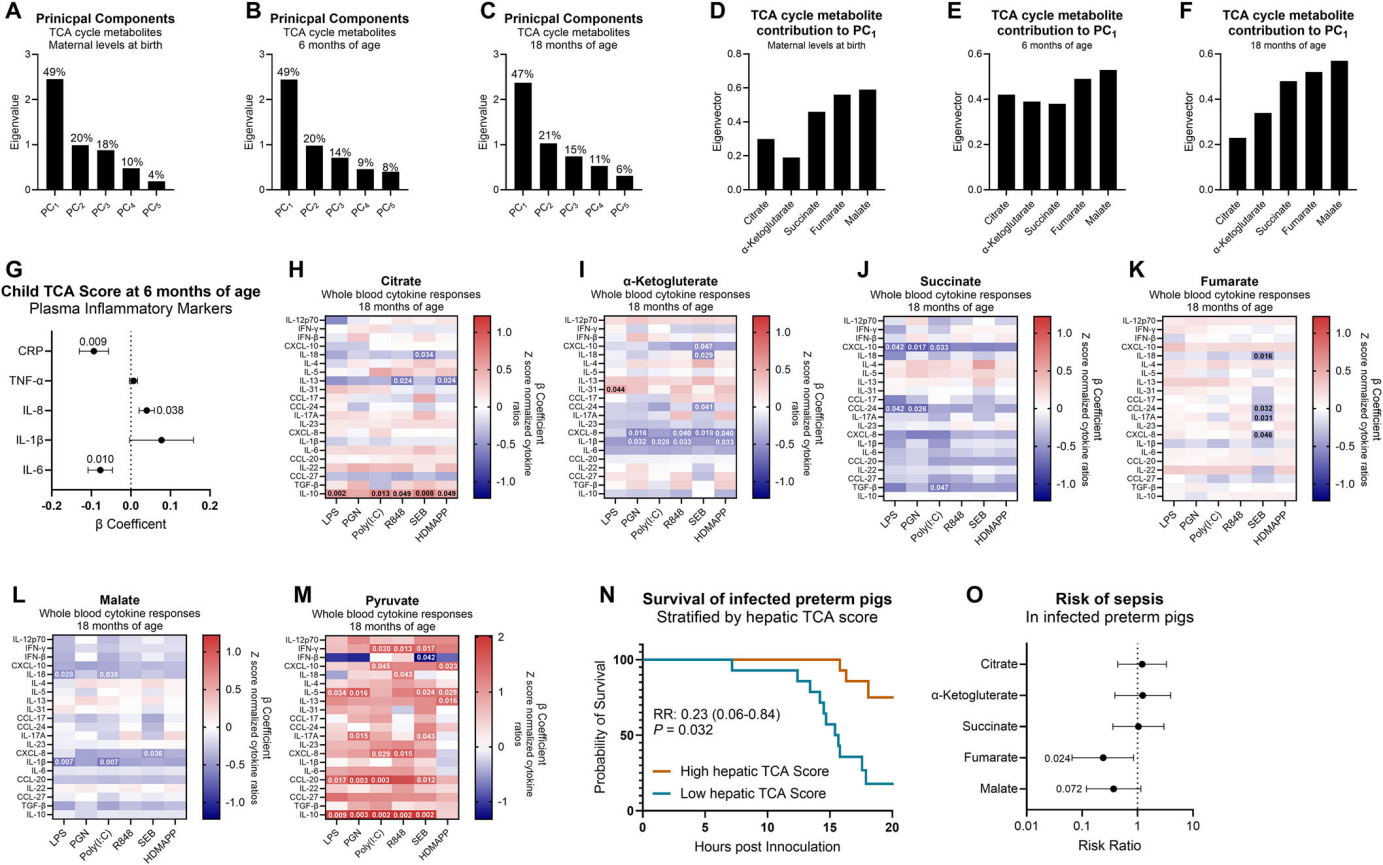
Association of TCA metabolites with inflammation in newborn infants and infected preterm piglets. (**A**–**C**) Eigenvalues showing the fraction each principal component (PC) contributes to the overall PCA at birth, 6, and 18 months of age. (**D**–**F**) Eigenvectors showing how each TCA metabolite contributes to PC_1_ (TCA scores) at birth, 6, and 18 months of age. (**G**) Associations between 6-month TCA scores and plasma inflammatory markers with estimated coefficient and 95% CIs. CRP, C-reactive protein; TNF, tumor necrosis factor; IL, Interleukin, analyzed by generalized linear model and shown as β-coefficients with 95% CIs. (**H**–**M**) Heatmaps showing the association between individual TCA cycle metabolites and pyruvate at 18 months of age and z-score normalized cytokine ratios following whole blood stimulation with 6 pattern recognition receptor agonists. Shown as β-coefficients following a generalized linear model, where red color indicates positive, and blue negative association. LPS lipopolysaccharide, PGN Peptidoglycan, R848 imidazoquinoline, SEB staphylococcal enterotoxin B, HDMAPP 1-Hydroxy-2-methyl-2-buten-4-yl 4-diphosphate. Data from (**A**–**M**) were from the COPSAC infant cohort. (**N**) Survival curves for preterm pigs infected with *S. epidermidis*, stratified by hepatic TCA score (high vs. low, divided at the median). Log-rank risk ratios (RR) with corresponding 95% confidence intervals are shown. (**O**) Estimated risk ratios with 95% CIs for risk of sepsis/mortality in infected preterm pigs based on the absolute hepatic levels of each individual TCA-cycle metabolite. Exact *P* values were indicated in each figure panel. For data in (**A**–**M**), *n* = 678 for maternal data, *n* = 562 and 538 for data at 6 and 18 months, respectively, *n* = 28 in (**N**, **O**).

The five key annotated TCA cycle metabolites (α-ketoglutarate, malate, succinate, citrate, and fumarate) were pre-selected for an unsupervised principal component analysis (PCA) to obtain components representing overall TCA cycle activity. In both the maternal metabolome at birth and the child metabolome at 6 and 18 months of age, individual TCA cycle metabolites contributed nearly equally and positively to Principal Component 1 (PC₁, from now assigned as TCA score), explaining 47–49% of the variation at each timepoint (Fig. EV1A–F).

When correcting for relevant confounders, higher maternal TCA cycle activity at birth, as reflected by higher TCA score, was associated with a reduced overall infection count, driven by fewer episodes of tonsillitis and cold symptoms in children during the first 3 years of life, as estimated by quasi-poisson regression (Fig. 1B). Higher TCA scores were also associated with increased time to first tonsillitis episode, as estimated by a Cox proportional hazard model (Fig. 1C), but not for other infection types. These effects appeared to be driven primarily by higher levels of citrate, fumarate, and malate (Table EV1). We have previously shown a high correlation between the maternal and newborn plasma metabolome, making the maternal metabolome at birth suitable for our analysis (Olarini et al, 2022). Strikingly, TCA scores at 18 months, derived from child plasma, were still associated with a lower risk of total infection episodes, now driven by fewer episodes of acute otitis media and cold symptoms, in the period between 18-36 months of age (Fig. 1D).

At 6 months of age, higher child TCA scores were associated with lower plasma levels of IL-6 and C-reactive protein, but higher levels of IL-8 (Fig. EV1G). At 18 months of age, higher child TCA scores were associated with higher frequencies of CD56^Bright^ Natural Killer (NK) cells (Fig. 1E). Individually, α-ketoglutarate by itself correlated with lower frequencies of neutrophils and higher frequencies of BDCA-1^+^ dendritic cells (DCs, Fig. 1E). Furthermore, citrate and succinate correlated with higher frequencies of regulatory T cells (T_Reg_) and γδ-T cells, respectively (Fig. 1E). These findings align with known metabolic dependencies of immune cell subsets, as neutrophils predominantly rely on glycolysis, while CD56^Bright^ NK cells, DCs, T_Reg_, and γδ T cells depend on OxPhos (Shyer et al, 2020; Pearce and Everts, 2015; Borregaard, 2010).

The changes in immune cell subsets at 18 months of age were reflected in cytokine responses to various PRR agonists and T cell ligands. Higher TCA scores were associated with reduced responses of the pro-inflammatory cytokines IL-1β and IL-18 after stimulation of whole blood with LPS, Poly(I:C), and SEB, and increased IL-4 responses to SEB (Fig. 1F). Among the five TCA cycle metabolites, levels of citrate were consistently associated with increased levels of the anti-inflammatory cytokine IL-10 across five of the six different PRR agonist stimulations (Fig. EV1H). Likewise, α-ketoglutarate, succinate, fumarate, and malate were associated with reduced responses of several pro-inflammatory cytokines (CXCL-10, IL-18, CXCL-8, and IL-1β) to several ligands (Fig. EV1I–L). Of glycolysis intermediary molecules, only pyruvate was detected and correlated with reduced infection risk from 18 to 36 months (Table EV2), but contrary to TCA cycle metabolites, pyruvate correlated with increased immune responses (mainly IL-10 and CCL-20) against most immune agonists (Fig. EV1M).

Together, these results suggest that higher plasma levels of TCA cycle metabolites at birth and during infancy correlate with reductions in infection burden and systemic inflammatory response. Although plasma metabolites reflect the whole host body metabolism, not specifically immune cell metabolism, these findings underscore the importance of energy metabolic pathways in shaping newborn immune responses. Likewise, there may be differences in each of the TCA metabolites, as e.g., citrate is consistently associated with increases in anti-inflammatory cell types and cytokine responses, whereas α-ketoglutarate is associated with reductions in innate cell populations and pro-inflammatory cytokine responses. These associations may also be relevant during serious infections in young children. Building on these insights, we next explored the causal impacts of modulated TCA cycle activity by novel nutritional interventions on host defense during neonatal infections.

### Hepatic TCA cycle activity is affected by bloodstream infection in preterm piglets

With a preterm piglet model of neonatal sepsis induced by bloodstream infection with *Staphylococcus epidermidis*(Bæk et al, 2020), we have shown that a standard parenteral glucose supply, similar to that given to preterm infants, results in hyperglycemia, excessive glycolysis-induced inflammation and sepsis, while glucose restriction lowers inflammation, increases hepatic OxPhos, but induces hypoglycemia (Bæk et al, 2024; Muk et al, 2022). To assess how infection affects hepatic TCA cycle activity under these conditions, newborn preterm pigs received PN containing standard glucose levels (14.4 g/kg/day, similar to what preterm infants receive (Mesotten et al, 2018a) and were infused with either *S. epidermidis*, the most common cause of neonatal sepsis in preterm newborns (*n* = 28) (Strunk et al, 2024), or saline control (*n* = 6). Animals were monitored for 20 h and euthanized when reaching sepsis criteria, humane endpoints predefined as arterial blood pH of ≤7.1, together with deep lethargy, apnea, or hypoperfusion. Among infected pigs, 50% (14/28) required early euthanasia due to sepsis. Targeted metabolomics of five key TCA cycle intermediates was performed on liver tissue collected at euthanasia. Infected non-survivors had significantly lower hepatic fumarate and malate levels than both infected survivors and uninfected controls (Fig. 1G). Likewise, a composite hepatic TCA score, constructed similarly to the COPSAC analysis, showed that infected piglets with higher TCA scores were markedly more likely to survive the infection (Fig. EV1N). Again, improved survival was associated with higher fumarate and malate levels (Fig. EV1O). These findings indicate that severe neonatal infection is associated with suppressed hepatic TCA cycle activity. Thus, interventions aiming to preserve hepatic TCA flux may improve infection survival.

### Replacing glucose with galactose improves *disease tolerance* and infection survival

Our first PN strategy investigated whether replacing glucose with an equally energy-dense carbohydrate could shift hepatic metabolism away from glycolysis and toward enhanced TCA cycle activity during infection. Galactose is a monosaccharide bound to glucose in the natural structure of lactose, the main carbohydrate of milk, and is cleaved by lactase during digestion prior to absorption. Despite its obvious safety, galactose has not been used as a direct nutritional supplement for infants. Galactose metabolism starts with the Leloir pathway prior to entering glycolysis (Frey, 1996), and we hypothesized that the slow conversion of galactose into glucose-6-phosphate can reduce systemic glycolysis during neonatal infection, increasing reliance on mitochondrial OxPhos, in turn promoting host *disease tolerance*.

**Figure 2:**
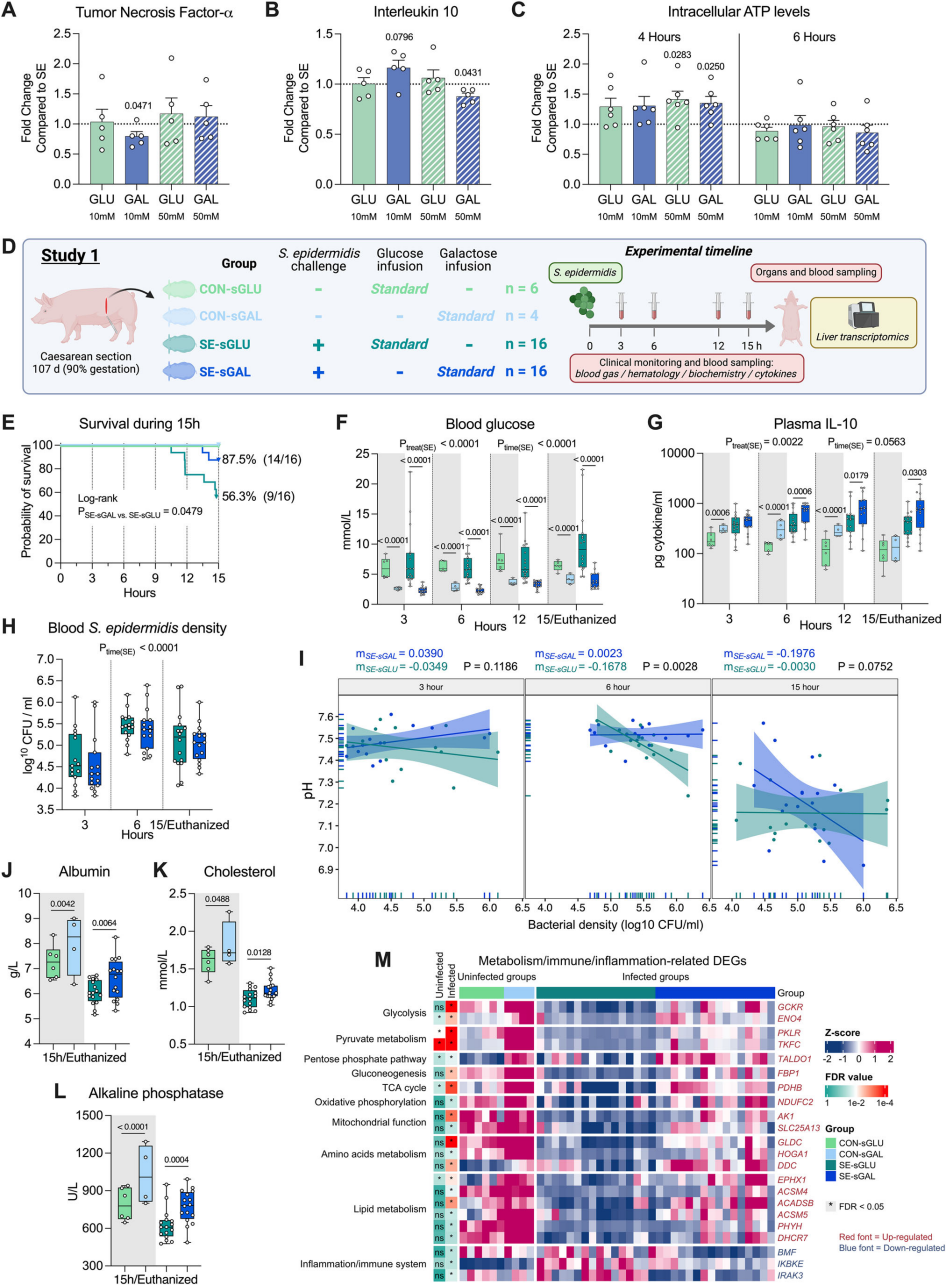
Galactose sustains normoglycemia, promotes disease tolerance, and lowers sepsis risk. (**A**–**C**) In vitro responses of human THP1 macrophage-like cells exposed to *S. epidermidis*, showing fold changes in TNF-α, IL-10, and intracellular ATP in replicates treated with Glucose and Galactose, relative to *S. epidermidis*-exposed untreated controls. Normality was assessed before statistical testing. For normally distributed data, paired *t* tests were used; otherwise, Wilcoxon matched-pairs signed-rank tests were used. Data are shown as bar plots with individual data points. Bars indicate the mean, and error bars indicate standard deviation. *P* values indicate comparisons with the corresponding infected control without added glucose or galactose. *N* = 5/group in (**A**, **B**) and *n* = 6 in (**C**). (**D**) Piglet experimental design, used to generate data in (**E**–**M**). (**E**) Survival curves up to 15 h post-inoculation or humane endpoint, compared via log-rank test. (**F**, **G**) Blood glucose and plasma IL-10 levels at 3, 6, 12 h, and euthanasia. (**H**) Blood *S. epidermidis* counts at 3, 6 h, or euthanasia. (**I**) Reaction norm analysis: blood pH vs. pathogen burden over time. Slopes compared via an extra sum-of-squares F test. (**J**–**L**) Serum albumin, cholesterol, and alkaline phosphatase at euthanasia. (**F**–**H**, **J**–**L**) Data at each time point were analyzed using a linear mixed-effects model, incorporating group, gender, and birth weight as fixed factors and litter as a random factor. (**F**–**H**) Another linear mixed-effects model was employed to probe further disparities spanning the entire experimental duration, incorporating group, time, their interaction, gender, and birth weight as fixed factors, with litter and pig ID as random factors. P_treat(SE)_, P_time(SE)_, and P_int(SE)_ denote probability values for group effect (SE-sGAL and SE-sGLU) over time, time effects, and the interaction effects between time and group in the linear mixed-effects interaction model, respectively. Uninfected animals (CON) served as the reference group and were not directly compared with infected animals (SE). Data are shown as box-and-whisker plots. The center line indicates the median; the lower and upper bounds of the box indicate the 25th and 75th percentiles, respectively; and the whiskers extend from the minimum to the maximum values. All individual data points are shown. (**M**) Heatmap of top differentially expressed genes between SE-sGAL and SE-sGLU groups. In (**E**–**M**), *n* = 15-16/group for infected groups, *n* = 4–6/group for uninfected groups. Source data are available online for this figure.

First, we tested how galactose and glucose mediate the response of human macrophage-like THP1 cells to live *S. epidermidis*. After 6 h of stimulation, low doses of galactose addition (10 mM) reduced production of TNF-α and tended to increase that of IL-10 (Fig. 2A,B). There was however, no difference in the capability of cells to produce ATP between galactose and glucose, or between doses (Fig. 2C), and minimal effects on gene expression (Appendix Fig. S1A). This suggests that providing galactose, rather than glucose, at physiologically relevant levels could reduce the pro-inflammatory activity of immune cells without compromising their energy metabolism.

Next, we tested the substitution of galactose for glucose in PN, using the same preterm pig neonatal sepsis model. Infected preterm piglets were reared with standard dose (14.4 g/kg/day) of PN glucose (sGLU) or galactose (sGAL), and intensively monitored until 15 h post-inoculation (Fig. 2D). Compared to SE-sGLU animals, SE-sGAL animals showed lower mortality due to sepsis (Fig. 2E). This was accompanied by a tendency for higher blood pH and lower carbon dioxide pressure (pCO_2_) over time (Appendix Fig. S1B,C), suggesting that galactose supply prevented respiratory acidosis. During the first 6 h, galactose-supplemented animals also showed a slight elevation of lactate (Appendix Fig. S1D), potentially indicating a stronger glycolysis-related immune response to resist the infection. Importantly, galactose-supplied animals were normoglycemic, with gradually increasing blood glucose levels from 2.4 to around 4 mM over time, indicating the ability to convert exogenous galactose into glucose (Fig. 2F).

In SE-sGAL animals, plasma IL-10 was elevated (Fig. 2G), while bacterial clearance (Fig. 2H) and levels of TNF-α and IL-6 (Appendix Fig. S1E,F) were unchanged. However, SE-sGAL animals had less depletion of blood neutrophils, monocytes, and hemoglobin (Appendix Fig. S1G–I). Thus, it appears that the clinical benefit of galactose supply was derived from improved *disease tolerance* instead of *resistance*, prioritizing the maintenance of normal organ function over a strong immune response to clear the pathogens. To investigate defense strategies further, we employed a reaction norm analysis by plotting changes in host health against pathogen burdens, as described previously (Wu et al, 2024). Blood pH was used as a readout for general health, and reaction norms were generated at 3, 6, and 15 h post-inoculation (Fig. 2I). Both infected groups displayed similar slopes at 3 h, but at 6 h SE-sGAL animals showed a shallower slope, whereas the SE-sGLU dropped in blood pH with increasing levels of blood bacteria. This indicates improved *disease tolerance* following galactose supply. At the end of the study, the difference between slopes was less pronounced, but there was now a tendency for decreased health in the SE-sGAL group when bacterial levels increased. In contrast, animals in SE-sGLU reached a paralysis state with consistently low health across various levels of blood bacteria.

At euthanasia, all infected animals showed lower levels of negative acute phase reactants (albumin, cholesterol, and alkaline phosphatase), but galactose supplementation alleviated drops in these markers (Fig. 2J–L). Other liver injury markers, including aspartate aminotransferase and alanine aminotransferase, as well as kidney injury marker creatinine, were not affected (Appendix Fig. S1J–M). In uninfected animals, galactose also affected TNF-alpha, Hb, and biochemical profiles, but the changes were minor compared with the effects of infection.

### Galactose supply reduces inflammation and enhances hepatic mitochondrial metabolism

Galactose can be converted to glucose in the liver and subsequently stored as glycogen or released directly into the bloodstream (Gannon et al, 2001). Therefore, we analyzed the immunometabolic impacts of galactose on the hepatic transcriptome. PCA showed distinct clusters of infected and uninfected animals (Appendix Fig. S2A). In an enrichment (GSEA) and differentially expressed gene (DEG) analysis, SE-sGAL animals exhibited upregulation of various metabolic pathways, including OxPhos, TCA cycle, glycolysis/gluconeogenesis, as well as downregulation of immune pathways, including Th1/Th2, Th17, and IL-17 signaling (Fig. 2M; Appendix Fig. S2B and Dataset EV1A). Similar metabolic effects of galactose were present in non-infected animals as well (Appendix Fig. S2C and Dataset EV1A). Taken together, the hepatic transcriptomic data further support our conclusions that galactose supply improved mitochondrial energy metabolism, preventing excessive inflammation and supporting *disease tolerance*.

### Glucose restriction plus GAAs prevents sepsis and organ dysfunction

**Figure 3:**
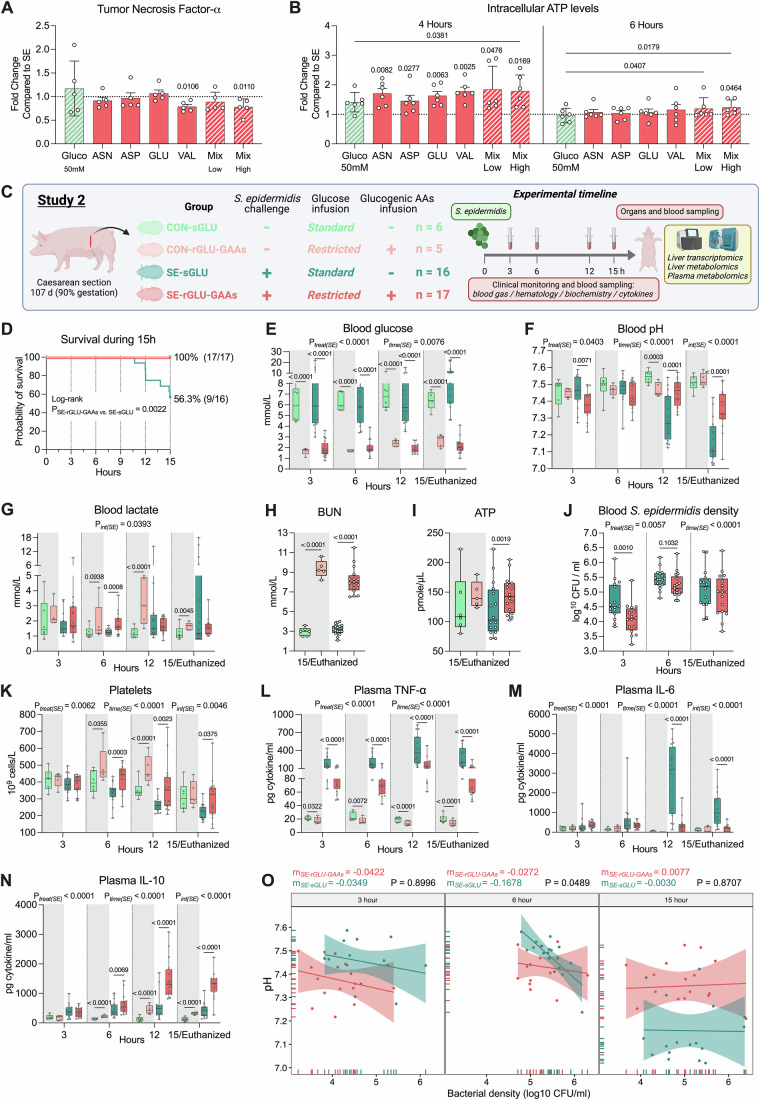
Combined glucose restriction and glucogenic amino acid supply prevent lethal sepsis and organ dysfunction. (**A**, **B**) In vitro responses of human THP1 macrophage-like cells exposed to *S. epidermidis*, showing fold changes in TNF-α and intracellular ATP in replicates treated with Glucose, Asparagine (ASN), Aspartate (ASP), Glutamate (GLU), and Valine (VAL), relative to *S. epidermidis*-exposed untreated controls. Normality was assessed before statistical testing. For normally distributed data, paired *t* tests were used; otherwise, Wilcoxon matched-pairs signed-rank tests were used. Data are shown as bar plots with individual data points. Bars indicate the mean, and error bars indicate SD. *P* values indicate comparisons with the corresponding infected control without added amino acids. *N* = 5–6/group. (**C**) Piglet experimental design, used to generate data in (**D**–**O**). (**D**) Survival curves up to 15 h post-inoculation or humane endpoint, compared via log-rank test. (**E**–**G**) Blood glucose, pH, and lactate at 3, 6, 12, and 15 h post-inoculation or at euthanasia. (**H**, **I**) Serum blood urea nitrogen (BUN) and plasma ATP at 15 h post-inoculation or euthanasia. Analyzed using the same linear mixed-effects model. (**J**) Blood *S. epidermidis* density at 3, 6 h, or euthanasia, determined by CFU assay. (**K**–**N**) Blood platelets and plasma TNF-α, IL-6, and IL-10 at 3, 6, 12, and 15 h or euthanasia. (**E**–**N**) Data at each time point were analyzed using a linear mixed-effects model, incorporating group, gender, and birth weight as fixed factors and litter as a random factor. (**E**–**G, J**–**N**) Another linear mixed-effects model was employed to probe further disparities spanning the entire experimental duration, incorporating group, time, their interaction, gender, and birth weight as fixed factors, with litter and pig ID as random factors. P_treat(SE)_, P_time(SE)_, and P_int(SE)_ denote probability values for group effect (SE-rGLU-GAAs and SE-sGLU) over time, time effects, and the interaction effects between time and group in the linear mixed-effects interaction model, respectively. Uninfected animals (CON) served as the reference group and were not directly compared with infected animals (SE). Data are shown as box-and-whisker plots. The center line indicates the median; the lower and upper bounds of the box indicate the 25th and 75th percentiles, respectively; and the whiskers extend from the minimum to the maximum values. All individual data points are shown. (**O**) Reaction norm analysis: blood pH vs. pathogen burden at 3, 6, and 15 h, slopes compared via extra sum-of-squares F test. In (**D**–**O**), *n* = 15–17/group for infected groups, and *n* = 5–6/group for uninfected groups. Source data are available online for this figure.

Replacing glucose with galactose enabled piglets to maintain stable blood sugar levels. However, it appeared to delay, not prevent clinical deterioration, possibly as galactose still enters glycolysis to fuel ATP production and inflammation. We therefore explored an alternative nutritional strategy to boost TCA cycle activity (to reduce inflammation) as well as gluconeogenesis (to ensure normoglycemia) during periods of glucose restriction. Glucogenic amino acids (GAA) can enter the TCA cycle, either directly or through intermediary steps, and form the backbone necessary for gluconeogenesis. First, we screened the immune responses of *S. epidermidis*-infected THP-1 cells following supplementation of either glucose or individual or a mixture of four strictly glucogenic amino acids (GAAs): glutamate, valine, aspartate, and asparagine. These GAAs were selected based on their easy conversion into TCA cycle metabolites, without directing to pyruvate and lactate production. Valine and high dose of the mixture (8 mM) reduced TNF-α production (Fig. 3A), with no impact on IL-10 and minimal effects on gene expression (Appendix Fig. S3A,B). However, all individual GAAs, as well as low (2 mM) and high doses of the mixture, increased ATP production after 4 h, compared to the infected controls (Fig. 3B). The high-dose mixture increased ATP production more effectively than glucose alone, also at 6 h. This suggests that GAA supplementation may enhance TCA and OxPhos in immune cells during infections while maintaining low levels of inflammation.

Based on this, another infection study was conducted with preterm piglets nourished either with standard glucose (sGLU) or restricted glucose with GAA supplementation (each amino acid 0.5 g/kg/day, rGLU–GAA, Fig. 3C). Strikingly, 100% of rGLU–GAA-treated animals were alive at 15 h post inoculation as opposed to 44% of infected sGLU succumbing to fatal sepsis (Fig. 3D). Further, treated animals maintained stable blood glucose levels of around 2 mM, in contrast to most infected controls being hyperglycemic (Fig. 3E). Clinical parameters also indicated gradual deterioration with respiratory acidosis in infected controls from 12 h until the end of the study, indicated by severe drops of pH (Fig. 3F), spO_2_, base excess, and hemoglobin, and elevation of pCO_2_ (Appendix Fig. S3C–F). In contrast, the intervention prevented all these disturbances. Similar to galactose-nourished animals, rGLU-GAAs animals exhibited a slight, yet significant and transient, rise in lactate levels at the early phase of infection (6 h, Fig. 3G). This increase suggests an enhanced conversion of the administered GAAs into pyruvate, which is then metabolized into lactate, likely through the TCA cycle. Moreover, the treated group at the end of the study exhibited increased blood urea nitrogen (BUN) and ATP production, suggesting the GAAs were metabolized through the TCA cycle and supported OxPhos (Fig. 3H,I). Biochemical parameters measured at euthanasia also indicate that glucose restriction in combination with GAAs protected against injuries to the liver (lower AST and ALT) and kidneys (lower creatinine), as well as prevented impairment in hepatic synthesis of negative acute-phase reactants (higher albumin and cholesterol) (Appendix Fig. S3G–K). Albumin and cholesterol levels were also higher in the CON-rGLU–GAA group, likely reflecting altered metabolism.

### Glucose restriction with GAA supplementation enhances both *disease resistance* and *tolerance*

With clear clinical benefits of the rGLU–GAA intervention during infection, we further examined parameters related to host defense strategies. Blood bacterial loads in the intervention group were reduced at both 3 and 6 h, indicating an improved *disease resistance*, eliminating pathogens during the early phase of infection (Fig. 3J). This was associated with better preservation of blood leukocyte subsets (Appendix Fig. S3L–O) and prevention of thrombocytopenia by the intervention (Fig. 3K). Notably, different from galactose intervention, rGLU-GAAs reduced levels of TNF-α and IL-6 and elevated levels of anti-inflammatory cytokine IL-10 (Fig. 3L–N) across all study timepoints. The differences in *disease resistance* and inflammatory status likely contribute to the substantial protective effects of rGLU–GAA treatment against organ injuries, which were not observed during the galactose intervention.

To evaluate *disease tolerance*, we again employed reaction norm analysis. At 3 h, both infected groups showed similar slopes (Fig. 3O, left). However, by 6 h, rGLU–GAA animals had improved *disease tolerance* as shown with a shallow slope, in contrast to the infected controls with a steep decrease in health when bacterial burdens increased (Fig. 3O, middle). At euthanasia, although the slopes of the reaction norms were similar between the two groups (Fig. 3O, right), the low blood pH suggests that infected controls were in a state of immunoparalysis, whereas SE-rGLU-GAAs animals still maintained vigor. Collectively, the combined glucose restriction and GAA supplementation boosted *disease resistance* in the early phase of infection and improved *disease tolerance* during the whole study period.

### Glucose restriction plus GAAs suppresses hepatic glycolysis and boosts TCA and gluconeogenesis activity

**Figure 4:**
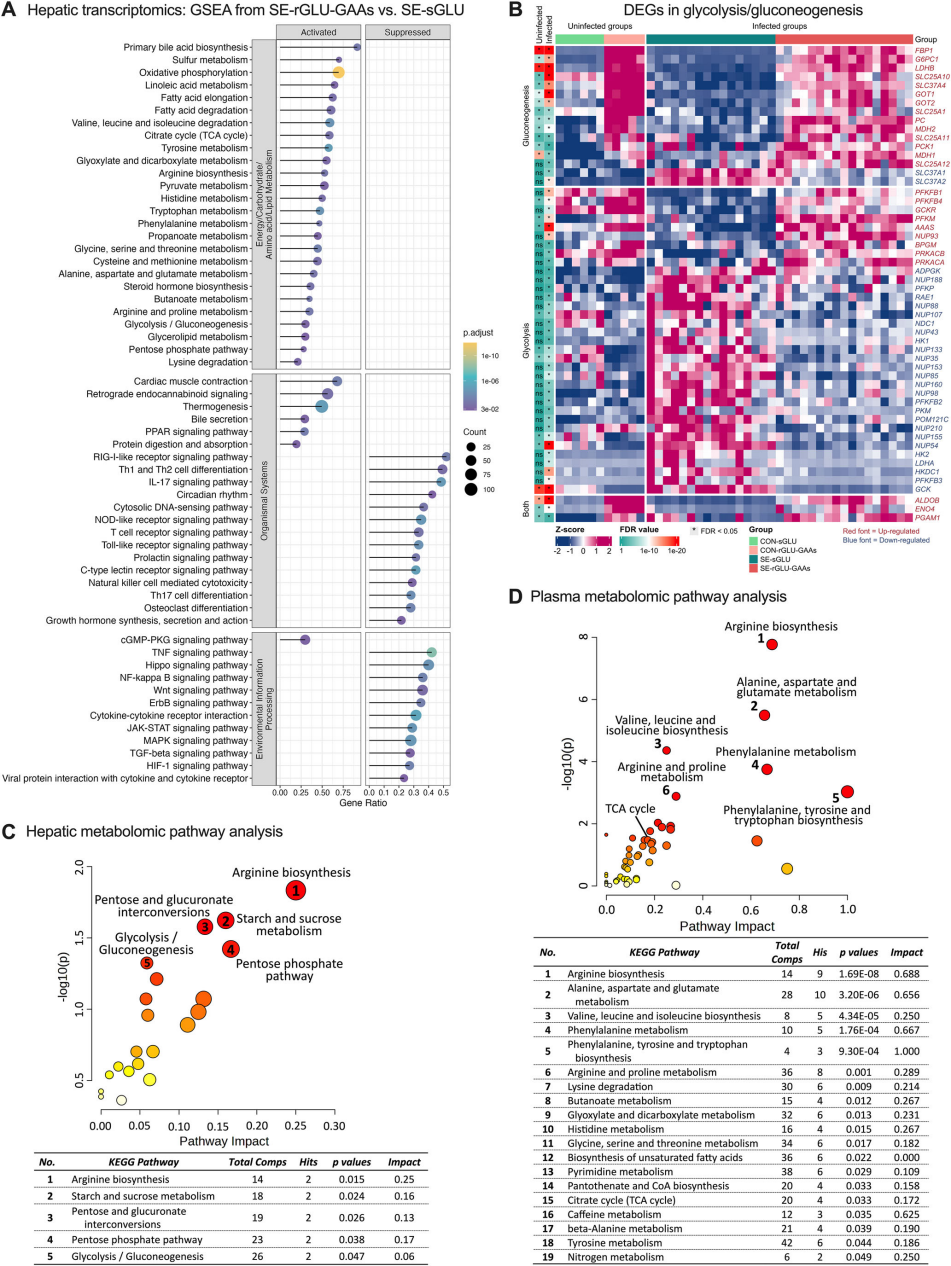
Hepatic transcriptomics and hepatic and plasma metabolomics reveal the link between host metabolism and the sepsis-preventive effects of rGLU-GAAs in preterm piglets. (**A**) Hepatic GSEA comparing SE-rGLU-GAAs and SE-sGLU groups using KEGG pathways for *Sus scrofa*. Selected enriched pathways are shown by category. Dot size and color indicate gene ratio and FDR, respectively. (**B**) Heatmap of 54 hepatically differentially expressed genes (DEGs) in glycolysis/gluconeogenesis pathways between SE-rGLU-GAAs and SE-sGLU groups. (**C**, **D**) MDA-based pathway enrichment in liver tissue and plasma between SE-rGLU-GAAs and SE-sGLU groups. Dot color indicates enrichment significance (red = high, yellow = low), and size reflects pathway impact. Significant pathways are listed in Dataset EV2. In (**A**, **B**), *n* = 15–17/group for infected groups, and *n* = 5–6/group for uninfected groups. In (**C**, **D**), *n* = 10–13/group for infected groups, and *n* = 3–4/group for uninfected groups. Source data are available online for this figure.

We reasoned that the sepsis protective effect of rGLU–GAA is mediated by reduced glycolysis due to glucose restriction and increased TCA cycle activity and gluconeogenesis due to the supplementation of glucogenic amino acids. In the hepatic transcriptome, PCA indicated major impacts of both intervention and infection (Appendix Fig. S4A). GSEA analysis revealed that most metabolic pathways were upregulated by the intervention, regardless of infection status, including OxPhos, TCA cycle, glycolysis/gluconeogenesis, as well as metabolism of amino acids and fatty acids (Fig. 4A; Appendix Fig. S4B and Dataset EV2A,B). In contrast, among infected animals, inflammatory pathways were suppressed by the intervention. DEG analysis showed 3518 upregulated and 3721 downregulated genes by the intervention. Importantly, within 96 genes of glycolysis/gluconeogenesis, 54 genes were regulated, and most genes strictly involved in gluconeogenesis (14/16) were upregulated, whereas most genes strictly involved in glycolysis (26/35) were downregulated in SE-rGLU-GAAs animals (Fig. 4B). Of note, glycolysis was not affected by the intervention in the uninfected condition. This indicates that glycolysis was inhibited by glucose restriction during infection, while GAA supply enhanced gluconeogenesis. Other TCA-related pathways (fatty acid β-oxidation, ketogenesis, OxPhos, and amino acid metabolism), as well as inflammation, also support GSEA findings (Appendix Figs. S4C–F and S5A–H).

**Figure 5:**
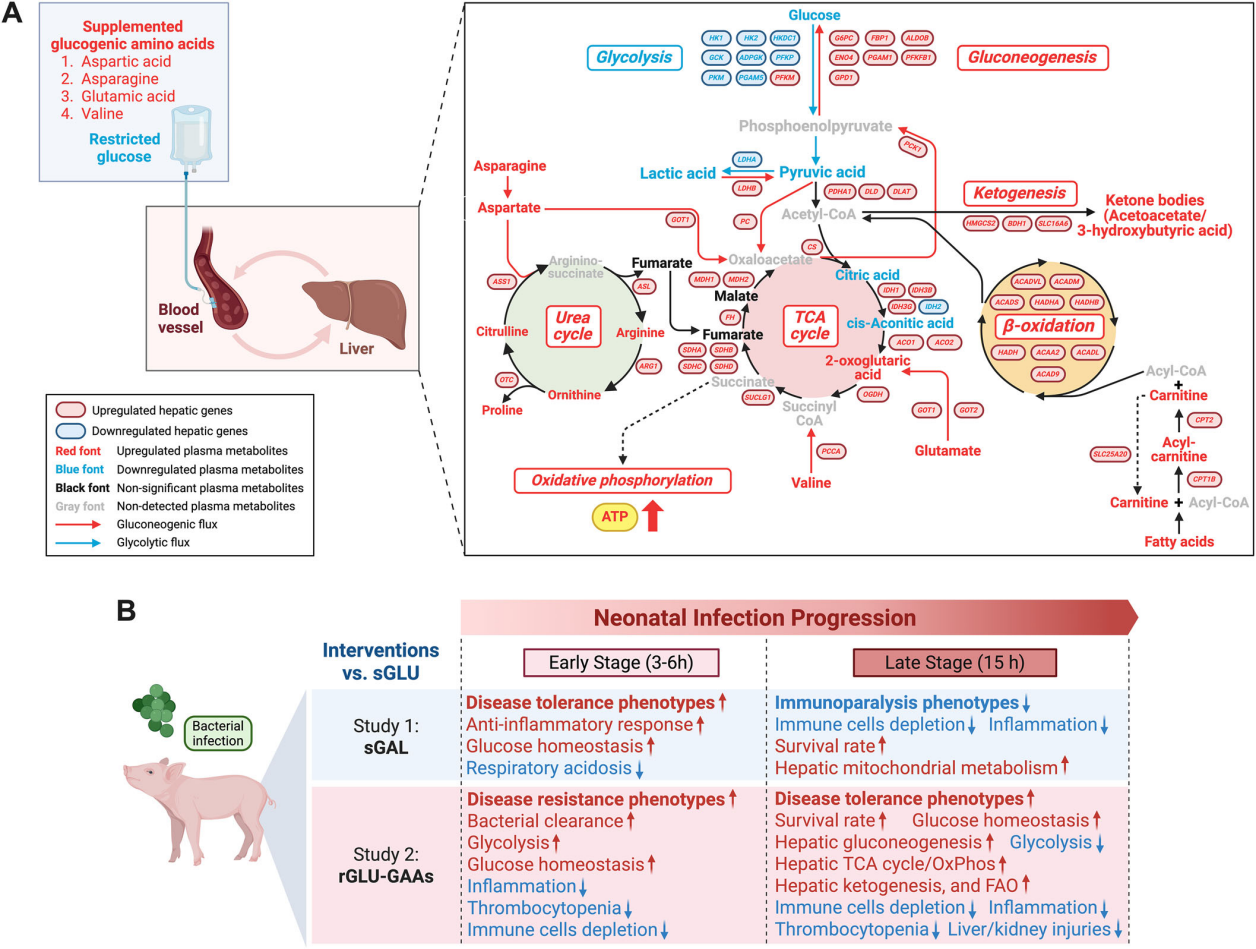
Effects of galactose and glucogenic amino acid supply on systemic metabolism during neonatal infection in preterm piglets. (**A**) Schematic representation of key metabolic alterations in cellular energy pathways (glycolysis, gluconeogenesis, TCA cycle, fatty acid β-oxidation, ketogenesis, and urea cycle) derived from integrated blood parameters, liver transcriptome profiles, and plasma metabolome data in rGLU–GAA vs. sGLU piglets. Pathway visualization is based on genes and metabolites from KEGG knowledgebases, supplemented with additional insights from Reactome knowledgebases and the existing literature. (**B**) Schematic summary of the differential effects of sGAL and rGLU-GAAs across early and late stages of neonatal infection relative to sGLU. Arrows indicate the direction of change relative to the sGLU animals. Source data are available online for this figure.

Next, we sought to confirm metabolic regulations by hepatic and plasma metabolomics. The overall hepatic metabolome with 1014 identified metabolites in 4 experimental groups was clustered together in PCA analysis (Appendix Fig. S6A). Between the two infected groups, 77 metabolites with differential abundance (MDAs) were detected (Dataset EV2C) and were associated with arginine biosynthesis and carbohydrate metabolism, including glycolysis/gluconeogenesis (Fig. 4C; Dataset EV2D). Specifically, carbohydrate-derived metabolites and lactic acid were reduced by SE-rGLU-GAAs, while most of the fatty acids, including carnitines and arachidonic acid derivatives, were upregulated (Appendix Fig. S6B). These support the finding of attenuated glycolysis and increased TCA and OxPhos activities from transcriptomic data. Analysis of plasma metabolome showed a clear clustering between the two infected groups with numerous MDAs (122 increased and 160 decreased by the intervention), and pathway analysis revealed a similar general pattern of metabolic regulations to hepatic transcriptomics and metabolomics (Fig. 4D; Dataset EV2E,F and Appendix Fig. S6C–F). These included elevation of 2-oxoglutaric acid, the metabolite catabolized together with GAAs prior to entering the TCA cycle, as well as elevation of multiple glucogenic and ketogenic amino acids, ketone bodies, carnitine, and fatty acid derivatives. Collectively, data from hepatic transcriptomics and hepatic and plasma metabolomics are in agreement. The sepsis preventive effects of glucose restriction and GAA supplementation were strongly tied to a rewiring of the host energy metabolism, resulting in reduced glycolysis and enhanced TCA cycle, gluconeogenesis, amino acid metabolism, ketogenesis, and fatty acid β-oxidation (Fig. 5A; Appendix Fig. S7). Compared with galactose supply, which primarily supports early disease tolerance and attenuates late immunoparalysis, glucose restriction and GAA supplementation promote early disease resistance and late disease tolerance, resulting in greater overall clinical benefit (Fig. 5B).

### GAA supply alone elevates blood glucose and enhances both *disease resistance* and *tolerance*

**Figure 6:**
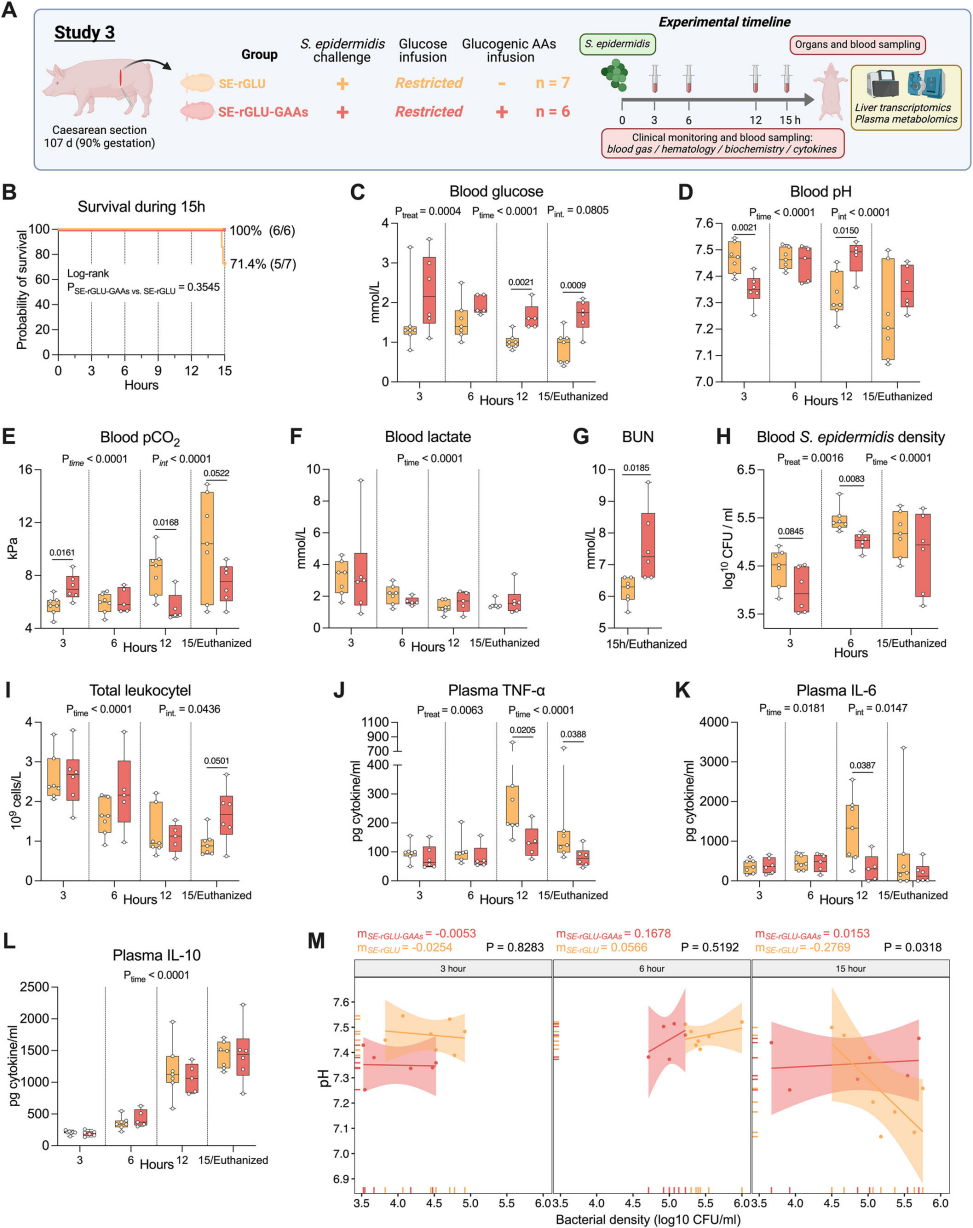
Glucogenic amino acid supplementation is sufficient to modulate clinical parameters and induce metabolic rewiring during sepsis in preterm piglets. (**A**) Animal experimental design. (**B**) Survival curves up to 15 h post-inoculation or humane endpoint, compared via log-rank test. (**C**–**F**) Blood glucose, pH, pCO₂, and lactate levels at 3, 6, 12, and 15 h post-inoculation or at euthanasia. (**G**) Blood urea nitrogen (BUN) at 15 h or euthanasia. (**H**) Blood *S. epidermidis* density at 3, 6 h, or euthanasia, determined by CFU assay. (**I**–**L**) Total leukocyte counts and plasma TNF-α, IL-6, and IL-10 at 3, 6, 12, and 15 h or euthanasia. (**C**–**L**) Data at each time point were analyzed using a linear model, incorporating group, gender, and birth weight as fixed factors. (**C**–**F**, **H**–**L**) Another linear mixed-effects model was employed to probe further disparities spanning the entire experimental duration, incorporating group, time, and their interaction as fixed factors, with pig ID as a random factor. P_treat_, P_time_, and P_int_ denote probability values for group effect (SE-rGLU-GAAs vs. SE-rGLU) over time, time effects, and the interaction effects between time and group in the linear mixed-effects interaction model, respectively. Uninfected animals (CON) served as a reference and were not included in the statistics. Data are shown as box-and-whisker plots. The center line indicates the median; the lower and upper bounds of the box indicate the 25th and 75th percentiles, respectively; and the whiskers extend from the minimum to the maximum values. All individual data points are shown. (**M**) Reaction norm analysis: blood pH vs. pathogen burden at 3, 6, and 15 h. Slopes compared via an extra sum-of-squares F test. *N* = 6–7/group. Source data are available online for this figure.

Testing the rGLU–GAA intervention did not separate the effects of the GAAs from those of glucose restriction alone. Therefore, we conducted a subsequent experiment to assess the impact of GAAs on a glucose-restricted background (Fig. 6A). Glucose-restricted animals developed severe hypoglycemia (mean blood glucose <1 mM), and 2/7 animals died from sepsis. In contrast, GAA-supplemented animals maintained blood glucose concentrations around 2 mM throughout the 15 h study period (Fig. 6B,C), consistent with GAA-induced gluconeogenesis and improved metabolic stability. GAAs also prevented respiratory acidosis (higher blood pH, lower pCO_2_, and unaltered lactate), while increased BUN levels suggest increased amino acid catabolism (Fig. 6D–G). Importantly, GAAs enhanced bacterial clearance at both 3 and 6 h (not at 15 h), blood leukocyte replenishment, and reduced inflammation (lower TNF-α and IL6) at the later phase of the experiment (Fig. 6H–L). Subsequent reaction norm analysis between the two groups showed similar slopes at 3–6 h, but GAA animals again had improved *disease tolerance* at the end of the study (Fig. 6M). Together, this experiment confirmed that GAAs directly contributed to the clinical benefits of the rGLU–GAA intervention, most likely via enhancement of both *disease resistance* and *tolerance*, as well as improved hepatic gluconeogenesis. Importantly, while our earlier research established a link between hyperglycemia and heightened sepsis severity (Bæk et al, 2024; Muk et al, 2022) the current findings indicate that avoidance of severe hypoglycemia also contributed to sepsis prevention. The higher glucose levels in treated animals were not exogenous but stemmed from gluconeogenesis induced by supplying GAA during glucose restriction.

**Figure 7:**
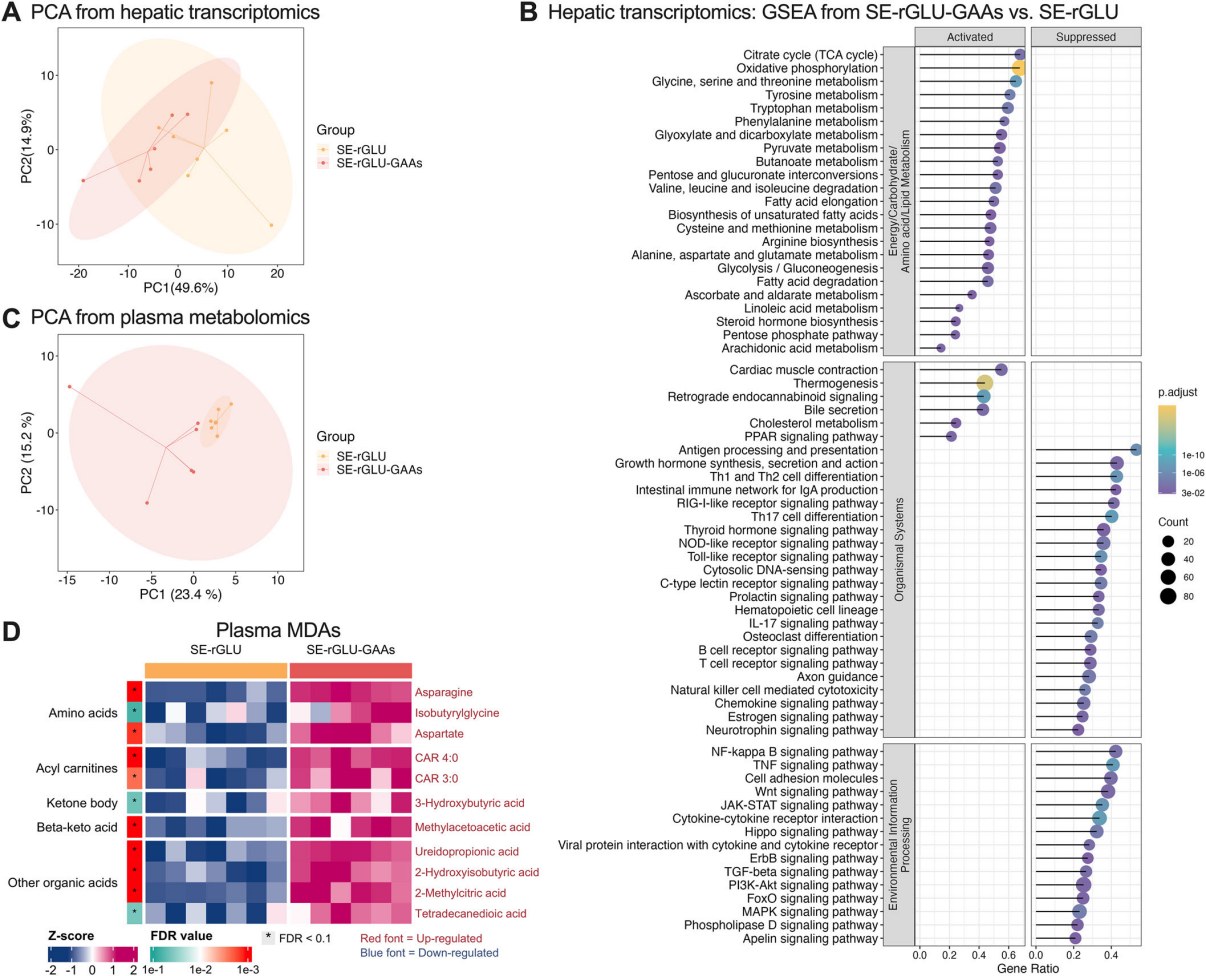
GAA supply during glucose restriction enhances gluconeogenesis and reduces inflammation during neonatal infection in preterm piglets. (**A**, **C**) PCA plots of hepatic transcriptomics and plasma metabolomics, showing scores for the first two principal components. (**B**) GSEA comparing SE-rGLU-GAAs and SE-rGLU groups using the *Sus scrofa* KEGG database. Selected enriched pathways are shown across various categories. Dot size and color represent gene ratio and FDR, respectively. (**C**) Heatmap of differentially expressed genes (DEGs) related to energy metabolism between SE-rGLU-GAAs and SE-rGLU groups. (**D**) Heatmap of key MDAs (metabolites with differential abundances) between SE-rGLU-GAAs and SE-rGLU groups. *N* = 6–7/group. Source data are available online for this figure.

Finally, we also sought to confirm the GAA effects via hepatic transcriptomics and plasma metabolomics. A distinct hepatic transcriptome profile between the two groups was shown by both PCA (Fig. 7A) and DEG analysis, with 189 upregulated and 136 downregulated genes in the SE-rGLU-GAAs group. From both GSEA and DEG analyses, GAAs upregulated OxPhos, TCA cycle, glycolysis/gluconeogenesis, amino acid and lipid metabolic pathways, while dampening several inflammatory pathways (Fig. 7B; Appendix Fig. S8A,B and Dataset EV3A). For plasma metabolomics, PCA also revealed distinct clustering patterns between the two groups (Fig. 7C), while MDA analysis showed increased levels of most amino acids, acylcarnitines, ketone bodies, keto acids, and fatty acids in infected rGLU-GAAs animals (Fig. 7D; Dataset EV3B). It was clear that in infected animals, the rGLU-GAAs vs. sGLU comparison (study 2) had much more profound changes in hepatic transcriptome and plasma metabolome than the rGLU vs. rGLU comparison (study 3). Regardless, the additive effects of GAAs in the background of glucose restriction on *disease resistance* and *tolerance*, as well as the TCA cycle, gluconeogenesis, and systemic inflammation, appeared to be critical to both sepsis prevention and glucose homeostasis.

### GAA score connection with infection burdens and TCA score in human infants

**Figure EV2:**
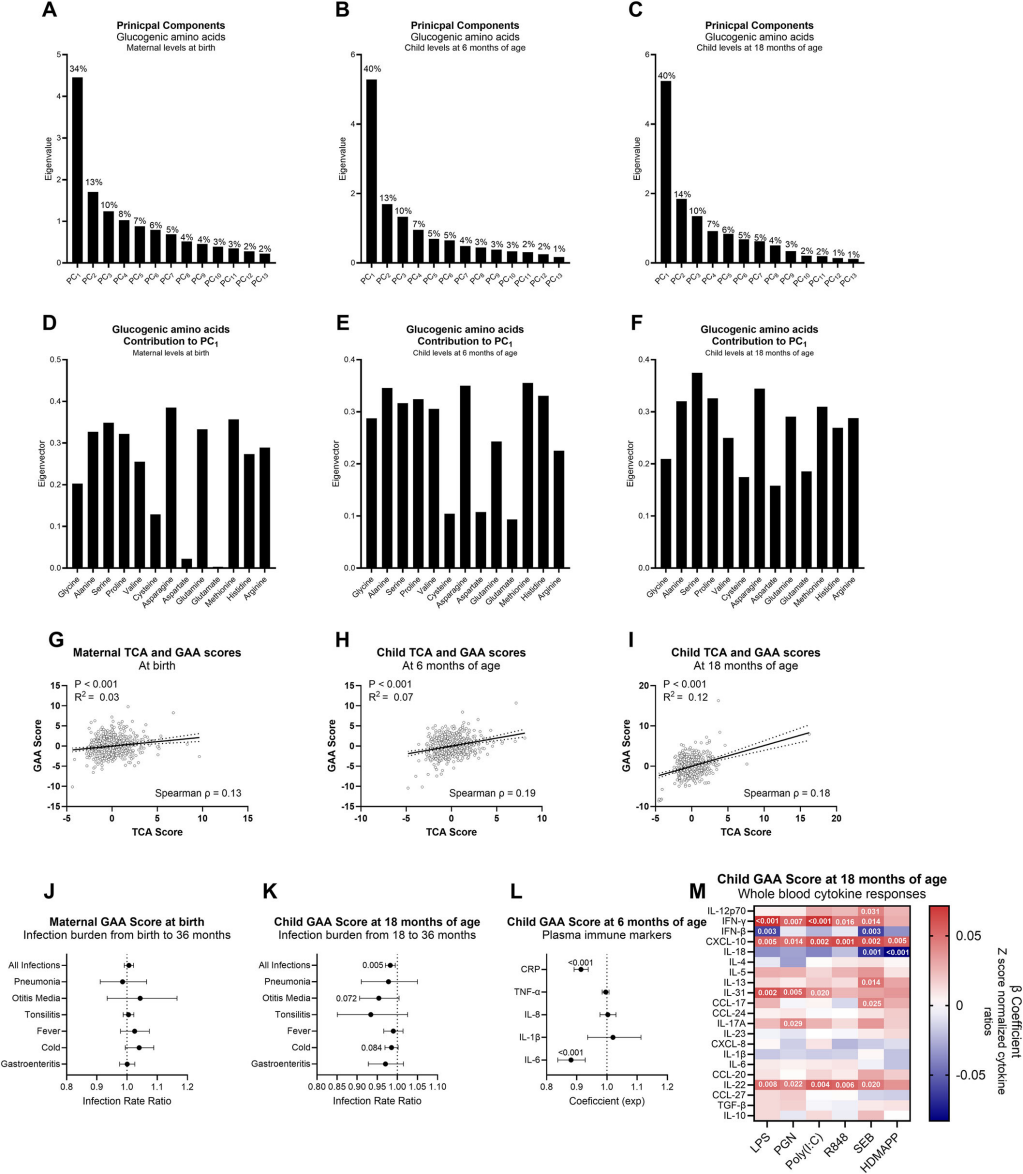
Correlation of TCA and GAA scores and association of GAA score and infection burdens and inflammation in infants. (**A**–**C**) Eigenvalues showing the fraction each principal component (PC) contributes to the overall PCA at birth, 6, and 18 months of age for GAAs. (**D**–**F**) Eigenvectors showing how each TCA metabolite contributes to PC_1_ (TCA scores) at birth, 6, and 18 months of age. (**G**–**I**) Spearman correlation between TCA and GAA scores at birth, 6 and 18 months of age. (**J**, **K**) Association of GAA scores at birth and 18 months with infection burdens from birth to 36 months and 18-36 months, respectively. Both are shown as estimated risk ratios with 95% CIs, derived from quasi-Poisson regression. (**L**) Associations between 6-month GAA scores and plasma inflammatory markers. CRP: C-reactive protein, TNF: Tumor necrosis factor, IL: Interleukin, analyzed by generalized linear model shown as estimated β-coefficients with 95% CIs. (**M**) Heatmaps showing the association between individual GAA scores at 18 months of age and z-score normalized cytokine ratios following whole blood stimulation with 6 pattern recognition receptor agonists. Shown as β-coefficients following a generalized linear model where red color indicates positive, and blue negative association. LPS lipopolysaccharide, PGN peptidoglycan, R848 imidazoquinoline, SEB staphylococcal enterotoxin B, HDMAPP 1-Hydroxy-2-methyl-2-buten-4-yl 4-diphosphate. Exact *P* values were indicated in each figure panel. *n* = 678 for maternal data, *n* = 562 and 538 for data at 6 and 18 months, respectively.

Upon the strong effects of GAAs in animal model, we finally returned to confirm the role of GAAs in human infants. We created a GAA-score based on a principal component analysis of plasma levels of all 13 glucogenic amino acids in available plasma metabolomic data from COPSAC2010 cohort, at birth, 6 and 18 months. At each timepoint, PC1 accounted for 34–40% of the variation, and all GAAs contributed positively to it (Fig. EV2A–F). Further, the GAA scores were correlated significantly with corresponding TCA scores, both at birth, 6 and 18 months (Fig. EV2G–I, all *P* < 0.001). Maternal GAA score at birth was not associated with altered infection risk (Fig. EV2J). However, child GAA scores at 6 months were associated with lower levels of plasma CRP and IL-6, while child GAA scores at 18 months of age were associated with lower risk of all infections from 18 to 36 months, and higher whole blood IFN-γ, CXCL-10 and IL-22 immune responses (Fig. EV2K–M).

## Discussion

The aim of this study was to identify nutritional strategies that improve infection outcomes in vulnerable newborns. By integrating human cohort data, in vitro experiments, and translationally relevant neonatal pig studies, we demonstrate that host energy metabolism, particularly TCA cycle activity, offers a promising target for nutritional interventions to strengthen infection defense in early life. Data from the COPSAC_2010_ mother–child cohort indicate that normal variation in TCA cycle activity was associated with altered risk of common bacterial infections in childhood (otitis media, tonsillitis, and cold symptoms), systemic inflammation, and immune functions. These findings are in alignment with previous literature and indicate that activity of the TCA cycle not only correlates with anti-inflammatory/tolerogenic immune responses, but also better infection resilience (Liu et al, 2020; Trauelsen et al, 2021; Dom**í**nguez-Andrés et al, 2019). Importantly, the metabolites detected in plasma reflect the metabolism of the entire host, not just the immune system. Global changes in energy metabolism may therefore have an impact on the metabolism and responses of immune cells. However, the association between energy metabolism and immunity may be more complex in those born prematurely or at a low birthweight. In hospitalized neonates, hypoglycemia is among the most common metabolic disturbances shortly after birth, which is partly derived from their low glycogen stores (Abramowski et al, 2023). On the other hand, premature newborns frequently develop hyperglycemia due to insulin resistance when given glucose-rich PN, increasing the risk that infections escalate to sepsis (Salis et al, 2017; Hofman et al, 2004). This metabolic instability highlights the need for nutritional approaches that strengthen mitochondrial TCA cycle activity, maintain energy availability during infections, while preventing the glycolysis-driven excessive inflammation that contributes to poor outcomes (Wu et al, 2024; Muk et al, 2022; Bæk et al, 2024).

Using a translational neonatal sepsis model, we identified two nutritional approaches, galactose and GAA supplementation, that boost TCA cycle activity and improve infection survival through effects on host defense mechanisms. Despite being a natural part of lactose in breastmilk, and released in the gut and absorbed after breastfeeding, galactose has not been used as a carbohydrate source for newborn infants. Immune cells cultured in galactose-containing medium showed suppressed glycolytic activity and lactate production (Zhang et al, 2019), as it must first be metabolized before entering glycolysis or released as glucose (Conte et al, 2021). In the current study, replacement of PN glucose with galactose enhanced *disease tolerance* during the early phase of infection and overall anti-inflammatory response over the course of the infection and prevented hyperglycemia, improving survival and clinical outcomes. In the galactose-treated group, a few animals exhibited transient low-glucose values at the 3–6 h time points. These episodes were infrequent, short-lived, and not accompanied by clinical signs of hypoglycemia. However, important markers of liver and kidney injuries (liver enzymes, albumin, cholesterol, and creatinine) were minimally affected, together with limited changes in hepatic metabolic rewiring measured by transcriptomics at the end of the study. It thus appeared that substituting galactose for glucose still resulted in some downstream glycolytic activity, delaying rather than preventing the progression of excessive inflammation. In clinical settings, the sepsis delaying effects of the galactose intervention may provide an important window of opportunity for other adjunct therapies (e.g., antibiotics or inotropes) in infected, vulnerable newborns prior to clinical deterioration.

In contrast, the alternative approach of combining glucose restriction and GAA supplementation effectively limited glycolysis and enhanced TCA cycle activity as well as gluconeogenesis. This markedly improved infection survival and protection against organ damage. The metabolism of GAAs via the TCA cycle can provide energy via OxPhos for the liver and immune system, affecting their inflammatory response, and via hepatic gluconeogenesis, help maintain a supply of glucose for other vital organs. Preterm infants do have a reduced capacity for gluconeogenesis, but nonetheless express the required enzymes for gluconeogenic activities (Kalhan et al, 2001; Chacko and Sunehag, 2010). So, it is possible that glucose homeostatic control during neonatal infection in preterm infants can be achieved by restricting PN glucose supply and GAA supplementation. Importantly, this intervention did not completely inhibit inflammation, and the treated animals were capable of mounting a sufficient *disease resistance* response to better eliminate invading bacteria during the first six hours of infection. Strikingly, *disease tolerance* mechanisms were also enhanced by the intervention at six hours and maintained until the end of the study. Clearly, infection survival was not correlated with the host capacity to eliminate invading pathogens but rather dictated by the balance of host defense strategies. To our knowledge, this was the first study showing simultaneous enhancement of both *disease tolerance* and *resistance* during infection. Enhanced *disease tolerance* exerted by glucose restriction and GAAs was also associated with improved hepatic energy metabolic pathways, not only the TCA cycle and OxPhos but also fatty acid oxidation, and ketogenesis. Whether these pathways are affected downstream from the TCA cycle, or the other way around, is not clear from our data. However, these pathways have been shown to play critical roles in providing alternative energy for vital organs (e.g., brain and heart) and protecting against inflammation-induced tissue damage during sepsis (Willmann and Moita, 2024). Further, ketone bodies, particularly beta-hydroxybutyrate, can exhibit potent anti-inflammatory effects (Zhao et al, 2023; Youm et al, 2015). Finally, all these effects are strikingly similar to the regulated metabolic pathways and defense strategies we have previously demonstrated in preterm pigs that survive infection (Wu et al, 2024).

It is critical to note that galactose substitution and GAA supplementation were used as a proof-of-concept to test whether glucose levels can be maintained during infections when glucose supply is markedly reduced. The extreme glucose restriction was chosen to reveal mechanistic effects, not to mirror clinical dosing, so the results should not be viewed as directly translatable. Instead, they show that GAAs can sustain gluconeogenesis under low-glucose conditions, supporting future studies using clinically relevant glucose infusion rates. We propose that a PN formulation in which part or all of the glucose is replaced with galactose and GAAs may prevent hyperglycemia and, importantly, reduce the risk of sepsis and adverse infection outcomes in neonates. To be effective, such a strategy would likely need to be initiated from birth, prior to infection onset, in order to support early immune responses and shape the initial host–pathogen interactions.

In conclusion, our results not only strongly imply connections between newborn metabolic regulation and host infection defense but also suggest potential therapies that may be lifesaving for vulnerable newborns with suspected infections. Both galactose and glucose restriction plus GAA supplementation conferred protective effects during serious bloodstream infections and modulated newborn defense strategies. Given the obvious safety of galactose, phase 1 trials in vulnerable newborns requiring monosaccharide infusion may be the best way to determine if human newborns are capable of utilizing galactose effectively, either alone or in some combination with glucose. Our encouraging results may directly pave the way for clinical trials testing the feasibility and efficacy of these interventions.

### Limitations of the study

With the human cohort data, it was only feasible to establish associations, but not causation, between semi-quantitative levels of plasma metabolites and infection burden. Further, the granular nature of the data allowed the distinction between suspected bacterial and viral infections, although it must be noted that the cohort comprised healthy children and thus had a very low rate of serious infections requiring hospitalization. It was therefore not ethically feasible to collect plasma from the newborn infants, necessitating the use of maternal plasma metabolomics at birth. However, we have previously demonstrated that metabolite levels at birth closely track between mother and infant (Brustad et al, 2023; Olarini et al, 2022). Further, certain limitations of animal studies must be acknowledged. First, the preterm piglet provides a clinically relevant system for studying neonatal immune–metabolic responses, yet our sepsis model involves *S. epidermidis* infection shortly after birth, reflecting early postnatal exposure. While this bacterial species is a major cause of late-onset sepsis in preterm infants, it is less frequently implicated in early-onset sepsis. Thus, the clinical phenotype replicated here aligns more closely with late-onset nosocomial infection, and metabolic states in this context may differ from those during the immediate postnatal adaptation phase. Second, different bacterial pathogens may elicit distinct host-defense and metabolic strategies, and additional models incorporating other neonatal pathogens will be valuable to validate the broader applicability of our findings. Third, due to resource constraints and the limited volume of blood collected during the study, a more detailed characterization of host defense, immune, and metabolic responses at various time points during infection was not possible, limiting a comprehensive understanding of host metabolic changes over time. Since we focused on the effects of four selected strictly glucogenic amino acids, the mechanistic impacts of each amino acid in this setting are unknown. Fourth, only birthweight was recorded, while bodyweight changes over time of infection were not measured. Finally, it is important to emphasize that the experimental PN formulations were not intended to be directly clinically translatable but were tailored to the energy and amino acid requirements of preterm pigs rather than human infants. Accordingly, all experiments should be interpreted as proof-of-concept.

## Methods

**Table:** Reagents and tools table

Reagent or resource	Source	Identifier
**Bacterial strain**		
*Staphylococcus epidermidis*	Isolated from a sepsis infant	WT-1457
**Cell line**		
THP-1 cell	ATCC	Tib-202
**Chemicals and reagents**		
D-(+)-Galactose	Sigma	G5388
L-Aspartate	Sigma	A7219
L-Asparagine	Sigma	A4159
L-Valine	Sigma	V0513
L-Glutamate	Sigma	G8415
Doxapram	Dechra	34144/G
Flumazenil	Hameln Pharma	33659
Zoletil 50 Vet.	Virbac	568527
Xysol Vet	CP Pharma	CP 054899
Ketamine (Ketaminol Vet	Merck	511519
Pentobarbital	Alfasan	088672
Kabiven	Fresenius-Kabi	052505
Heparin	Panpharma	ETISM788-1
**Critical commercial assays**		
ATP Assay Kit	Sigma-Aldrich	MAK190
Porcine TNF-α DuoSet ELISA Kit	R&D	DY690B
Porcine IL-6 DuoSet ELISA Kit	R&D	DY686
Porcine IL-10 DuoSet ELISA Kit	R&D	DY693B
RNeasy mini kit	Qiagen	74106
Human TNF alpha Uncoated ELISA Kit with Plates	ThermoFisher	88-7346-22
**Experimental models: organisms/strains**		
Preterm piglets	crossbred, Landrace × Yorkshire × Duroc	
**Deposited data**		
RNA sequencing data	NCBI	GEO: GSE263512
**Software and algorithms**		
GraphPad Prism 9	GraphPad Software	https://www.graphpad.com/ scientific-software/prism/
R version 4.2.3	R Foundation for Statistical Computing	https://www.r-project.org/
MetaboAnalystR 6.0	Metaboanalyst	https://www.metaboanalyst.ca/
**Others**		
GEM Premier 3000	Instrumentation Laboratory	GEM Premier 3000
ADVIA 2120i Hematology System	Siemens	ADVIA 2120i System
ADVIA 1800 Chemistry System	Siemens	ADVIA 1800 System

### Human data

#### Study population

The studied cohort involved both female and male infants, and sex was included as a covariate in statistical models. The Danish population-based COPSAC_2010_ mother–child cohort has been comprehensively described previously, including detailing participant baseline characteristics and the enrollment process (Chawes et al, 2016). Briefly, 736 pregnant women were recruited at 24 weeks of gestation, and their children were monitored longitudinally at the COPSAC research clinic until the age of 3 years, with extended follow-up visits at 4, 5, 6, 8, 10, and 13 years. This included nine planned visits at age 0–3 years as well as acute care visits, with a primary focus on common infections and atopic conditions. The pregnant women participated in a double-blind randomized controlled trial using a 2 × 2 factorial design, receiving either fish oil capsules containing 2.4 g of n-3 long-chain polyunsaturated fatty acids or a placebo (Clinicaltrails.gov: NCT00798226). In addition, 623 participants were enrolled in another RCT comparing high-dose vitamin D supplementation (2800 IU/day) with a standard dose (400 IU/day) (Clinicaltrails.gov: NCT00856947). The study received approval from the Danish Ethics Committee (H-B-2008-093) and the Danish Data Protection Agency (2008-41-2599). Infections were recorded through acute visits and daily diaries during the first 3 years and included pneumonia, tonsillitis, acute otitis media, gastroenteritis, cold symptoms, and fever. Diagnoses were confirmed by COPSAC physicians at scheduled and acute care visits based on parental interviews, medical records, and registry data (Brustad et al, 2025). Informed consent was obtained from all subjects, and the study conformed to the principles set out in the WMA Declaration of Helsinki and Department of Health and Human Services Belmont Report. All metabolomics, whole blood stimulation assays, and immune profiling have been performed and published previously. In this study, we analyzed the available data of the cohort for association with TCA metabolites and infection/immune outcomes. All details for these analyses were briefly described below with reference to the original studies.

#### Metabolomics profiling

Metabolite data were planned prospectively to be obtained from plasma samples collected from the mother at the time of birth and from the child at 6 and 18 months of age, when a routine health check was performed. LC-MS metabolite characterization was performed by Metabolon, Inc. (Durham, NC, USA). A detailed analytical method was described previously (Rago et al, 2021, 2019). The identification level followed the reported criteria (Sumner et al, 2007).

#### Immune profiling

At 18 months of age, available heparinized blood was collected and analyzed within 4 h for flow cytometry and ex vivo immune stimulation. All data have been generated and published previously (Thysen et al, 2020). Briefly, for flow cytometry, cell subsets were defined based on specific marker combinations and side scatter (SSC) properties as follows: granulocytes (SSC^int/high^), neutrophils (CD16^+^ SSC^int/high^), eosinophils (CD16^-^ SSC^high^), B cells (CD19^+^), T cells (CD3^+^), CD4 T cells (CD3^+^CD4^+^), CD8 T cells (CD3^+^CD8^+^), activated CD4 T cells (CD3^+^CD4^+^CD25^+^CD127^high/+^), activated CD8 T cells (CD3^+^CD8^+^CD25^+^CD127^high/+^), Tregs (CD3^+^CD4^+^CD25^+^CD127^low/−^), γδT cells (CD3^+^γδTCR^+^), invariant NKT cells (iNKT, CD3^+^Vβ24Jα18^+^), classical monocytes (CD3^-^CD19^-^CD14^+/high^CD16^−^), intermediate monocytes (CD3^-^CD19^-^CD14^+/high^CD16^+^), inflammatory monocytes (CD3^-^CD19^-^CD14^int/−^CD16^+^), BDCA-1 dendritic cells (CD3^-^CD19^-^CD14^-^CD16^-^BDCA-1^+^), BDCA-3 dendritic cells (CD3^-^CD19^-^CD14^-^CD16^+^CD1c^-^BDCA-3^high^), plasmacytoid dendritic cells (CD3^-^CD19^-^CD14^-^BDCA-2^+^), NK cells (CD3^-^CD56^+^), CD56^dim^ NK cells (CD3^-^CD56^dim^), and CD56^bright^ NK cells (CD3^-^CD56^bright^). Total counts of leukocytes, monocytes, dendritic cells, innate immune cells, and adaptive immune cells were also enumerated.

Immune functional tests were performed in whole blood, and data treatment was reported previously (Thysen et al, 2020). Briefly, blood was stimulated with one of the following agents (37 °C and 5% CO₂ for 24 h): *E. coli*-derived lipopolysaccharide (LPS, InvivoGen), synthetic dsRNA analog Poly(I:C), imidazoquinoline, peptidoglycan (PGN,), 1-Hydroxy-2-methyl-2-buten-4-yl 4-diphosphate (HDMAPP), and staphylococcal enterotoxin B (SEB). Cytokine and chemokine concentrations were quantified in supernatant using MesoScale Discovery (MSD) pre-coated plates, including Human IFN-beta Tissue Culture Kit, Human TGF-β1 Kit, Cytokine Panel 1 V-PLEX, Prototype Human 3-plex, and MULTI-SPOT 10 Spot Special Order Human 9-plex. Undetectable levels were assigned values at half the minimum detectable concentration for each analyte. Cytokine stimulation ratios were calculated by dividing the stimulated value by the unstimulated value, and all values were then z-score transformed before analysis.

#### Statistics

Infection risk was analyzed by a quasi-Poisson regression model estimating the incidence risk ratio using the number of infection episodes as count data. Time to infection was calculated by a Cox proportional hazard model, while the immune data were analyzed by a general linear regression model estimating beta-coefficients using the continuous cell count or cytokine levels. Analyses from birth, were adjusted for possible confounders such as the randomization in the two RCTs, sex, social circumstances, smoking during pregnancy, gestational age, birthweight, delivery mode, neonatal hospitalization, maternal antibiotic use during pregnancy, maternal asthma, birth season, maternal age, BMI, duration, and presence of siblings while at 6 and 18 months covariates were reduced to participation in RCTs, sex, social circumstances, child BMI, and any infections 14 days previously, based on directed acyclic graphs and previous similar analysis on the impact of metabolite levels at birth on infection outcomes (Brustad et al, 2023). Analyses were conducted using Stata (version 14.2), with significance defined as *P* < 0.05.

### In vitro experiments

The human monocyte-like THP-1 cell line was obtained from ATCC (TIB-202) and cultivated in RPMI-media (ThermoFisher 72400047) with 10% FCS (ThermoFisher A5670502). The cell line has been tested by ATCC with confirmation of no mycoplasma contamination. Prior to stimulation, cells were seeded in 24-well plates, 2.5 × 10^5^ cells/well, and differentiated into macrophage-like cells by treatment with Phorbol-12-myristate-13-acetate (Sigma, 524400, 180 nM) for 3 days. Following differentiation, cells were infected with *S. epidermidis*, MOI 0.5 for 4 or 6 h, with and without treatment with glucose (10/50 mM), galactose (10/50 mM), asparagine (2 mM), aspartate (2 mM), glutamate (2 mM), valine (2 mM), or an amino acid mixture (2/8 mM). Afterward, intracellular ATP was determined using the BacTiter Glow ATP assay (Promega, 74106), and cytokine secretion was determined using commercial ELISA kits (ThermoFisher 88-8086-86, 88-7106-86, 88-7066-86) according to the manufacturer’s instructions.

Total RNA was isolated using the RNeasy kit according to the manufacturer’s instructions (Qiagen 7404). cDNA was synthesized from 2 µg of RNA using a High-Capacity cDNA Reverse Transcription Kit (Thermo Fisher, Waltham, MA, USA). Real-time quantitative PCR (RT–qPCR) was performed with 10-fold diluted cDNA using the LightCycler 480 SYBR Green I Master kit on a LightCycler 480 (both Roche, Basel, Switzerland). Samples were analyzed in duplicate using cytokine-specific primers (Dataset EV4). Gene expression was normalized to the expression of beta-actin using the 2-△△CT method. Data was normalized to *S. epidermidis* infected cells cultured in uncomplemented media, and all statistical comparisons between treatments were done by either a paired T-test (for normally distributed data) or Wilcoxon’s signed-rank test. Analyses were conducted using Stata (version 14.2), with significance defined as *P* < 0.05.

### Experimental animal studies

#### Animals

Animal studies involved both female and male animals, and sex was used as a covariate in statistical models. Seventy-four crossbred preterm piglets (Landrace ×  Large White × Duroc) were delivered by cesarean section at day 106 (90% gestation, term at day 117) from three healthy pregnant sows (21, 23, and 27 piglets per sow). The anesthesia, surgical procedures of sows, and housing conditions for sows and piglets are described in detail elsewhere, which were in compliance with the Danish ethical regulations (Bæk et al, 2020). After delivery, the newborn piglets were immediately transferred to preheated (37 °C) newborn incubators with a supplementary oxygen supply (1–2 L/min). When needed, animals were resuscitated by Doxapram and Flumazenil (0.1 ml/kg for each, intramuscular injection), tactile stimulation, and manual noninvasive positive pressure ventilation, applied as necessary until achieving respiratory stability. Furthermore, each piglet was equipped with a vascular catheter inserted into the dorsal aorta via the transected umbilical cord, facilitating the administration of PN, bacterial inoculation, and blood sampling. Three piglets were euthanized due to failure of jugular vein puncture, catheter problem, and unsuccessful resuscitation, and were excluded from the study.

#### *S. epidermidis* culture preparation

Originating from frozen stock, the *S. epidermidis* bacteria, isolated from a septic infant, were cultured in tryptic soy broth overnight. Following this incubation, bacterial density was accurately gauged using spectroscopic techniques. Based on these measurements, a working solution (10^9^ CFU/kg) was formulated by diluting the culture with a precise volume of sterile saline (Bæk et al, 2020).

#### Parenteral nutrition preparation

Four specialized formulations of parenteral nutrition (PN) were developed from the Kabiven infusion formula (Fresenius-Kabi). The Kabiven system consists of three chambers that provide amino acids, glucose, and lipid emulsion. For PN with standard glucose (sGLU) provision, the glucose chamber was emptied, and a specific volume of 50% glucose solution was added into the glucose chamber to reach 10% glucose PN with infusion rate of 6 mL/kg/h, equivalent to 14.4 g/kg/d glucose, similar to PN used for preterm infants (Mesotten et al, 2018b) For PN with galactose supply with standard glucose equivalent level (sGAL), the glucose chamber was emptied, and galactose solution was added to reach 10% galactose PN (14.4 g/kg/d). For glucose-restricted (rGLU) PN, a precise volume of glucose was removed from the PN glucose chamber to reach a final glucose content of 1.4% (2 g/kg/d with an infusion rate of 6 mL/kg/h). For PN with glucose restriction and supplementation of four glucogenic amino acids (rGLU-GAAs), a precise volume of four glucogenic amino acids solution was added to the glucose-restricted PN solution to reach the infusion rate of 0.5 g/kg/d for each amino acid (aspartate, glutamate, asparagine, valine).

#### Animal experimental procedures and intervention

Approximately 2 h after the cesarean section, all animals were randomly stratified based on birth weight and sex into groups receiving one of the four types of PN: sGLU, sGAL, rGLU, and rGLU-GAAs. The treatment group was not blind to the investigators. Animals were then inoculated with live *S. epidermidis* (SE) or control saline via an interatrial infusion over three minutes. During the post-inoculation period, animals reaching predefined humane endpoint (lethal sepsis criteria, including arterial blood pH of ≤7.1 and clinical signs of deep lethargy, discoloration, and tachypnea) were euthanized for blood sampling and tissue collection. To standardize sample collection times and improve animal welfare, we adjusted the experimental duration for the second and third litters to 15 h. This modification was prompted by initial findings where animals in the first litter received intensive care for up to 20 h post-inoculation. Animal numbers in infected groups are estimated based on effect size detected from previous studies (Muk et al, 2022; Bæk et al, 2024). Predefined criteria to exclude animals were respiratory failures and iatrogenic complications after preterm birth. Group allocation is not blinded to animal caretakers.

Blood samples were collected at 3, 6, and 12 h post-bacterial challenge and at the study end via the arterial catheter for blood gas analysis and hematology, as well as plasma collection and storage for cytokine measurements. At 3 and 6 h and at the study end, blood samples were drawn through jugular vein puncture for bacterial enumeration. Serum and plasma samples at euthanasia were used for serum biochemical analysis and plasma metabolomic analysis.

At humane endpoint or 15 h of post-inoculation, all piglets were deeply anesthetized with a Zoletil mixture (0.1 ml/kg), which included Zoletil 50 (125 mg tiletamine, 125 mg zolazepam), xylazine (6.25 ml xylazine 20 mg/ml), ketamine (1.25 ml ketamine 100 mg/ml), and butorphanol (2.5 ml butorphanol 10 mg/ml), and were subsequently euthanized with an intracardiac injection of barbiturate. The liver was collected and preserved (frozen) during necropsy for liver transcriptomic and metabolomic analysis.

#### Blood gas, hematology, inflammatory markers, serum biochemistry, and plasma ATP measurement

Blood samples at 3, 6, 12, and 15 h underwent routine blood gas analysis utilizing the GEM Premier 3000 (Instrumentation Laboratory, USA) for blood pH, pCO_2_, oxygen saturation, base excess, glucose, and lactate measurement. Hematological assessments were conducted with the ADVIA 2120i Hematology System (Siemens, Germany). For the quantification of plasma cytokines, TNF-α, IL-6, and IL-10 were analyzed using porcine-specific DuoSet enzyme-linked immunosorbent assays (R&D Systems). Serum biochemistry evaluations were executed using the ADVIA 1800 Chemistry System (Siemens, Germany). In addition, the extracellular ATP level was measured by the ATP Colorimetric Assay Kit (Sigma-Aldrich).

#### Hepatic transcriptome profiling and analysis

RNeasy mini kit (QIAGEN, USA) was used to extract liver RNA from all piglets. Gene expression was profiled by utilizing whole-transcriptome shotgun sequencing. Library preparation and sequencing were carried out by NOVOGENE services (Cambridge, UK). DEG analysis was performed using the *DESeq2* package version 1.38.3 (Love et al, 2014). We excluded lowly expressed genes, defined as having a raw count of less than 10 in 4, 5, and 6 samples in Experiments 1, 2, and 3, respectively. Litter was added to adjust the model. To robustly estimate the fold change (FC), we applied the *lfcShrink* function with the *ashr* estimator (Zhu et al, 2019). A false discovery rate (FDR) cut-off of 0.05 was implemented to obtain DEGs. Genes were ranked by the FC (in log scale) before pathway analyses. The gene set enrichment analysis (GSEA) module in the *clusterProfilter* package version 4.0.2 (Wu et al, 2021) was utilized with *Sus scrofa* Kyoto Encyclopedia of Genes and Genomes (KEGG) knowledgebases to elucidate pathway-level disturbances. Down- or upregulated pathways were defined based on a normalized enrichment score (NES) with negative or positive values, respectively. The FDR cut-off of 0.05 was considered to determine significant pathways. To create a comprehensive visualization of specific genes within GSEA-enriched pathways, DEGs were identified based on the KEGG, Biological Processes aspect of Gene Ontology (GO:BP), Reactome, and Hallmark knowledgebases, with additional insights from the literature. The relative expression of DEGs was then visualized through heatmaps generated using the *ComplexHeatmap* package version 2.15.1 (Gu et al, 2016). The analysis of liver transcriptomics data was performed in R 4.2.3.

#### Hepatic and plasma metabolome profiling and analysis

Untargeted metabolomics analysis was conducted using ultra-performance liquid chromatography-mass spectrometry (UPLC-MS) through the services provided by Creative Proteomics (Shirley, NY, USA), with detailed methods described previously (Wu et al, 2024). For the hepatic metabolome, there were 5614 and 5067 features in positive and negative ion modes, respectively. Among them, 1098 putatively annotated features, assigned by the vendor and cross-examined by our team, were eventually used for subsequent analyses. For the plasma metabolome, a total of 754 putatively annotated features in POS and NEG ion modes were used for downstream analyses. All raw data with annotations were described in Dataset EV5. The low repeatability annotated features were excluded, whose relative standard deviation (RSD) was ≥25% in the pooled QC samples. Prior to statistical analysis, data were median-normalized and log10-transformed using *MetaboAnalystR* package version 4.0.0 in R version 4.2.3 (Chong and Xia, 2018). A linear mixed-effects model was conducted, incorporating group, sex, and birth weight as fixed factors and litter as a random factor, using the *lme4* package (Bates et al, 2015). An FDR cut-off of 0.1 was applied to derive molecules with differential abundance (MDAs). MDA-based pathway analysis, which integrated a hypergeometric test and out-degree centrality, was performed in the MetaboAnalyst 6.0 (https://www.metaboanalyst.ca) with *Sus scrofa* KEGG knowledgebase. Pathways with a *P* value cut-off of 0.05 and more than one significant hit were considered statistically significant.

#### Statistics

All statistical analyses of non-omics data were executed via R version 4.3.2 unless otherwise stated. For discerning differences at specific intervals (3, 6, 12, and 15 h), continuous data underwent analysis via a linear mixed-effects model, incorporating group, sex, and birth weight as fixed factors and litter as a random factor, using the *lme4* package (Bates et al, 2015). Another linear mixed-effects model was employed to probe further disparities spanning the entire experimental duration. This model integrated group, time, their interaction, sex, and birth weight as fixed factors, with litter and pig ID as random factors, using the *lme4* package (Bates et al, 2015). Normal distribution, variance homogeneity of residuals, and fitted values were assessed. The data that did not conform to a normal distribution were logarithmically transformed. If the transformation did not achieve an approximate log-normal distribution, the non-parametric Mann–Whitney *U* test was used instead. To examine different strategies in the infection response, reaction norm analysis was performed using blood pH as a readout for health at 3, 6, and 15 h and plotted against the pathogen burdens at the same time point by linear regression. An extra sum-of-squares F Test was used to compare slopes. Statistical significance was defined as *P* value < 0.05.

#### Study approval

The Danish Animal Experiments Inspectorate approved all animal studies and experimental procedures under license number 2020-15-0201-00520. These approvals are in accordance with EU Directive 2010/63, which governs the legislation for the use of animals in research. The study report was adhered to ARRIVE guidelines.

### Graphics

Figures 1A, 2D, 3D, 5 and 6A were created with Biorender.com.

## Supplementary information

Table EV1

Table EV2

Appendix

Peer Review File

Dataset EV1

Dataset EV2

Dataset EV3

Dataset EV4

Dataset EV5

Source data Fig. 1

Source data Fig. 2

Source data Fig. 3

Source data Fig. 4

Source data Fig. 5

Source data Fig. 6

Source data Fig. 7

Expanded View Figures

## Data Availability

The raw and processed RNA-seq data generated from this study were deposited in the NIH Gene Expression Omnibus, with accession number GSE263512. The original and normalized hepatic and plasma metabolomics data were attached to the supplementary data. The pipeline for the analysis is available on GitHub (https://github.com/Pharmaco-OmicsLab/NUTRISEPSIS). The source data of this paper are collected in the following database record: biostudies:S-SCDT-10_1038-S44321-026-00463-z.
