# Supplementary material for: Harnessing the glycolysis-TCA cycle axis to boost host defense against neonatal infection
Source: EMBO Mol Med. 2026 Jun 8;18(7):2896–919. doi: 10.1038/s44321-026-00463-z (PMC13365532; doi:10.1038/s44321-026-00463-z)
Supplement: Supplementary file 2 — Table EV2 [file 44321_2026_463_MOESM2_ESM.docx]

**Table EV2: Infection rate ratios for glycolysis metabolites**

|  |  | From Birth to 36 months | | From 18 to 36 months | |
| --- | --- | --- | --- | --- | --- |
| Metabolite | Infection | IRR (95% CI) | P | IRR (95% CI) | P |
| Pyruvate | All Infections | 0.96 (0.78-1.17) | 0.676 | 0.86 (0.71-1.05) | 0.139 |
|  | Pneumonia | 1.29 (0.47-3.50) | 0.622 | 0.83 (0.28-2.50) | 0.738 |
|  | Acute otitis | **0.47 (0.25-0.88)** | **0.019** | 0.56 (0.25-1.27) | 0.167 |
|  | Tonsilitis | 0.40 (0.09-1.77) | 0.228 | 1.50 (0.42-5.41) | 0.532 |
|  | Fever | 1.16 (0.83-1.62) | 0.376 | 0.86 (0.59-1.25) | 0.424 |
|  | Cold | 0.86 (0.68-1.09) | 0.212 | 0.83 (0.64-1.08) | 0.170 |
|  | Gastroenteritis | 1.38 (0.76-2.51) | 0.295 | 1.35 (0.70-2.62) | 0.373 |

Association between plasma relative abundance levels of pyruvate and early life infections. Infection risk estimated by quasi-poission regression. Both shown from birth until 36 months of age and from 18 to 36 months of age. CI: confidence interval, IRR: Infection Rate Ratio. N = 678 for data at birth, and n = 538 for data at 18 months.
