# Supplementary material for: Harnessing the glycolysis-TCA cycle axis to boost host defense against neonatal infection
Source: EMBO Mol Med. 2026 Jun 8;18(7):2896–919. doi: 10.1038/s44321-026-00463-z (PMC13365532; doi:10.1038/s44321-026-00463-z)
Supplement: Supplementary file 3 — Appendix [file 44321_2026_463_MOESM3_ESM.pdf]

Table of Contents

Appendix Figure S1 2
Appendix Figure S2 4
Appendix Figure S3 5
Appendix Figure S4 7
Appendix Figure S5 9
Appendix Figure S6 10
Appendix Figure S7 11
Appendix Figure S8 12

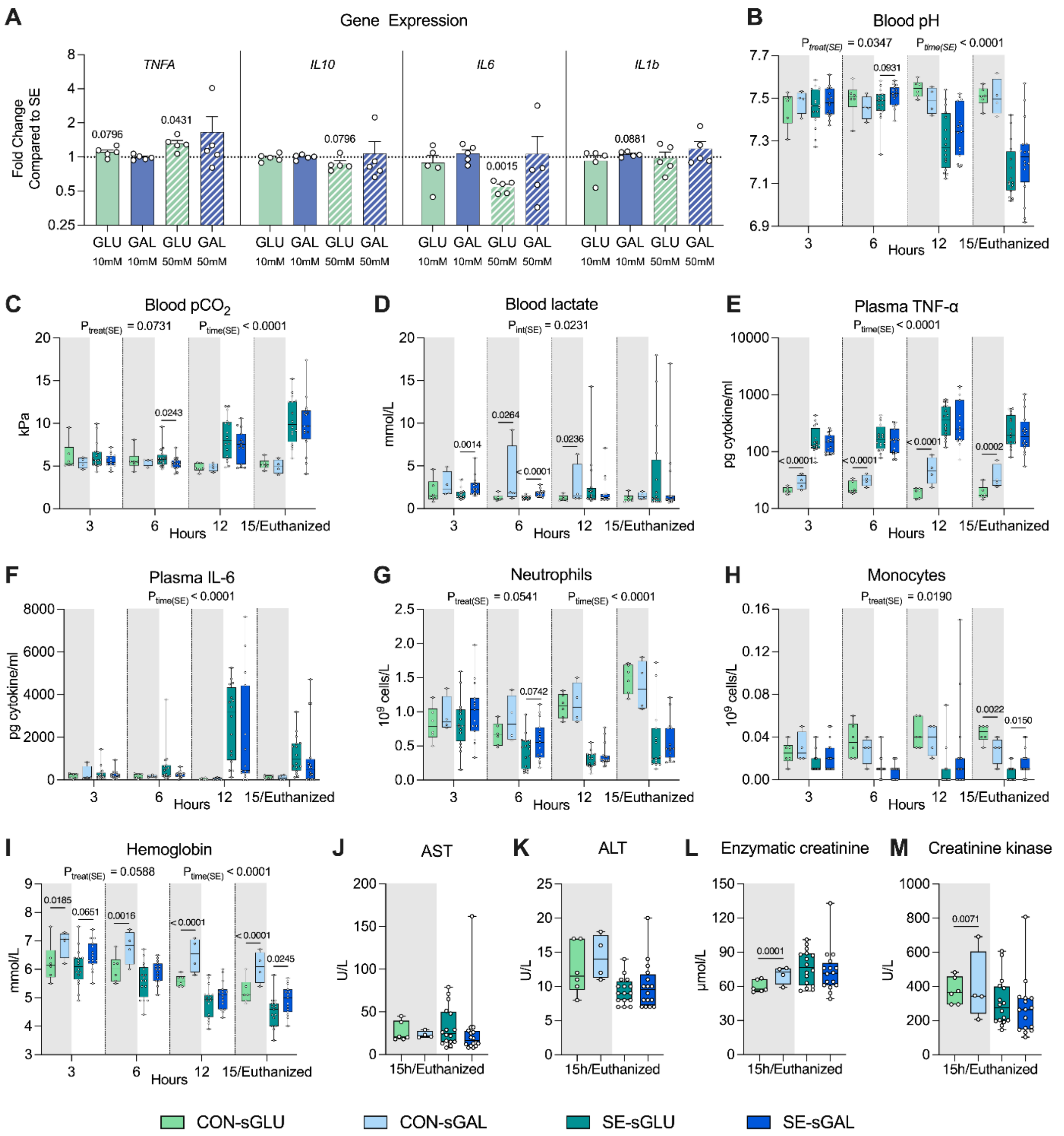

Appendix Figure S1. Effects of galactose supply on blood gas and immune parameters during neonatal infection in preterm piglets. (A) Four cytokine gene expressions are shown as relative fold changes in relation to *S. epidermidis* positive control. Normality was assessed before statistical testing. For normally distributed data, paired t-tests were used; otherwise, Wilcoxon matched-pairs signed-rank tests were used. Data are shown as bar plots with individual data points. Bars indicate the mean, and error bars indicate SD. P-values indicate comparisons with the corresponding infected control without added glucose or galactose. **(B-I)** Blood and immune parameters (blood pH, pCO_2, lactate, total leukocytes, neutrophils, monocytes, hemoglobin, plasma TNF-α, and IL-6) were collected at 3, 6, 12, and 15 h post-inoculation or at euthanasia. **(J–M)** Serum biochemistry parameters (aspartate transaminase, alanine transaminase, enzymatic creatinine, and creatinine

kinase) at 15 h post-inoculation or at euthanasia. **Statistics**: **(B-M)** Data at each time point were analyzed separately via a linear mixed-effects model, incorporating group, gender, and birth weight as fixed factors and litter as a random factor. **(B-I)** Another linear mixed-effects model was employed to probe further disparities spanning the entire experimental duration, incorporating group, time, their interaction, gender, and birth weight as fixed factors, with litter and pig ID as random factors. $P_{treat(SE)}$, $P_{time(SE)}$, and $P_{int(SE)}$ denote probability values for group effect (SE-sGAL and SE-sGLU) over time, time effects, and the interaction effects between time and group in the linear mixed effects interaction model, respectively. Uninfected animals (CON) served as the reference group and were not directly compared with infected animals (SE). Data are shown as box-and-whisker plots. The centre line indicates the median; the lower and upper bounds of the box indicate the 25th and 75th percentiles, respectively; and the whiskers extend from the minimum to the maximum values. All individual data points are shown. In (A), n=5/group. In (B-I), n=15-16/group for infected groups and n=4-6/group for uninfected groups.

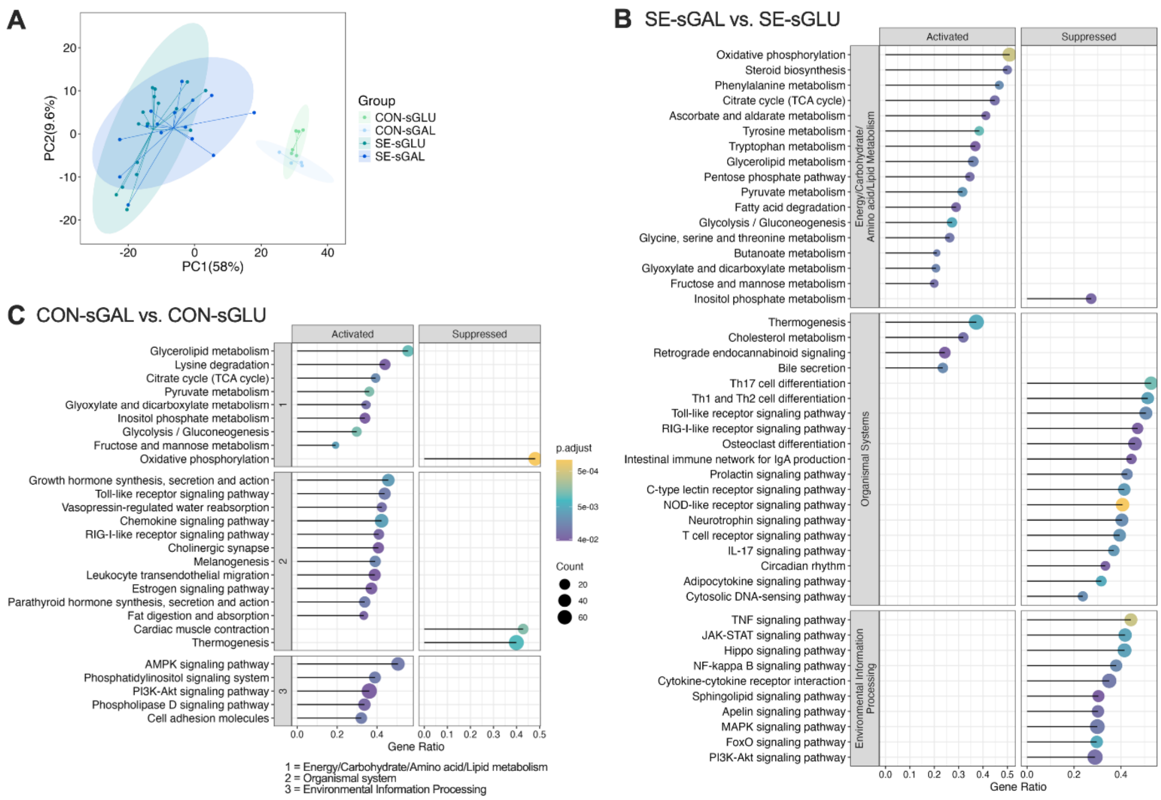

Appendix Figure S2. Galactose supply reduces inflammation and enhances multiple hepatic metabolic pathways in infected preterm piglets. (A) PCA scores plot of the first two principal components. **(B,C)** GSEA was performed between the SE-sGAL vs SE-sGLU as well as infected vs uninfected groups, respectively. The *Sus scrofa* (pig) KEGG knowledgebase was utilized for pathway enrichment analysis. Significant pathways in specific categories such as energy metabolism, carbohydrate metabolism, amino acid metabolism, lipid metabolism, signal transduction, and immune system have been chosen for presentation. The complete list of enriched pathways can be found in **Dataset EV1A**. The size and color of the dots indicate the gene ratio and FDR values, respectively. n=15-16/group for infected groups and n=4-6/group for uninfected groups.

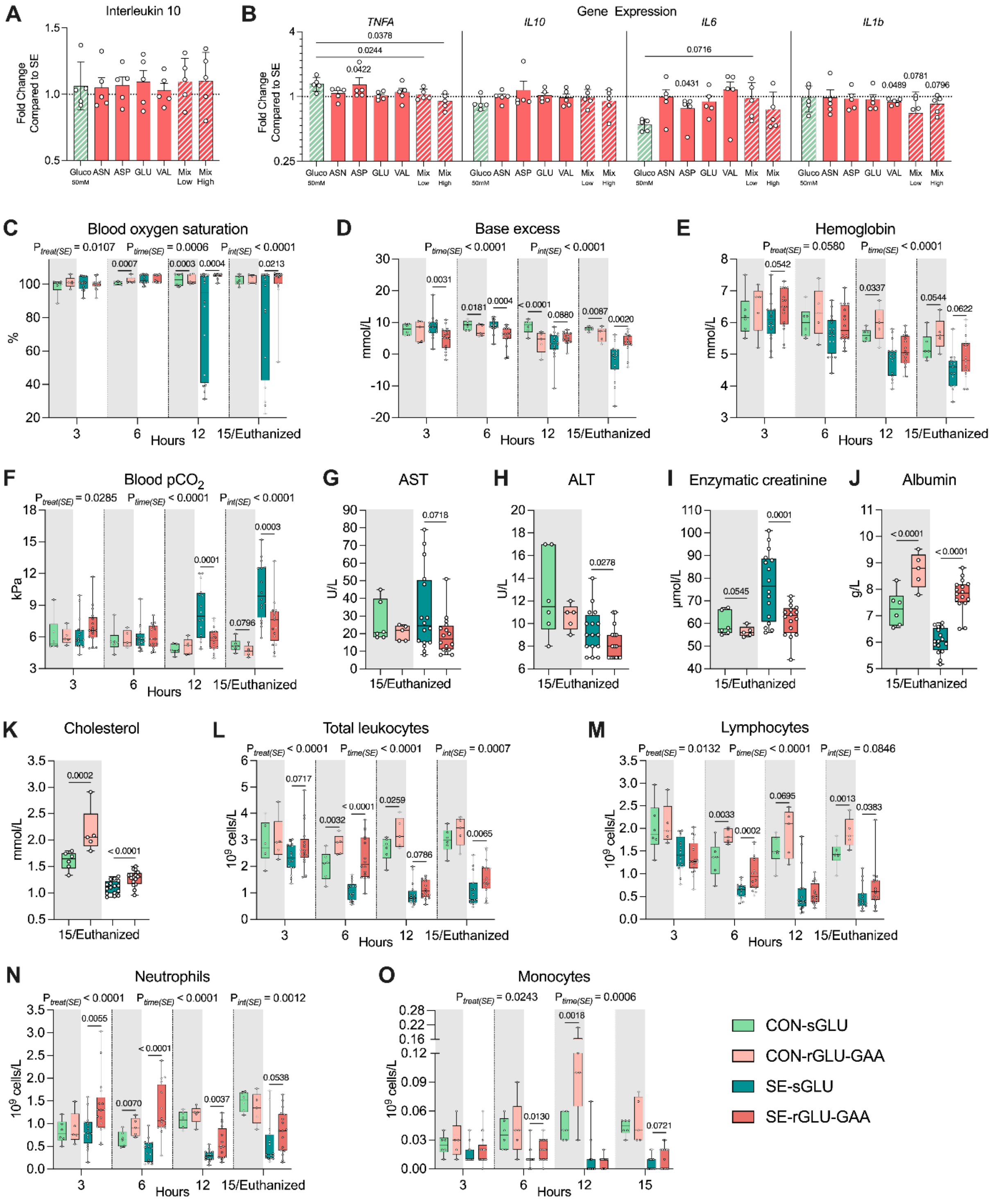

Appendix Figure S3. Combined glucose restriction and glucogenic amino acid supply enhances both host disease resistance and tolerance in infected preterm piglets. (A) *In vitro* human macrophage-like THP1 cells exposed to live *S. epidermidis*. Levels of interleukin 10 are

shown as relative fold changes in relation to *S. epidermidis* positive control. **(B)** Four cytokine gene expressions are shown as relative fold changes in relation to *S. epidermidis* positive control. **(A–B)** Normality was assessed before statistical testing. For normally distributed data, paired t-tests were used; otherwise, Wilcoxon matched-pairs signed-rank tests were used. Data are shown as bar plots with individual data points. Bars indicate the mean, and error bars indicate SD. P-values indicate comparisons with the corresponding infected control without added amino acids. **(C–F)** Blood pCO_2, oxygen saturation, base excess, and hemoglobin at 3, 6, 12, and 15 h post-bacterial inoculation or at euthanasia. **(G–K)** Serum aspartate transaminase (AST), alanine transaminase (ALT), enzymatic creatine, and albumin at 15 h post-inoculation or at euthanasia. **(L–O)** Blood immune parameters (blood total leukocytes, lymphocytes, neutrophils, and monocytes) at 3, 6, 12, and 15 h post-bacterial inoculation or at euthanasia. **Statistics: (C–O)** Data at each time point were analyzed using a linear mixed-effects model, incorporating group, gender, and birth weight as fixed factors and litter as a random factor. **(C–F & L–O)** Another linear mixed-effects model was employed to probe further disparities spanning the entire experimental duration, incorporating group, time, their interaction, gender, and birth weight as fixed factors, with litter and pig ID as random factors. $P_{treat(SE)}$, $P_{time(SE)}$, and $P_{int(SE)}$ denote probability values for group effect (SE-rGLU-GAAs and SE-sGLU) over time, time effects, and the interaction effects between time and group in the linear mixed effects interaction model, respectively. Uninfected animals (CON) served as the reference group and were not directly compared with infected animals (SE). Data are shown as box-and-whisker plots. The centre line indicates the median; the lower and upper bounds of the box indicate the 25th and 75th percentiles, respectively; and the whiskers extend from the minimum to the maximum values. All individual data points are shown. In (A-B), n=5/group. In (C-O), n=15-17/group for infected groups and n=5-6/group for uninfected groups.

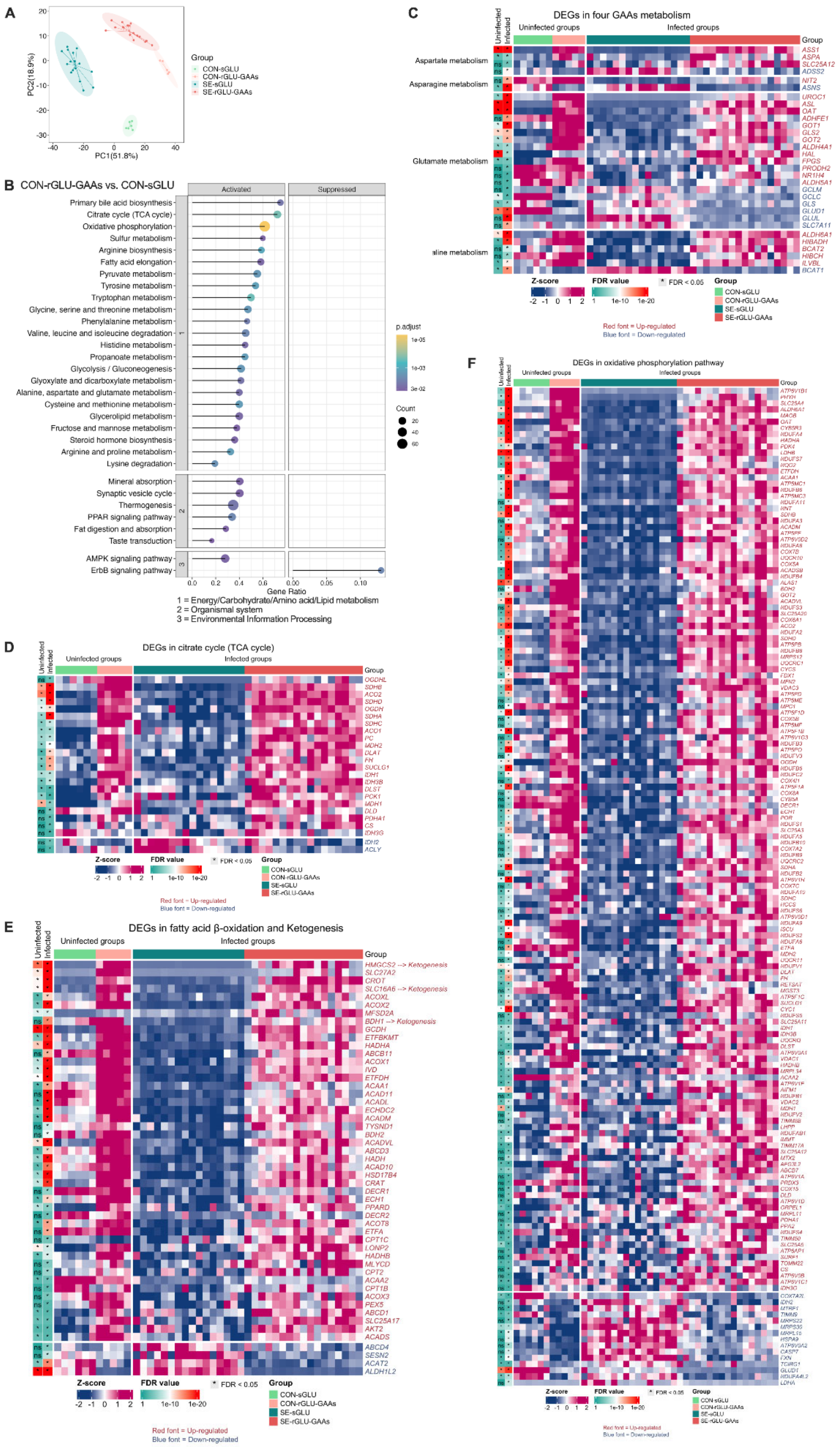

Appendix Figure S4. Effects of rGLU-GAAs supply on hepatic metabolism in preterm piglets. (A) PCA of hepatic transcriptome between all groups **(B)** GSEA was performed between the CON-rGLU-GAAs and CON-sGLU groups using the *Sus scrofa* (pig) KEGG knowledgebase.

Significant pathways in specific categories such as energy metabolism, carbohydrate metabolism, amino acid metabolism, lipid metabolism, signal transduction, and immune system have been chosen for presentation. The complete list of enriched pathways can be found in **Dataset EV2B**. The size and color of the dots indicate the gene ratio and FDR values, respectively. **(C–F)** Heatmaps illustrating DEGs involved in the specific metabolic pathways between SE-rGLU-GAAs and SE-sGLU groups. n=16-17/group for infected groups and n=5-6/group for uninfected groups.

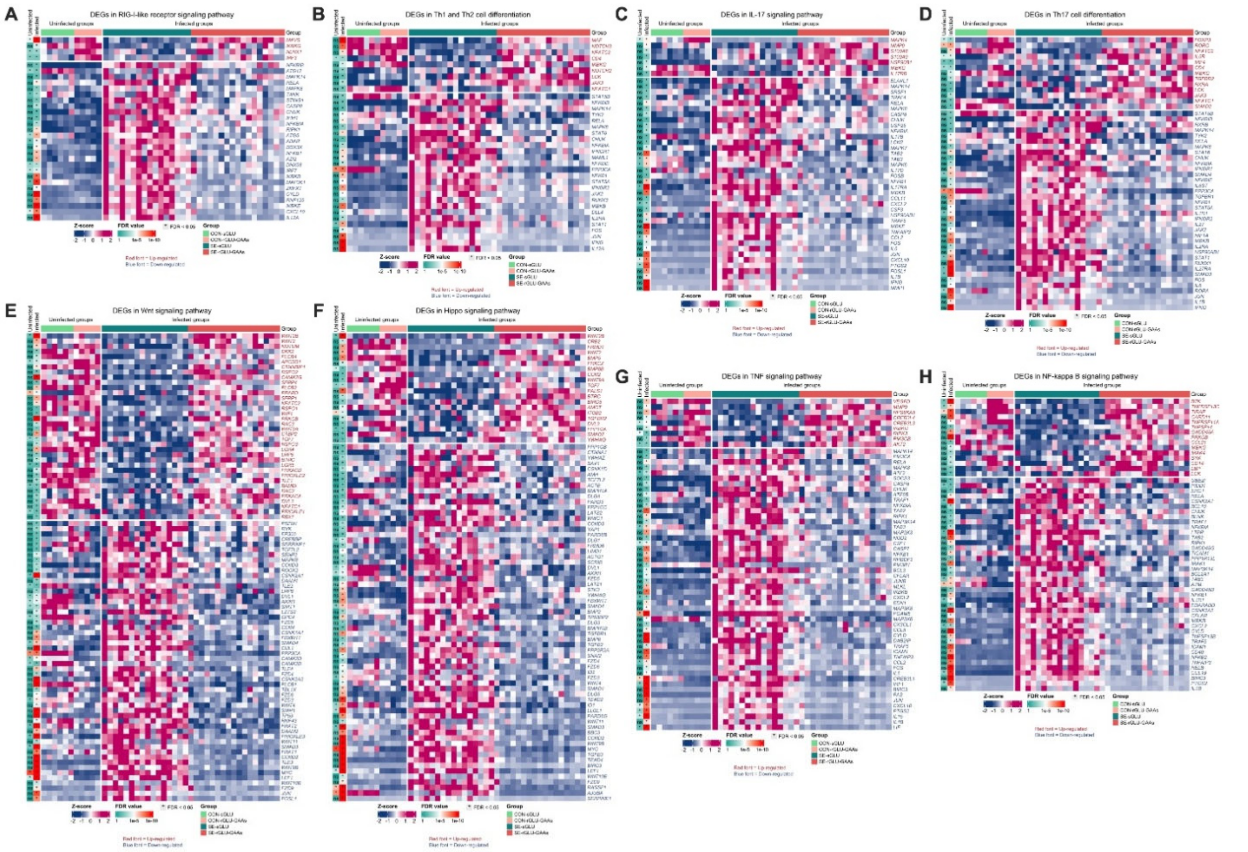

Appendix Figure S5. rGLU-GAAs supply suppressed hepatic pathways related to immunity and signal transduction in infected preterm piglets. (A-H) Heatmaps illustrating DEGs involved in the critical immune/inflammation-related pathways between SE-rGLU-GAAs and SE-sGLU groups. n=16-17/group for infected groups and n=5-6/group for uninfected groups.

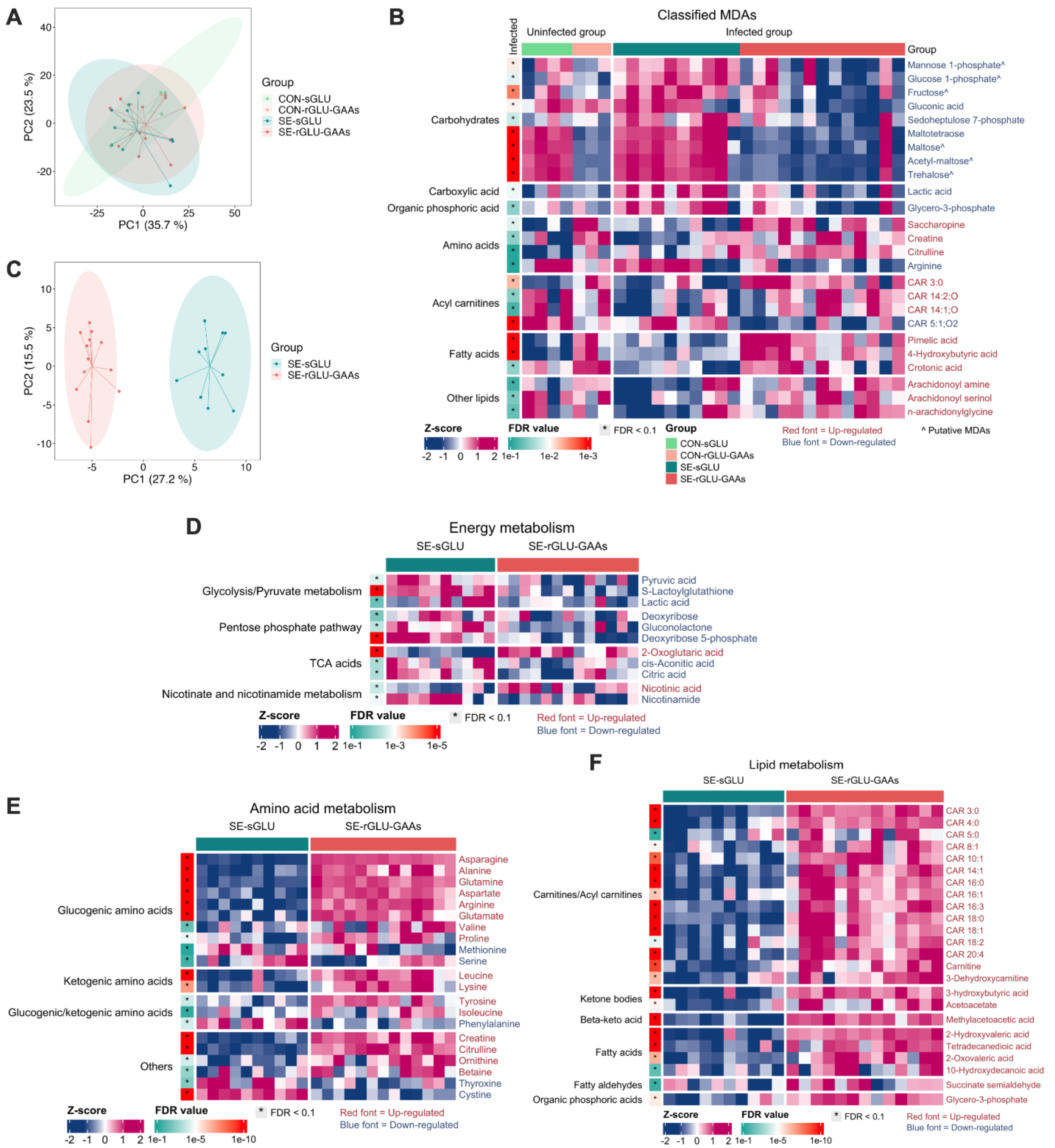

Appendix Figure S6. rGLU-GAAs supply during infection affect plasma and hepatic metabolome in preterm piglets. (A-B) PCA analysis and heatmap showing metabolites with differential abundances from hepatic metabolome. (**C-E**) PCA analysis and heatmaps showing plasma metabolites with differential abundances in different metabolic categories. n=10-13/group for infected groups and n=3-4/group for uninfected groups.

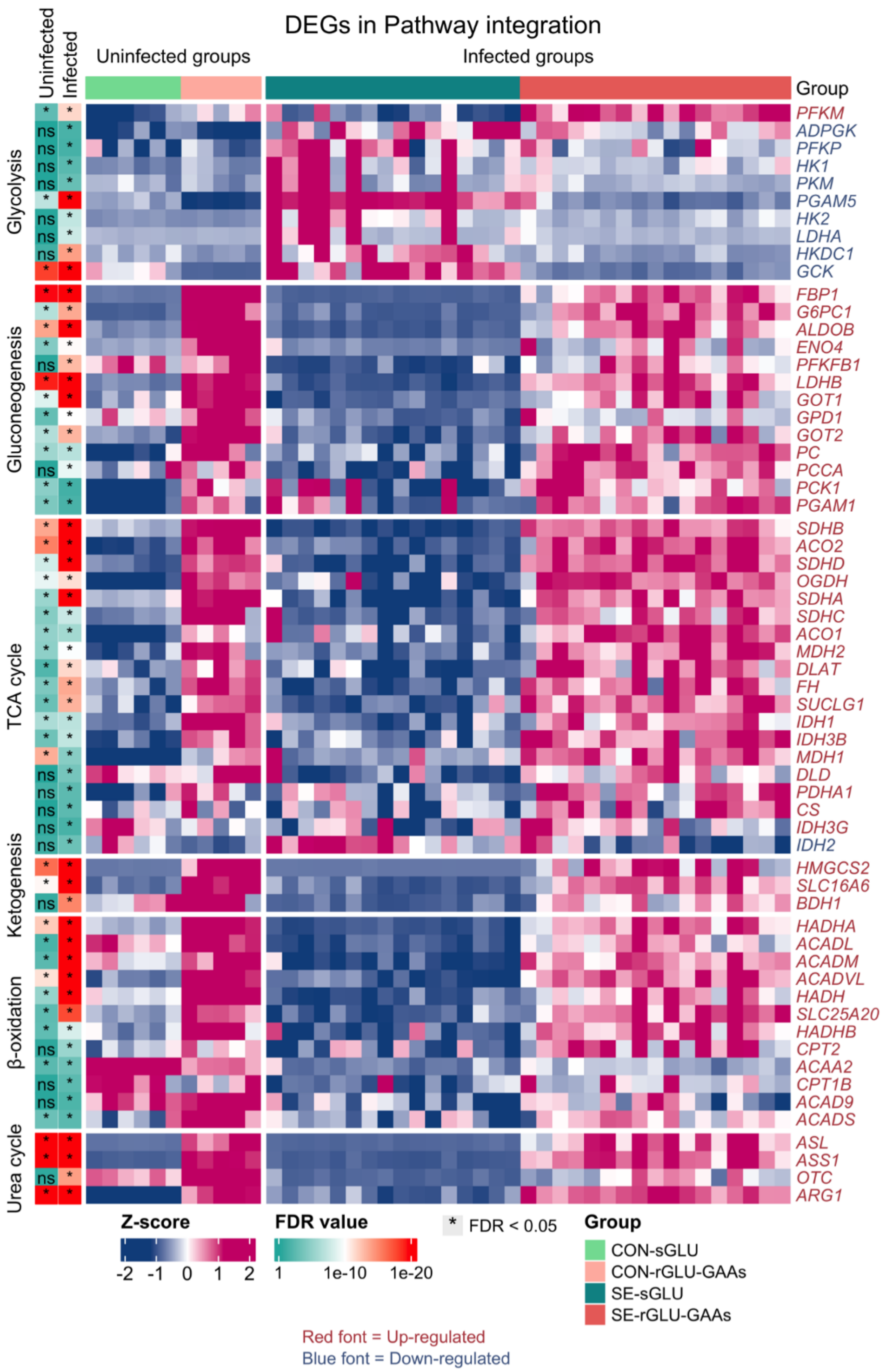

Appendix Figure S7. Effects of rGLU-GAAs supply on hepatic metabolism in infected preterm piglets. (A-H) Heatmap illustrating DEGs between SE-rGLU-GAAs and SE-sGLU groups involved in **Figure 5**. n=16-17/group for infected groups and n=5-6/group for uninfected groups.

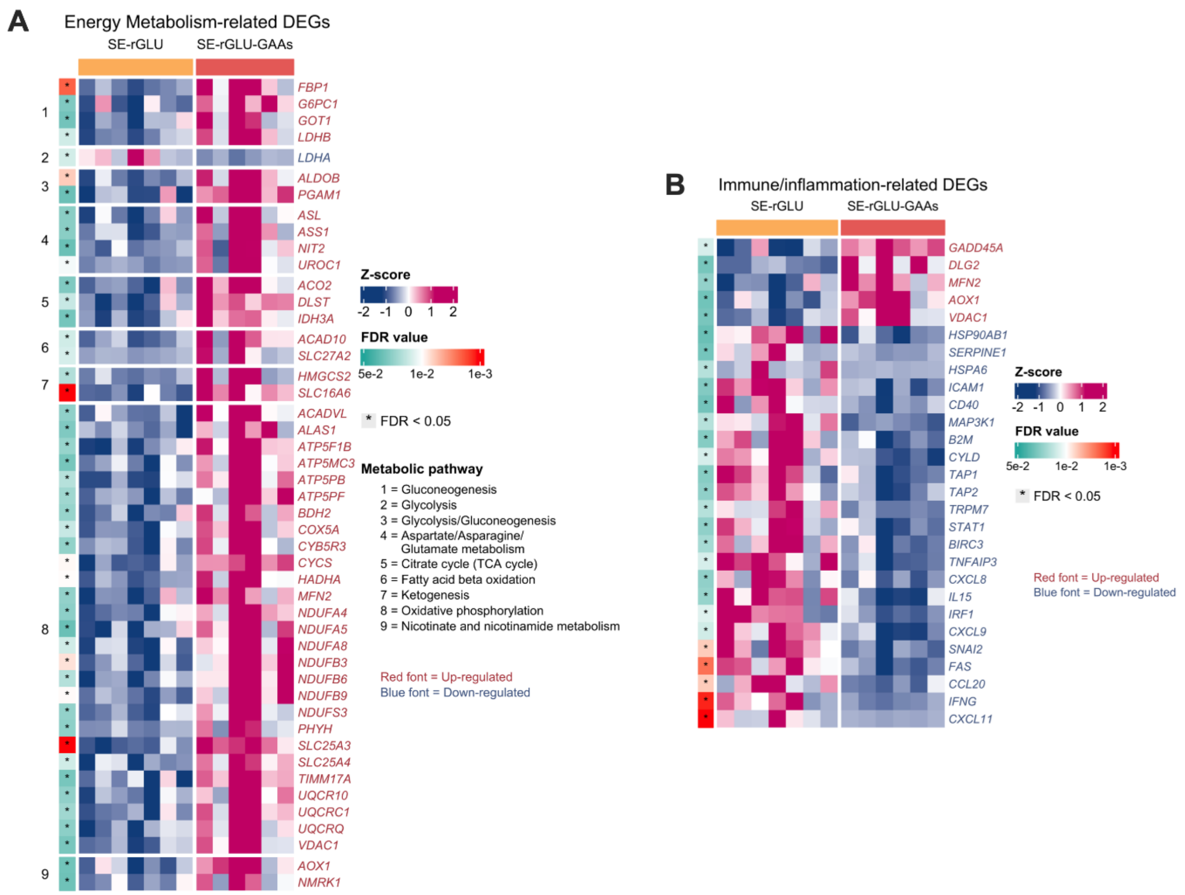

Appendix Figure S8. Effects of GAA supply during glucose restriction on hepatic gene expressions related to energy metabolism (A) and inflammation (B) in infected preterm piglets. n=6-7/group.
