# Supplementary material for: Harnessing the glycolysis-TCA cycle axis to boost host defense against neonatal infection
Source: EMBO Mol Med. 2026 Jun 8;18(7):2896–919. doi: 10.1038/s44321-026-00463-z (PMC13365532; doi:10.1038/s44321-026-00463-z)
Supplement: Supplementary file 17 — Expanded View Figures [file 44321_2026_463_MOESM17_ESM.pdf]

Expanded View Figures

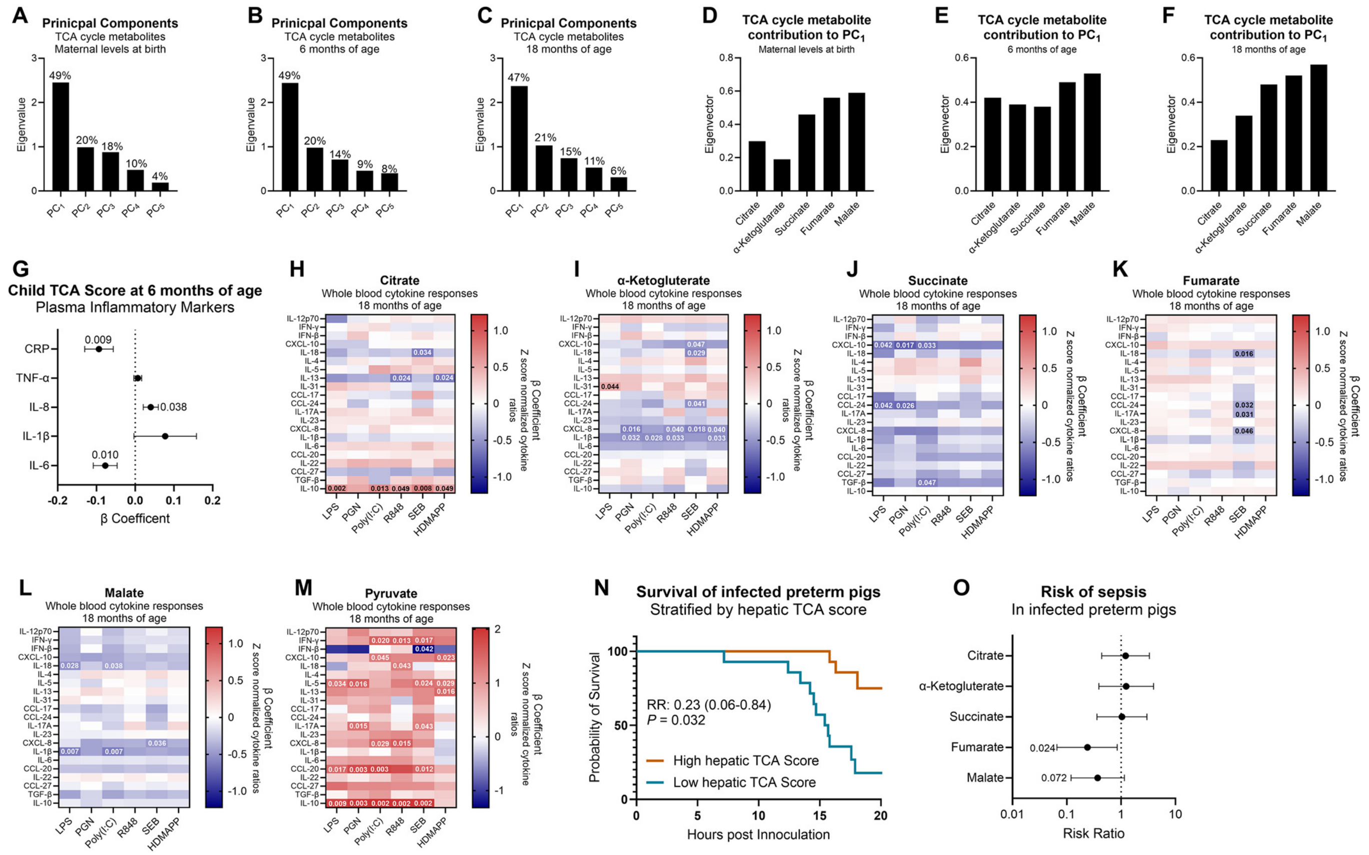

Figure EV1. Association of TCA metabolites with inflammation in newborn infants and infected preterm piglets.

(**A–C**) Eigenvalues showing the fraction each principal component (PC) contributes to the overall PCA at birth, 6, and 18 months of age. (**D–F**) Eigenvectors showing how each TCA metabolite contributes to PC_1 (TCA scores) at birth, 6, and 18 months of age. (**G**) Associations between 6-month TCA scores and plasma inflammatory markers with estimated coefficient and 95% CIs. CRP, C-reactive protein; TNF, tumor necrosis factor; IL, Interleukin, analyzed by generalized linear model and shown as β-coefficients with 95% CIs. (**H–M**) Heatmaps showing the association between individual TCA cycle metabolites and pyruvate at 18 months of age and z-score normalized cytokine ratios following whole blood stimulation with 6 pattern recognition receptor agonists. Shown as β-coefficients following a generalized linear model, where red color indicates positive, and blue negative association. LPS lipopolysaccharide, PGN Peptidoglycan, R848 imidazoquinoline, SEB staphylococcal enterotoxin B, HDMAPP 1-Hydroxy-2-methyl-2-buten-4-yl 4-diphosphate. Data from (**A–M**) were from the COPSAC infant cohort. (**N**) Survival curves for preterm pigs infected with *S. epidermidis*, stratified by hepatic TCA score (high vs. low, divided at the median). Log-rank risk ratios (RR) with corresponding 95% confidence intervals are shown. (**O**) Estimated risk ratios with 95% CIs for risk of sepsis/mortality in infected preterm pigs based on the absolute hepatic levels of each individual TCA-cycle metabolite. Exact *P* values were indicated in each figure panel. For data in (**A–M**), $n = 678$ for maternal data, $n = 562$ and 538 for data at 6 and 18 months, respectively, $n = 28$ in (**N**, **O**).

 © The Author(s)

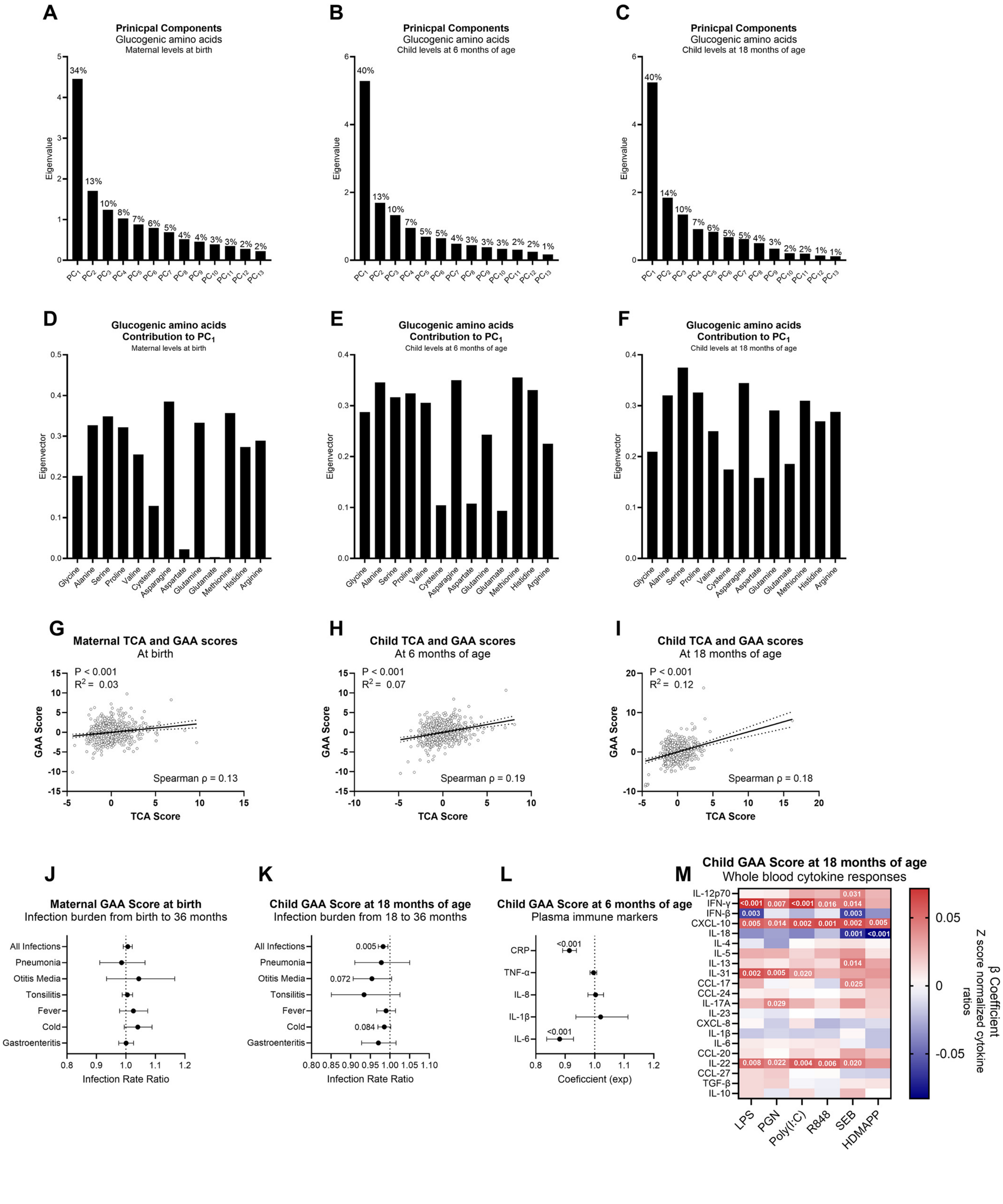
A
Prinicpal Components
Glucogenic amino acids
Maternal levels at birth
Eigenvalue
34%
13%
10%
8%
7%
6%
5%
4%
4%
3%
3%
2%
2%
PC1 PC2 PC3 PC4 PC5 PC6 PC7 PC8 PC9 PC10 PC11 PC12 PC13
B
Prinicpal Components
Glucogenic amino acids
Child levels at 6 months of age
Eigenvalue
40%
13%
10%
7%
5%
5%
4%
3%
3%
3%
2%
2%
1%
C
Prinicpal Components
Glucogenic amino acids
Child levels at 18 months of age
Eigenvalue
40%
14%
10%
7%
6%
5%
5%
4%
3%
2%
2%
1%
1%
D
Glucogenic amino acids
Contribution to PC1
Maternal levels at birth
Eigenvector
Glycine Alanine Serine Proline Valine Cysteine Asparagine Aspartate Glutamine Glutamate Methionine Histidine Arginine
E
Glucogenic amino acids
Contribution to PC1
Child levels at 6 months of age
Eigenvector
F
Glucogenic amino acids
Contribution to PC1
Child levels at 18 months of age
Eigenvector
G
Maternal TCA and GAA scores
At birth
P < 0.001
R2 = 0.03
Spearman ρ = 0.13
GAA Score
TCA Score
H
Child TCA and GAA scores
At 6 months of age
P < 0.001
R2 = 0.07
Spearman ρ = 0.19
GAA Score
TCA Score
I
Child TCA and GAA scores
At 18 months of age
P < 0.001
R2 = 0.12
Spearman ρ = 0.18
GAA Score
TCA Score
J
Maternal GAA Score at birth
Infection burden from birth to 36 months
All Infections
Pneumonia
Otitis Media
Tonsilitis
Fever
Cold
Gastroenteritis
Infection Rate Ratio
K
Child GAA Score at 18 months of age
Infection burden from 18 to 36 months
All Infections
Pneumonia
Otitis Media
Tonsilitis
Fever
Cold
Gastroenteritis
0.005
0.072
0.084
Infection Rate Ratio
L
Child GAA Score at 6 months of age
Plasma immune markers
CRP
TNF-α
IL-8
IL-1β
IL-6
<0.001
<0.001
Coeficcient (exp)
M
Child GAA Score at 18 months of age
Whole blood cytokine responses
IL-12p70
IFN-γ
IFN-β
CXCL-10
IL-18
IL-4
IL-5
IL-13
IL-31
CCL-17
CCL-24
IL-17A
IL-23
CXCL-8
IL-1β
IL-6
CCL-20
IL-22
CCL-27
TGF-β
IL-10
LPS PGN Poly(I:C) R848 SEB HDMAPP
β Coefficient
Z score normalized cytokine ratios

Figure EV2. Correlation of TCA and GAA scores and association of GAA score and infection burdens and inflammation in infants.

(**A–C**) Eigenvalues showing the fraction each principal component (PC) contributes to the overall PCA at birth, 6, and 18 months of age for GAAs. (**D–F**) Eigenvectors showing how each TCA metabolite contributes to PC_1 (TCA scores) at birth, 6, and 18 months of age. (**G–I**) Spearman correlation between TCA and GAA scores at birth, 6 and 18 months of age. (**J**, **K**) Association of GAA scores at birth and 18 months with infection burdens from birth to 36 months and 18-36 months, respectively. Both are shown as estimated risk ratios with 95% CIs, derived from quasi-Poisson regression. (**L**) Associations between 6-month GAA scores and plasma inflammatory markers. CRP: C-reactive protein, TNF: Tumor necrosis factor, IL: Interleukin, analyzed by generalized linear model shown as estimated β-coefficients with 95% CIs. (**M**) Heatmaps showing the association between individual GAA scores at 18 months of age and z-score normalized cytokine ratios following whole blood stimulation with 6 pattern recognition receptor agonists. Shown as β-coefficients following a generalized linear model where red color indicates positive, and blue negative association. LPS lipopolysaccharide, PGN peptidoglycan, R848 imidazoquinoline, SEB staphylococcal enterotoxin B, HDMAPP 1-Hydroxy-2-methyl-2-buten-4-yl 4-diphosphate. Exact *P* values were indicated in each figure panel. $n = 678$ for maternal data, $n = 562$ and 538 for data at 6 and 18 months, respectively.

 © The Author(s)
