# Supplementary material for: Harnessing the glycolysis-TCA cycle axis to boost host defense against neonatal infection
Source: EMBO Mol Med. 2026 Jun 8;18(7):2896–919. doi: 10.1038/s44321-026-00463-z (PMC13365532; doi:10.1038/s44321-026-00463-z)
Supplement: Supplementary file 4 — Peer Review File [file 44321_2026_463_MOESM4_ESM.pdf]

EMBO Molecular Medicine

Harnessing the glycolysis-TCA cycle axis to boost host defense against neonatal infection

Ziyuan Wu, Nguyen Tran Nam Tien, Bjorn Klabunde, Karoline Aasmul-Olsen, Simone Offersen, Nguyen Thi Hai Yen, Tik Muk, Anna Thysen, Susanne Brix, Nicklas Brustad, Tingting Wang, Jakob Stokholm, Klaus Bønnelykke, Anders Brunse, Nguyen Phuoc Long, Bo Chawes, Ole Bæk, and Duc Ninh Nguyen

Corresponding author(s): Duc Ninh Nguyen (dnn@sund.ku.dk) , Ole Bæk (ole.baek@sund.ku.dk)

Review Timeline:

Submission Date:	12th Jan 26
Editorial Decision:	4th Mar 26
Revision Received:	14th Apr 26
Editorial Decision:	6th May 26
Revision Received:	20th May 26
Accepted:	26th May 26

Editor: Lise Roth

Transaction Report:

(Note: With the exception of the correction of typographical or spelling errors that could be a source of ambiguity, letters and reports are not edited. Depending on transfer agreements, referee reports obtained elsewhere may or may not be included in this compilation. Referee reports are anonymous unless the Referee chooses to sign their reports.)

4th Mar 2026

Dear Dr. Nguyen,

Thank you for submitting your manuscript to our editorial office. Please accept my apologies for the delay in responding, which was due to the time it took to find suitable referees and the fact that one of them required extra time to complete their review. We have now received feedback from the three reviewers who agreed to evaluate your study. As you will see from the reports below, while they acknowledge the potential interest of the findings, they nevertheless raise major concerns relating to the model system and the distinction between association and causation.

If you feel you can satisfactorily address these points and those listed by the referees, you may wish to submit a revised version of your manuscript. Please attach a covering letter giving details of the way in which you have handled each of the concerns raised by the referees. A revised manuscript will once again be subject to review, and we cannot guarantee at this stage that the eventual outcome will be favorable.

If you would like to discuss further the reports from the referees, I am available to do so via email or video. Let me know if you are interested in this option.

We are expecting your revised manuscript within three months, if you anticipate any delay, please contact us.

When preparing your revised manuscript, please refer to our guidelines: https://link.springer.com/journal/44321/submission-guidelines#cms-Revised-submissions. We perform an initial quality control of all revised manuscripts before re-review; failure to include requested items will delay the evaluation of your revision.

We require:

1) A .docx formatted version of the manuscript text (including legends for main figures, EV figures and tables). Please make sure that the changes are highlighted to be clearly visible.

2) Individual production quality figure files as .eps, .tif, .jpg (one file per figure). For guidance, download the 'Figure Guide PDF': https://media.springernature.com/original/springer-cms/rest/v1/content/27825798/data/v1.

3) A .docx formatted letter INCLUDING the reviewers' reports and your detailed point-by-point responses to their comments. As part of the EMBO Press transparent editorial process, the point-by-point response is part of the Review Process File (RPF), which will be published alongside your paper.

4) A complete author checklist, which you can download from our author guidelines. Please insert information in the checklist that is also reflected in the manuscript. The completed author checklist will also be part of the RPF.

5) Please note that all corresponding authors are required to supply an ORCID ID for their name upon submission of a revised manuscript.

6) It is mandatory to include a 'Data Availability' section after the Materials and Methods. Before submitting your revision, primary datasets produced in this study need to be deposited in an appropriate public database, and the accession numbers and database listed under 'Data Availability'. Please remember to provide a reviewer password if the datasets are not yet public.

In case you have no data that requires deposition in a public database, please state so in this section. Note that the Data Availability Section is restricted to new primary data that are part of this study.

7) For data quantification: please specify the name of the statistical test used to generate error bars and P values, the number (n) of independent experiments (specify technical or biological replicates) underlying each data point and the test used to calculate p-values in each figure legend. The figure legends should contain a basic description of n, P and the test applied. Graphs must include a description of the bars and the error bars (s.d., s.e.m.).

8) At EMBO Press we ask authors to provide source data for the main manuscript figures. You will receive a separate email with instructions for providing source data with your revised manuscript, including how to upload and organize the files.

9) Our journal encourages inclusion of *data citations in the reference list* to directly cite datasets that were re-used and obtained from public databases. Data citations in the article text are distinct from normal bibliographical citations and should directly link to the database records from which the data can be accessed. In the main text, data citations are formatted as follows: "Data ref: Smith et al, 2001" or "Data ref: NCBI Sequence Read Archive PRJNA342805, 2017". In the Reference list, data citations must be labeled with "[DATASET]". A data reference must provide the database name, accession

number/identifiers and a resolvable link to the landing page from which the data can be accessed at the end of the reference.

10) We replaced Supplementary Information with Expanded View (EV) Figures and Tables that are collapsible/expandable online. A maximum of 5 EV Figures can be typeset. EV Figures should be cited as 'Figure EV1, Figure EV2" etc... in the text and their respective legends should be included in the main text after the legends of regular figures.

- For the figures that you do NOT wish to display as Expanded View figures, they should be bundled together with their legends in a single PDF file called *Appendix*, which should start with a short Table of Content. Appendix figures should be referred to in the main text as: "Appendix Figure S1, Appendix Figure S2" etc.

- Additional Tables/Datasets should be labeled and referred to as Table EV1, Dataset EV1, etc. Legends have to be provided in a separate tab in case of .xls files. Alternatively, the legend can be supplied as a separate text file (README) and zipped together with the Table/Dataset file.

11) The paper explained: EMBO Molecular Medicine articles are accompanied by a summary of the articles to emphasize the major findings in the paper and their medical implications for the non-specialist reader. Please provide a draft summary of your article highlighting
- the medical issue you are addressing,
- the results obtained and
- their clinical impact.

This may be edited to ensure that readers understand the significance and context of the research. Please refer to any of our published articles for an example.

12) Author contributions: You will be asked to provide CRediT (Contributor Role Taxonomy) terms in the submission system. These replace a narrative author contribution section in the manuscript.

13) A "Disclosure and Competing Interests Statement" should be provided in the main text.

14) Every published paper includes a 'Synopsis' to further enhance discoverability. Synopses are displayed on the journal webpage and are freely accessible to all readers. They include a short stand first (maximum of 300 characters, including space) as well as 2-5 one-sentences bullet points that summarizes the paper. Please write the bullet points to summarize the key NEW findings. They should be designed to be complementary to the abstract - i.e. not repeat the same text. We encourage inclusion of key acronyms and quantitative information (maximum of 30 words / bullet point). Please use the passive voice. Please attach these in a separate file or send them by email, we will incorporate them accordingly.

Please also suggest a striking image or visual abstract to illustrate your article as a PNG file 550 px wide x 300-600 px high.

15) Include a Reagents and Tools Table as part of the Methods section, which can be downloaded from our author guidelines.

16) As part of the EMBO Publications transparent editorial process initiative (see our Editorial at http://embomolmed.embopress.org/content/2/9/329), EMBO Molecular Medicine will publish online a Review Process File (RPF) to accompany accepted manuscripts.
In the event of acceptance, this file will be published in conjunction with your paper and will include the anonymous referee reports, your point-by-point response and all pertinent correspondence relating to the manuscript. Let us know whether you agree with the publication of the RPF and as here, if you want to remove or not any figures from it prior to publication.
Please note that the Authors checklist will be published at the end of the RPF.

EMBO Molecular Medicine has a "scooping protection" policy, whereby similar findings that are published by others during review or revision are not a criterion for rejection. Should you decide to submit a revised version, I do ask that you get in touch after three months if you have not completed it, to update us on the status.

I look forward to receiving your revised manuscript.

Yours sincerely,

Lise Roth

Lise Roth, PhD
Senior Editor
EMBO Molecular Medicine

***** Reviewer's comments *****

Referee #1 (Comments on Novelty/Model System for Author):

For point 4, I am doubting between adequate and not adequate. The paper wants to enforce a breakthrough in neonatal infection/sepsis, and the authors apply a C-section delivered piglet model and one infection model to build their story. This is the weaker point of the paper, the fact that they base their conclusions on one model. It is a good model I think, but still a model, and just one. The model is also quite aggressive and, based on ethical concerns I presume, very short.

Referee #1 (Remarks for Author):

The paper can make use of a very interesting dataset of 700 newborn children, followed for 3 years in terms of health issues and plasma markers, including metabolites. The "TCA" metabolites, measured and reported here would be easier to interpret is they were also complemented with blood glucose, lactate and preferentially also pyruvate and other gluconeogenic molecules like glycerol and amino acids. If they are available, they should be provided.
Fig1B,C,D should mention, in the figure head, that this is maternal, maternal and child TCA levels resp.
I do not see the added value of Fig 1F in this paper? Is this pattern of response different than whole blood responses of adults or so? Fig 1G is a bit misleading and the title of the figure should mention piglets: it falls under a figure with title that refers to children, while 1G deals with piglets. Though the data are clear and data significant, I would urge the authors to be a bit more reluctant with their statements that "improved survival was driven by higher fumarate and malate levels". I would suggest to talk about associations rather that cause & consequence.
Fig2 starts with asking an interesting and relevant question, namely how to 'boost' TCA activity in patients or animals in a safer way than using glucose. Fig 2A,B and C report data obtained by adding Gal or Glu to THP1 cells with S. epidermidis. The choice of the cells is not optimal and should be repeated with blood cells, like was done in Fig1F for example, because THP1, after all, is an immortalized cell line. Also, the 5 replicates are quite low for such an experiment, and it is unclear what the * refers to. What groups are compared with low conc Gal to come to this *? Why is there no condition where just S. epidermidis is added, maybe with and/or without classical carbohydrate sources used in tissue culture, glutamin? The choices of TNF and IL10 are also weird in relation to Fig1F, where IL1 and IL18 were concluded to be the bad cytokines and IL4 the good.
Fig2F: please use the full 0-100% Y axis, this will be more correct. Also, the colors of the lines are not clearly distinctive. Also, I want to ask if the pig model was ever run further than 15h? I think it is rather risky to draw conclusions based on the situation at one final time point. If this was ethically not an option, did the authors consider the use of maybe a mouse model or another model that could allow later timepoints?
The experiment of Fig2 should also report other readouts, that are perhaps more related to lethal outcome in sepsis. Were body temperature and lactate measured? If so, they have to be reported.
Figure 3 is, without doubt, the selling article of the paper. Though my concerns about THP1 also hold true here, and the Y axis of the survival curve, the data nicely show the protective impact of GNEO amino acids in this particular piglet model (which is still far from a newborn human study of course). The extra experiment in Figure 6 looks redundant to me.
The biggest questions I have after reading the paper are the following: while starting the paper with human neonatal infection/sepsis, the paper quickly goes into a C-section delivered piglet model, never to really return to human neonatal sepsis. In other words, the readouts in Fig1 that suggest that TCA activity is important in surviving/reducing infections (not a big surprise as such) eventually leads to conclusions about the sugar source or even that gluconeogenesis should be stimulated to let the patient produce his/her glucose him/herself, by adding amino acids. The authors should now return to the neonates and their mothers and measure these amino acids and see if their concentration related to their infections. I expect the authors to address this.
Another question relates to the fact that this is a highly experimental model where animals are treated quite instantly with the metabolites. It is known that sepsis leads to fast block of gluconeogenesis in the liver. Therefore, it would be essential to test the AAs therapy when these are applied to the piglets quite some time after onset of sepsis, perhaps also in a model that is less aggressive and does not lead to deaths in 15h.
Finally, the authors should consider to what extent their findings are selective for the E. epidermidis model, so another model would be very useful to validate their findings.

Referee #2 (Comments on Novelty/Model System for Author):

The integration of cohort, in vitro, and animal data is a major strength

Referee #2 (Remarks for Author):

This manuscript examines how the tricarboxylic acid (TCA) cycle activity shapes neonatal defense against infection. Using human birth-cohort metabolomics, in vitro macrophage experiments, and a preterm piglet sepsis model, the authors show that boosting hepatic TCA cycle flux via galactose substitution or glucogenic amino acid supplementation can enhance disease tolerance and resistance during neonatal sepsis. The study identifies metabolic rewiring as a potential therapeutic approach to improve infection outcomes in vulnerable newborns.
Major Comments
The integration of cohort, in vitro, and animal data is a major strength. Some strengths: strong translational design, mechanistic integration across species, innovative nutritional intervention concept, high-quality omics data.
However, the manuscript would benefit from further discussion of TCA activity as a correlative biomarker and/or a causal driver of protection. For instance, discuss potential alternative explanations (e.g., gluconeogenesis vs. TCA flux vs. fatty acid oxidation contributions), and strengthen the mechanistic link between hepatic rewiring and immune modulation.
On the human data, the associations between plasma TCA metabolites and the burden of infection are compelling. However, causality cannot be inferred, and it was recognized in the study's limitations.
For instance, could plasma TCA metabolites reflect general metabolic fitness rather than immune-specific mechanisms? Could the authors discuss the separation between maternal and infant metabolomes?
The preterm pig model is highly valuable. Explicitly define how dosing compares to current neonatal PN practice. Is there a risk involved in the proposed protocol, such as f hypoglycemia, nitrogen load, and acid-base balance?
Provide a schematic summarizing the effects of resistance vs. tolerance across interventions. Is there a risk of immunosuppression with excessive tolerance?
Is there evidence for the lines 141-145? It seems to be overstating.
TCA cycle intermediates were measured in liver tissue. Are there tissue alterations? Did the authors perform histology analysis on the tissue?
Lines 167-169: How can we explain the lower levels of TCA activity? One possibility is mitochondrial dysfunction. Have you looked at it? This reduced activity appears to be a consequence of survival rather than its cause.
Figure 2C: There is an asterisk in the graph. Revise it.
Figure 2E: Did the animals actually die, or is it a prediction?
Figure S2G-I: Check the statistics in Figure S2M. This pattern holds for the 15-hour time point for hemoglobin and monocytes, but not for neutrophils. The CON-sGal group has higher levels of TNF, Hb, creatinine, and creatine kinase (see statistics). Further discuss it.
Line 217-218: How do you explain that? Are the liver injury markers not affected by galactose supplementation?

Figure S4C: groups SE-sGLU had high dispersion; please check the statistics.
Note that con-rGLU-GAAs also increased lactate in 12 and 14 hours (Fig 3G).
Figure S4G, AST was changed.
Figure S4J and K, con-rGLU-GAAs had increased albumin and cholesterol levels. It was not discussed.
Figure S4L-O, different statistics were done, Pin(SE) lymphocytes; there was no statistical difference, but between SE-groups on day 15, there is a difference. However, between-group Pint(SE) did not differ. These are the two key cells; monocytes were much lower, considering removing them.
The figures S6, D, and H were cut.
Line 341, "lower pCO_2," was not different.
Line 343, "GAAs enhanced bacterial clearance at both 3 and 6 h, as well", but not at 15 hours. "as better blood leukocyte replenishment and reduced inflammation at the later phase of the experiment", decreased TNF, but not IL6 (just 12 hours) or altered IL-10.
Check for typos, as on line 403.
Why choose perform metabolomics on plasma over blood immune cells?
Minor Comments
Standardize terminology (OxPhos vs oxidative phosphorylation).
Define all acronyms at first mention.
Check consistency in statistical reporting

Referee #3 (Comments on Novelty/Model System for Author):

This manuscript is possibly paradigm shifting with high translational implications. The authors provide a detailed description of their models which span human to cells. The piglet model is very short lived and doesn't provide information about the long term impact.

Referee #3 (Remarks for Author):

This important work has potentially paradigm shifting translational applications for a considerable problem in the NICU.
The work is comprehensive stemming from cellular data to in vivo and human data.

Some significant concerns arise nonetheless:
-The experiments are very short lived not allowing for a better understanding of the duration of the effect and any longer term implications. Further follow up would be helpful if some animals were allowed to survive and be euthanized at later timepoints.

-No weights over time are provided for the animal data, likely to the short term nature of the experiments and no survival studies. However his needs to be clearly stated in the limitations section.
-The authors didn't measure glycolysis in this model which would be important and easily doable from the existing dataset. Does glycolysis reduce significantly? Glycolysis is important to cell proliferation in many tissues.
-There needs to be transparency around the drop in glucose seen initially. What was this?Is this clinically relevant? I disagree with the authors that humans, especially premature ones, have a full capacity for gluconeogesis. This is the reason we worry about hypoglycemia clinically especially in babies that are growth restricted in utero. Did the cohort distinguish between babies from the groups evaluated?
Is there a clear rationale for the selection of time points for blood sampling in the children? This is important to better understand the intent.
Overall, important insights are provided in this well executed study

Referee #1 (Comments on Novelty/Model System for Author):

For point 4, I am doubting between adequate and not adequate. The paper wants to enforce a breakthrough in neonatal infection/sepsis, and the authors apply a C-section delivered piglet model and one infection model to build their story. This is the weaker point of the paper, the fact that they base their conclusions on one model. It is a good model I think, but still a model, and just one. The model is also quite aggressive and, based on ethical concerns I presume, very short.

Reply: Thank you for your comment. We have developed this model of neonatal infection and sepsis in preterm piglets several years ago. This is the only model that allows research in infected prematurely born animals (in contrast, rodent neonatal infection models that are not prematurely born). We believe this mimics more closely conditions in infected preterm infants. In addition, this is also the only existing neonatal infection model that allows the use of parenteral nutrition (PN), which is the main intervention in this research study. We are well aware of the need of supporting data from other types of datasets or models; thus this is the reason we have sought to analyze immune and metabolic data from human cohort as well as in vitro studies. However, it is important to emphasize that what we ultimately propose here are proof-of-concepts for the use Galactose and Glucogenic amino acids (GAA) in PN for preterm newborns. The new PN paradigm described in this work should not be directly applied in preterm infants but require refinement and further development to fit the nutritional needs of infants. This is something that we are actively working on. For example, we are currently investigating the efficacy of replacing all glucose in PN with a combination of Galactose and GAAs.

We do agree that our model is relatively short. Yet, the main purpose of the experiments were to test the effects of PN composition on the acute infection responses and to rescue preterm animals from life-threatening sepsis. We considered extending the experiments to 24h, however, in the pilot phase of this project we detected the vast differences in survival between the control and Galactose/GAA groups. This prompted us to shorten the model to 15 h, as we consider it important to collect tissue samples at euthanasia when most animals have relatively similar survival time to avoid the bias of postnatal organ maturation. Furthermore, since differences in survival were obvious at 15 h, we considered it unethical to extend the study further this would increase animal suffering. Nonetheless, the study has been approved by the Danish Animal Experiments Inspectorate.

Recently, we have developed a longer and milder model by delaying the infection challenge (still with *S. epidermidis*) for a few more days. This has led to a milder sepsis phenotype with lower incidence of sepsis but still with systemic inflammation. Most of the interventions we tested in the acute short models still reduce bacterial clearance and inflammation in the milder model. On the other hand, the acute model provides important information for protection against infection mortality that the milder model cannot (only protection against systemic inflammation). Thus, we firmly believe in the efficacy of the interventions tested in this manuscript. Further, we believe that further testing in milder models falls outside the scope of

this current manuscript and our current line of experiments will be part of a different publication.

Referee #1 (Remarks for Author):

1. The paper can make use of a very interesting dataset of 700 newborn children, followed for 3 years in terms of health issues and plasma markers, including metabolites. The "TCA" metabolites, measured and reported here would be easier to interpret is they were also complemented with blood glucose, lactate and preferentially also pyruvate and other gluconeogenic molecules like glycerol and amino acids. If they are available, they should be provided.

Reply: All metabolites are derived from LC-MS/MS metabolomics of collected plasma samples from mothers and their children. We have chosen to focus on the TCA metabolites and only selected those that are identifiable from both samples at birth and at 18 months of age. These metabolites are intermediate molecules which fluctuate less than glucose, lactate and amino acids, which are more affected by diet and physical activity. However, following the reviewer's comments, we have investigated how glucogenic amino acids are associated with infection and immune endpoints in human infants. We computed a glucogenic amino acid score (GAA score) in the same way as we computed the TCA scores. We can now show that GAA scores positively associate with TCA scores at birth, 6 and 18 months of age. Further, that, all GAAs contribute positively to the GAA score and importantly that GAA score was associated with less inflammation at 6 months of age, and at 18 months of age was associated with lower infection burden from 18-36 months, in similar way as TCA score. **These new findings** may underscore the importance of GAA in TCA cycle activities and infection protection in both animals and humans. The data are now included in a new **Supplemental Figure S10** and described at the end of the Results section (lines 371-382).

As for glycolysis metabolites, pyruvate was also detected and showed some association with immune response at 18 months and associated with lower burden of acute otitis (New Supplemental Table S2). However, this molecule plays a role in both glycolysis, TCA cycle activities and fatty acid oxidation, and we believe data related to pyruvate does not fully reflect TCA cycle activities and GAA metabolism. We now have included data on Pyruvate in Supplementary Table S2 and supplementary Figure S1M and address the findings in the results section (Lines 132-135, revised manuscript with tracked changes). For glucose and lactate, these metabolites were detected, but are highly variable with levels quickly changing postprandially, while the blood sampling timepoint in the human cohort was not controlled for this. Therefore, we believe that inclusion of glucose and lactate would not provide relevant information in connection to TCA/glycolysis activities.

We hope including GAA score of all detected GAAs and pyruvate will satisfy the reviewer's concerns.

2. Fig1B,C,D should mention, in the figure head, that this is maternal, maternal and child TCA levels resp.

Reply: Thank you for reminding us. Yes, now these figure panels 1B-F have been revised, including either maternal or child TCA levels.

3. I do not see the added value of Fig 1F in this paper? Is this pattern of response different than whole blood responses of adults or so? Fig 1G is a bit misleading and the title of the figure should mention piglets: it falls under a figure with title that refers to children, while 1G deals with piglets. Though the data are clear and data significant, I would urge the authors to be a bit more reluctant with their statements that "improved survival was driven by higher fumarate and malate levels". I would suggest to talk about associations rather that cause & consequence.

Reply: The data in both Fig. 1E and F reflects how TCA score is related to blood immune cell composition (Fig. 1E) and ex vivo blood cell immune response to antigen challenges (Fig. 1F) from the same blood samples collected at 18 months of age. In our point of view, data in Fig. 1F showed that TCA scores are critical to mediate anti-inflammatory response via better control of IL-18 and IL-1beta release. Considering TCA score being associated with less infection burdens and less IL-18 and IL-1beta response, we can speculate that TCA scores reduce pro-inflammatory immune responses. It is important to pave the way for studies using animal model.

We agree with the reviewer that Fig. 1G should mention piglets in the heading. Now it has been revised. We also revised the texts on figure legend.

We also agree with the reluctance of the reviewer on the statement of "improved survival was driven by higher fumarate and malate levels". We have now revised this to association rather than cause and effects (lines 164-165).

4. Fig2 starts with asking an interesting and relevant question, namely how to 'boost' TCA activity in patients or animals in a safer way than using glucose. Fig 2A,B and C report data obtained by adding Gal or Glu to THP1 cells with S. epidermidis. The choice of the cells is not optimal and should be repeated with blood cells, like was done in Fig1F for example, because THP1, after all, is an immortalized cell line. Also, the 5 replicates are quite low for such an experiment, and it is unclear what the * refers to. What groups are compared with low conc Gal to come to this *? Why is there no condition where just S. epidermidis is added, maybe with and/or without classical carbohydrate sources used in tissue culture, glutamin? The choices of TNF and IL10 are also weird in relation to Fig1F, where IL1 and IL18 were concluded to be the bad cytokines and IL4 the good.

Reply: Thank you for this thoughtful comment. We agree that differentiated THP-1 cells do not fully recapitulate primary blood immune cells, and we acknowledge this as a limitation of the in vitro experiment. In this study, THP-1 cells were differentiated into macrophage-like cells before stimulation, and we used this system as a controlled screening model to test whether

galactose or glucose could modulate infection-induced inflammatory responses in a reproducible manner. The intention was not to directly mimic the complexity of the human or animal in vivo response, but rather to provide supportive mechanistic evidence alongside the primary in vivo findings.

A condition with S. epidermidis alone, without addition of galactose or glucose, was indeed included. Data were normalised to this infected condition without added sugar, which was set to a reference value of 1 within each independent replicate. We agree that this was not sufficiently clear in the original figure and have now clarified this in the revised figure legend (Lines 971-973 and 984-987). We have also clarified the meaning of the asterisks, which indicate significant differences compared with the infected condition without added galactose or glucose.

Regarding the choice of cytokines, TNF-α and IL-10 were selected as representative markers of pro- and anti-inflammatory responses in this macrophage-like cell system, based on their robust and reproducible induction in response to bacterial stimulation. We agree that these do not directly correspond to all cytokines highlighted in the in vivo and ex vivo datasets. However, the purpose of this assay was not to reproduce the full cytokine signature observed in animals or human blood cells, but to assess whether nutrient supplementation could directly modulate inflammatory activation in a simplified in vitro setting.

We further agree that the number of replicates was limited and that primary blood cells would provide higher physiological relevance. However, given that these experiments were intended as supportive screening data rather than a main experimental platform, we believe that the use of differentiated THP-1 cells was appropriate for this purpose. To avoid overinterpretation, we have further clarified the rationale, reference condition, and statistical annotations in the revised figure legend and manuscript text.

5. Fig2F: please use the full 0-100% Y axis, this will be more correct. Also, the colors of the lines are not clearly distinctive. Also, I want to ask if the pig model was ever run further than 15h? I think it is rather risky to draw conclusions based on the situation at one final time point. If this was ethically not an option, did the authors consider the use of maybe a mouse model or another model that could allow later timepoints?

Reply: We agree and have revised all survival curves to the full 0-100%. As for longer term survival, we can run our model for up to 24 h after infection. As mentioned previously, for this particular study, in pilot work we observed that most animals in the infected control group reached humane endpoints by 18-24 hours, whereas no animals in the intervention groups did. For this reason we decided to end the main experiment at 15 h. An important reason for this time of euthanasia is that it is critical to ensure fairly similar postnatal age of collected tissues at euthanasia for analysis (e.g., liver transcriptomics) to avoid a time bias between the groups. As a final reason as the reviewer stated, it is an ethical issue to extend the suffering during infection of animals, once we have already detected significant differences between groups. We consider European regulations on animal experimentation would apply the same way considering animal suffering, regardless of model types, in mice or piglets.

As far as we know, the only relevant mouse model of neonatal sepsis is run using cecal slurry model (see PMID: 30741260). This model is also acute, with most infected animals succumbing after 12h of infection challenge (for instance, see PMID: 38769383). Further, as we have mentioned in our initial responses to your general comments, it is unfeasible to infuse newborn rodents with PN. The alternative in a rodent model would be to inject galactose, glucose intraperitoneally before infection onset, which may yield mechanistic insight into immunometabolic infection responses, but offers very little translational clinical value.

As also responded to your general comments, we have established a longer and milder model of sepsis that can last for 5 days after birth with the challenge of infection on postnatal day 3, and follow-up of 48 h post-infection. This leads to mild sepsis phenotype with lower incidence, and is meant to test the effects of interventions on systemic inflammation. We are testing several interventions that were previously evaluated in the acute 15h model and we can still show the effects on inflammatory protection. However, within the scope of this manuscript for protection against infection mortality, the acute model is mostly appropriate. Future testing in a longer-term model will be considered in future publications.

6. The experiment of Fig2 should also report other readouts, that are perhaps more related to lethal outcome in sepsis. Were body temperature and lactate measured? If so, they have to be reported.

Reply: Lactate data has been measured and reported as part of supplemental Figure S2D. At euthanasia lactate levels were numerically higher in infected control vs. Galactose animals but did not reach significant difference.

These immature animals have poor thermoregulation in general and we have previously shown that their body temperature does not rise in response to the infection (PMID: 32536925). For this reason, in this work, we therefore did not measure temperature.

7. Figure 3 is, without doubt, the selling article of the paper. Though my concerns about THP1 also hold true here, and the Y axis of the survival curve, the data nicely show the protective impact of GNEO amino acids in this particular piglet model (which is still far from a newborn human study of course). The extra experiment in Figure 6 looks redundant to me. The biggest questions I have after reading the paper are the following: while starting the paper with human neonatal infection/sepsis, the paper quickly goes into a C-section delivered piglet model, never to really return to human neonatal sepsis. In other words, the readouts in Fig1 that suggest that TCA activity is important in surviving/reducing infections (not a big surprise as such) eventually leads to conclusions about the sugar source or even that gluconeogenesis should be stimulated to let the patient produce his/her glucose him/herself, by adding amino acids. The authors should now return to the neonates and their mothers and measure these amino acids and see if their concentration related to their infections. I expect the authors to address this.

Reply: We believe that not only the survival data are convincing, but all other paraclinical outcomes (blood gas, plasma cytokines and omics data) are critical to support the protective effects of GAA intervention. In our point of view, extra experiment in figure 6 is critical as it provide the *direct* effects of GAA on the background of restricted glucose provision. Data in Figures 3-5 compared a standard glucose-rich PN vs. Restricted glucose plus GAA. Since we have previously shown that reduced glucose provision also attenuates inflammation in this model, the observed effects could stem from either restricted glucose or GAA or both. The extra study in Figure 6 convincingly showed that direct supplementation of GAA led to increased blood glucose levels, presumably through gluconeogenesis, which potentially could avoid hypoglycemia in clinical situations. Further, GAA supplementation directly contributed to anti-inflammatory state, as shown by reduced blood TNF-alpha and IL-6 levels.

As also responding to your question 1, now following your comments, at the end of the Results section (lines 371-382, and supplemental Figure S10), we now returned to the human cohorts and identified the levels of these glucogenic amino acids, and showed convincingly that levels of GAAs at 18 months of age were associated with less infection burdens detected between 18-36 months of age. Further, GAAs levels at 6 months of age were also associated with lower levels of inflammatory markers CRP and IL-6, further strengthening the findings from animal model. We hope these extra data and analyses can satisfy some of your concerns.

8. Another question relates to the fact that this is a highly experimental model where animals are treated quite instantly with the metabolites. It is known that sepsis leads to fast block of gluconeogenesis in the liver. Therefore, it would be essential to test the AAs therapy when these are applied to the piglets quite some time after onset of sepsis, perhaps also in a model that is less aggressive and does not lead to deaths in 15h.

Reply: We agree with this chain of thought if GAA's (or galactose for that matter) were to be used as a therapeutic intervention initiated after an infection is suspected. However, we believe that once an infection is detected in the clinic (either through clinical signs or infection biomarkers or blood culture) the pro-inflammatory response may already be at a stage where they cannot be stopped by nutritional interventions. We have previously shown that reduction in glucose provision does not stop sepsis development in our model if it is done once symptoms occur (around 3 hours after infection in our model system). Likewise, we have shown that it appears that very early immune responses are key to surviving in this model system. See PMID: 39992703.

What we propose is a novel parenteral nutrition paradigm, where some or potentially all of the glucose in PN is replaced by galactose and GAAs. This novel PN could be used directly from birth for all very preterm infants, to prevent them developing early hyper-inflammatory responses to infections, and thereby hopefully prevent sepsis. All experiments thus deal with **sepsis prevention, not sepsis treatment** and we considered that further experiments testing the interventions after infection challenge or after sepsis onset as outside the scope of this work. We have edited the discussion to iterate this point (lines 449-451, 454-459, and 491-494).

Further, as mentioned earlier in previous answers, we have developed a less aggressive model. We are currently evaluating the effects of a combined galactose and GAA based parenteral nutrition, given from birth until infection challenge on postnatal day 3. These data will be published in a future manuscript, but we can share that a combined galactose and GAA PN prevents hyperglycemia and appears safe if given from birth to uninfected animals. And crucially, after infection onset it improves bacterial clearance and anti-inflammatory immune responses. However, much more pre-clinical work is needed to optimize the PN formulation and show safety before it can enter clinical trials. Regardless, the combined approach of galactose and GAA also fall outside the scope of the current manuscript.

9. Finally, the authors should consider to what extent their findings are selective for the E. epidermidis model, so another model would be very useful to validate their findings. Reply: Considering a majority of cases of late-onset sepsis (LOS) are caused by *S. epidermidis* infection (up to 75% of all LOS cases in some countries, Strunk et al, 2021, Clin Infect Dis), this is one of the most relevant bacteria to model. But we agree with the reviewer that the finding in this model may or may not be fully translational to cases infected with other pathogens. We address this limitation in the manuscript, lines 483-485.

Referee #2 (Comments on Novelty/Model System for Author):

The integration of cohort, in vitro, and animal data is a major strength.

Reply: Thank you for your comments. We also consider the combination of in vitro, animal data and human cohort is quite unique to support our findings.

Referee #2 (Remarks for Author):

This manuscript examines how the tricarboxylic acid (TCA) cycle activity shapes neonatal defense against infection. Using human birth-cohort metabolomics, in vitro macrophage experiments, and a preterm piglet sepsis model, the authors show that boosting hepatic TCA cycle flux via galactose substitution or glucogenic amino acid supplementation can enhance disease tolerance and resistance during neonatal sepsis. The study identifies metabolic rewiring as a potential therapeutic approach to improve infection outcomes in vulnerable newborns.

Reply: Thank you for your comments and summary.

Major Comments

1. The integration of cohort, in vitro, and animal data is a major strength. Some strengths: strong translational design, mechanistic integration across species, innovative nutritional

intervention concept, high-quality omics data. However, the manuscript would benefit from further discussion of TCA activity as a correlative biomarker and/or a causal driver of protection. For instance, discuss potential alternative explanations (e.g., gluconeogenesis vs. TCA flux vs. fatty acid oxidation contributions), and strengthen the mechanistic link between hepatic rewiring and immune modulation.

Reply: We fully agree with this comment, it is possible that the association between increased activity in the TCA cycle and inflammation/infection risk is mediated by other closely related metabolic pathways. As per comments from other reviewers, we have adjusted the causal language throughout the paper to reflect this. We also add further text to discuss other possible pathways contributing to the infection protection (lines 438-448). It is however clear from our data that the TCA cycle, the central metabolic pathway of mitochondria, does associate with decreased inflammation and infection burden, and importantly that TCA cycle activity can be boosted by nutritional modulation during infections.

2. On the human data, the associations between plasma TCA metabolites and the burden of infection are compelling. However, causality cannot be inferred, and it was recognized in the study's limitations.

Reply: Thank you for your constructive comments. We fully agree. We clearly stated in the results that those human data are associations, but not causation, and state this limitation in the end of the Discussion. These human data are important as it serves as background and confidence for validating the association as well as testing the effects of interventions in pig model.

3. For instance, could plasma TCA metabolites reflect general metabolic fitness rather than immune-specific mechanisms? Could the authors discuss the separation between maternal and infant metabolomes?

Reply: Yes, the TCA scores are derived from human plasma samples and as such reflect the metabolism of the whole host and not just the immune system. This is of course a limitation in exploring the relationship between immune cell metabolism and infection responses. However, the association between plasma TCA scores and infection risk/immune responses supports the notion that whole body metabolic state is associated with immune cell function. And whole body metabolism is easier to modulated by diet or pharmaceuticals than targeting metabolism of individual immune cell populations (lines 394-396). We also tried to mark in Figure 1 the clear separation of maternal and infant data. Following the reviewer's suggestions, we have now added texts in the Discussion about the distinction between maternal and infant metabolomes (lines 474-477).

4. The preterm pig model is highly valuable. Explicitly define how dosing compares to current neonatal PN practice. Is there a risk involved in the proposed protocol, such as hypoglycemia, nitrogen load, and acid-base balance?

Reply: We have stated in the Discussion and throughout the paper that this PN paradigm testing in pigs is a **proof-of-concept** and the precise PN composition may not be directly translational to human preterm infants. Regardless, we strongly believe that the concept of lowering PN glucose dose and supplemented GAA leading to more stable glucose homeostasis and less risk of both hypo- and hyper-glycemia and less sepsis risk is highly applicable in human preterm infants.

We have based our carbohydrate provision on ESPGHAN guidelines, but we included a higher proportion of amino acids to meet the pig's nutritional requirements due to the fact that the growth rate of newborn pigs is much higher than humans. As such, the overall nitrogen load in pigs would be considered higher than in preterm infants requiring PN. We have further added some explanation in the limitation of the study at the end of the Discussion. Thank you for this points (lines 490-494).

Since the completion of the experiments in this paper we have started developing a novel PN solution, combining galactose and glucogenic amino acids with the traditional PN components (Glucose, intralipid) in an effort to create a product in line with current ESPGHAN guidelines on macro- and micronutrients. We are actively testing and optimizing this in the preterm pig model with the goal to suggest new PN paradigms for infection-sensitive preterm infants. This however will be the subject of future publications and we feel it is outside the scope of this paper.

5. Provide a schematic summarizing the effects of resistance vs. tolerance across interventions. Is there a risk of immunosuppression with excessive tolerance?

Reply: Following the reviewer's comment, we have now revised the Figure 5 to include schematic summary of the effects of the intervention on resistance and tolerance (Fig. 5B). We can emphasize that the unique results of the GAA intervention study were that there appeared to have no risk of immunosuppression as the effect of GAA was not only to enhance tolerance. The intervention enhanced resistance at early phase of infection (3h) by clearing bacteria more efficiently, causing less infection burden for the host, followed by enhanced tolerance at later phase of infection to protect against organ injury. It is also important to emphasize that from Figure 3, the overall bacterial density over time of infection course was lower in the intervention group. All these events pointed to the overall effects to balance the levels of resistance and tolerance by GAA intervention, rather than skewed tolerance.

6. Is there evidence for the lines 141-145? It seems to be overstating.

Reply: Yes, these are based on data in Fig 1. E and Supplemental Fig. S1G-H. Citrate was associated with enhanced IL-10 response to ex vivo blood stimulation, and alpha ketoglutarate was associated with lower levels of inflammatory cells (analyzed by flow cytometry), as well as less IL-1beta and CXCL8 response to blood stimulation.

7. TCA cycle intermediates were measured in liver tissue. Are there tissue alterations? Did the authors perform histology analysis on the tissue?

Reply: We did measure plasma levels of liver enzymes as markers of liver injury. Especially ASAT and ALAT showed significant differences by interventions, indicating the effects on liver injury. We unfortunately did not collect fixed tissues for histology.

8. Lines 167-169: How can we explain the lower levels of TCA activity? One possibility is mitochondrial dysfunction. Have you looked at it? This reduced activity appears to be a consequence of survival rather than its cause.

Reply: Decreased levels of TCA activity could be indeed due to increased mitochondrial dysfunction or rewired metabolism to increased glycolysis. We have previously shown from hepatic transcriptomics comparing infected survivors and non-survivors that non-survivors had down-regulated pathways related to oxidative phosphorylation and increased glycolysis (https://pubmed.ncbi.nlm.nih.gov/39213794/) but did not detect pathways related to mitochondrial dysfunctions. Of course, the lower TCA activities can be either the consequence or the cause of infection mortality like the reviewer suggested, but we suspected that it may contribute to the cause of mortality, and that prompted us to test intervention studies that can boost TCA activities in infected piglets to see if this can prevent infection mortality.

9. Figure 2C: There is an asterisk in the graph. Revise it.

Reply: The asterisks indicate significant changes compared with controls, we have revised to figure legends to be clearer.

10. Figure 2E: Did the animals actually die, or is it a prediction?

Reply: No, all animals were euthanized after reaching predefined humane endpoints. These are the same criteria that we use to define sepsis (pH <= 7.1 plus clinical symptoms of hypoperfusion and deep lethargy), meaning a case of sepsis was counted as a case of mortality (see lines 634-637).

11. Figure S2G-I: Check the statistics in Figure S2M. This pattern holds for the 15-hour time point for hemoglobin and monocytes, but not for neutrophils. The CON-sGal group has higher levels of TNF, Hb, creatinine, and creatine kinase (see statistics). Further discuss it. Line 217-218: How do you explain that? Are the liver injury markers not affected by galactose supplementation?

Reply: Thank you for your comment. We have carefully rechecked the statistics presented in Figure S2M. To assess group differences at specific time points (3, 6, 12, and 15 h), continuous data were analyzed using a linear mixed-effects model, with group, sex, and birth weight included as fixed effects and litter included as a random effect. Model assumptions were

evaluated by examining the normality and homogeneity of residuals, as well as the relationship between residuals and fitted values. Variables that did not meet the normality assumption were log-transformed before analysis. For creatine kinase, values in the CON-sGAL group were generally lower than those in the CON-sGLU group, although one animal in the CON-sGAL group showed a markedly high value. Accordingly, this comparison showed only a numerical trend, but did not reach statistical significance ($P = 0.09$).

Yes, the pattern did not hold for neutrophils at 15 h, partly because there were two animals with high neutrophil counts in the control groups. The galactose group still have numerically higher mean of neutrophil counts than the infected controls. For TNF-alpha, the difference between 2 uninfected control groups was very minor (even though it was significant) and we believe that did not have clinical relevance, compared to the levels in infected animals (Y axis is presented in log scale). Now we included some discussion as the reviewer suggested when describing these results of TNF, Hb, and biochemical data (lines 217-218).

Yes, liver injury markers were not affected by galactose supplementation. We have explained further in lines 234-236. This was likely due to the fact that galactose only slowed down the glycolysis but did not rewire metabolism completely to oxidative phosphorylation. And also considering the minor effects on cytokine levels (compared to the GAA intervention), it is expected that minor effects of galactose on systemic inflammation can lead to unobvious changes in organ injuries. That actually motivated us to explore GAA intervention as a more effective way of rewiring metabolism that affected the infection outcomes.

12. Figure S4C: groups SE-sGLU had high dispersion; please check the statistics. Note that con-rGLU-GAAs also increased lactate in 12 and 14 hours (Fig 3G). Figure S4G, AST was changed.

Reply: Thank you for this important comment. We agree that the SE-sGLU group showed relatively high dispersion. We therefore re-examined the analysis and performed a robust mixed-effects model as a sensitivity analysis, with Group, Gender, and Birth weight included as fixed effects and Litter as a random effect. The estimated group difference remained similar in direction and magnitude (estimate ≈ -13), supporting the robustness of the original finding. These results indicate that the reported effect was not driven by a small number of influential observations.

Yes, it appeared that the uninfected GAA animals had transiently higher lactate levels than uninfected controls, which were then normalized at 15 h (but still slightly higher than those in uninfected controls). And yes, AST in figure S4G showed tendency to difference between the groups.

13. Figure S4J and K, con-rGLU-GAAs had increased albumin and cholesterol levels. It was not discussed.

Reply: Correct that the intervention leads to increased albumin and cholesterol levels in uninfected controls, very likely due to rewired metabolism due to the intervention. This is likely reflected by changes in metabolic pathways as shown in transcriptomics analyses (Fig. 4B, S5B). Now we included some discussion when mentioning these data in the Results (lines 269-270).

14. Figure S4L-O, different statistics were done, Pin(SE) lymphocytes; there was no statistical difference, but between SE-groups on day 15, there is a difference. However, between-group Pint(SE) did not differ. These are the two key cells; monocytes were much lower, considering removing them.

Reply: We have analyzed the difference between groups over time of the infection course (Ptreat(SE)), the changes over time (Ptime(SE)), and interaction of time and treatment (Pint(SE)). And yes, we often observed immune cell depletion over the first 12h of the infection course, and the intervention leading to better outcomes often showed lower degree of immune cell depletion at early phase of infection. For lymphocytes, it is true that the interaction between time and group was not significant but showed tendency (P= 0.08). We also agree that lymphocytes and neutrophils are key cell types which often showed differences by interventions. And monocyte levels were much lower, but these are obtained data and we prefer to show them for transparency.

15. The figures S6, D, and H were cut.

Reply: we are unsure what the reviewer mean when stating the figure S6D and H were cut. The heatmap included all up and down-regulated genes, but we separated up- and down-genes by some white space for better illustration. Nothing has been cut.

16. Line 341, "lower pCO2," was not different.

Reply: pCO2 levels in the GAA group showed tendency for lower levels over time, and also significantly lower levels than control groups at 12 h.

17. Line 343, "GAAs enhanced bacterial clearance at both 3 and 6 h, as well", but not at 15 hours. "as better blood leukocyte replenishment and reduced inflammation at the later phase of the experiment", decreased TNF, but not IL6 (just 12 hours) or altered IL-10.

Reply: Thank you, we have added extra texts as you suggested (now in lines 346-348).

18. Check for typos, as on line 403.

Reply: We have revised, thank you

19. Why choose perform metabolomics on plasma over blood immune cells?

Reply: Thank you for this important comment. The choice to perform metabolomics in plasma and liver tissue, rather than in isolated blood immune cells, was intentional and aligned with the main aim of the study. Our objective was to investigate how infection alters systemic and hepatic metabolism in relation to host defence, rather than to specifically dissect immune cell-intrinsic metabolic programmes. In this context, plasma provides an integrated readout of circulating metabolites reflecting whole-body metabolic status, whereas the liver represents a central organ for metabolic coordination during infection. Together, these compartments were considered most appropriate for addressing the biological question of the present study.

We fully agree that metabolomic profiling of isolated blood immune cells could yield additional mechanistic insight into immunometabolic regulation at the cellular level. However, such analyses would require a different experimental setup and were beyond the scope of the current work. We have now clarified this rationale in the revised manuscript.

Minor Comments

20. Standardize terminology (OxPhos vs oxidative phosphorylation).

Reply: We have revised these accordingly.

21. Define all acronyms at first mention.

Reply: We have revised these accordingly.

22. Check consistency in statistical reporting

Reply: We have carefully checked the consistent reporting of statistics.

Referee #3 (Comments on Novelty/Model System for Author):

This manuscript is possibly paradigm shifting with high translational implications. The authors provide a detailed description of their models which span human to cells. The piglet model is very short lived and doesn't provide information about the long term impact.

Reply: Thank you for your constructive feedback. We also believe that our results can lead to shift in paradigm of using parenteral nutrition for infection-sensitive preterm infants.

We have chosen the acute infection model of neonatal sepsis to evaluate how immunometabolic intervention can rescue infected animals from life-threatening infections. The acute mortality in neonatal sepsis occuring within 12-15h of infection is similar to comparable neonatal sepsis model in newborn mice induced by cecal slurry (Firanza et al, 2025, Sci Rep). We agree that acute model does not provide information about long-term impacts (e.g., on neurodevelopmental outcomes or chronic organ injury), but our story here focuses on acute

infection survival. Thus, long-term impacts of infection and intervention fall outside of the scope of this study.

Referee #3 (Remarks for Author):

1. This important work has potentially paradigm shifting translational applications for a considerable problem in the NICU. The work is comprehensive stemming from cellular data to in vivo and human data.

Reply: Thank you for your comments.

Some significant concerns arise nonetheless:

2. The experiments are very short lived not allowing for a better understanding of the duration of the effect and any longer term implications. Further follow up would be helpful if some animals were allowed to survive and be euthanized at later timepoints.

Reply: Please also see our response to reviewer 1. We agree that our model is relatively short. However, the main purpose of the experiments was testing how the composition of PN affected the acute infection responses and to evaluate if novel PN compositions could rescue preterm animals from life-threatening sepsis. The model itself captures the clinical phenotype and paraclinical signs of neonatal sepsis.

As explained to Reviewer 1, we have previously extended the study to 24 h, and in a pilot study, we observed that all infected controls died within 18 hours post-infection, while more than 90% of intervened groups survived. This prompted us to shorten the model to 15 h, as we consider it important to collect tissue samples at euthanasia when most animals have relatively similar survival time to avoid the bias of postnatal organ maturation. On top of this, we have detected significant difference after 15 h in a quite severe infection setup, and this would be considered unethical to extend the study further that would increase the animal suffering.

Further, we have recently developed a longer and milder model by delaying the infection challenge (still with *S. epidermidis*) for a few more days. This has led to a milder sepsis phenotype with lower incidence of sepsis but still with systemic inflammation. Most of the interventions we tested in the acute short models still reduce bacterial clearance and inflammation in the milder model. This is a work in progress for future publications. So, we firmly believe in the efficacy of the interventions tested in this manuscript. On the other hand, the acute model provides important information for protection against infection mortality that the milder model cannot. Therefore we believe that further testing in milder models with longer term follow-up is relevant, but fall outside the scope of this current manuscript.

3. No weights over time are provided for the animal data, likely to the short term nature of the experiments and no survival studies. However this needs to be clearly stated in the limitations section.

Reply: Thank you for this comment. We agree that the absence of longitudinal body weight data should be acknowledged as a limitation and have now clarified this in the revised manuscript (lines 490-491). In the present study, only birth weight was recorded and used for stratified randomisation. Repeated body weight measurements were not performed during the 15 h experimental period. This decision was based on both practical and biological considerations. During the first day of life, the animals experienced both natural physiological bodyweight loss of 5-10%; and here in this study infection certainly also affected body weight. As a result, short-term body weight changes would be difficult to interpret as a robust indicator of disease progression or treatment response. In addition, in our previous work using this model, we did not observe clear differences in body weight between infected and non-infected animals over comparable experimental periods. We therefore prioritized other clinical and biochemical readouts in the current study. This limitation has now been stated explicitly in the revised manuscript.

4. The authors didn't measure glycolysis in this model which would be important and easily doable from the existing dataset. Does glycolysis reduce significantly? Glycolysis is important to cell proliferation in many tissues.

Reply: We are unsure which glycolysis measures the reviewer referred to with the existing dataset from the animal model. In blood we reported Lactate levels during the study and based on hepatic transcriptomic analysis in Fig. 4-B and Fig. S5D-F, especially in Fig. 4B, it is very clear that most of the genes involved only in glycolysis were significantly down-regulated in GAA group, compared to infected controls, but not between the 2 groups in non-infected conditions. This is important observation, suggesting that the intervention itself did not suppress glycolysis (which is important for cell proliferation as the reviewer pointed out), but only prevented the acceleration of glycolysis under infected conditions, thereby preventing hyper-inflammatory response. We further clarified this point when discussing results in the Fig. 4A-B in lines 307-309. Thank you.

5. There needs to be transparency around the drop in glucose seen initially. What was this?Is this clinically relevant? I disagree with the authors that humans, especially premature ones, have a full capacity for gluconeogesis. This is the reason we worry about hypoglycemia clinically especially in babies that are growth restricted in utero. Did the cohort distinguish between babies from the groups evaluated?

Reply: Both numerically and statistically, glucose levels did not drop in each group from 3 to 6h. There was only slight tendency for lower glucose levels in infected galactose group vs. uninfected galactose group at 3h (Fig. 2A), but this was not statistically significant. Any minor drop of glucose levels in infected vs. uninfected animals during the first few hours can be explained by likely use of blood glucose by immune cells to run glycolysis and provide ATP for inflammatory response. We do not know the real situation in human preterm infants, as glucose levels are rarely continuously monitored in clinical practice, and it is impossible to identify the exact timepoint when infection starts until later when clinical symptoms appear.

We do not state that the premature newborns have full capacity for gluconeogenesis. We referred to previous publications that report results in preterm infants that their liver can run gluconeogenesis, pointing to the options that we can explore interventions using gluconeogenesis pathway to avoid hypoglycemia. To our knowledge there is no data that shows that the gluconeogenesis pathway is further inhibited during neonatal infections. On the contrary, post-mortem quantitation of hepatic glucose-6-phosphatase activity has been reported to be *higher* in preterm infants that died due to early life infection, compared to those that died from respiratory failure (PMID: 10920788). Likewise, in our animal model we have seen upregulation of gluconeogenesis related genes in glucose restricted infected animals (PMID: 39992703). Similar to preterm infants, preterm pigs likely have reduced capacity for gluconeogenesis, compared to term pigs. Still, Fig. 6 shows that GAA supplementation increases blood glucose levels by under the same glucose provisions. Once this study is published, we believe it will trigger good discussion among clinicians and lead to trials testing glucogenic amino acids, to potentially stabilize blood glucose and avoid hypoglycemia. We have added texts to clarify of the reduced capacity of gluconeogenesis in preterm infants, compared with term infants (lines 429-430).

The human cohort did not record frequent blood glucose levels shortly after birth.

6. Is there a clear rationale for the selection of time points for blood sampling in the children? This is important to better understand the intent.

Reply: Thank you for your comments. The human cohort used is a deeply phenotyped Danish birth cohort. It was designed and established before 2010 with selected time points of blood sampling to match suggested timeline for routine health check in the children health program in Denmark. We have further clarified this important detail in the Methods section, lines 517-519.

7. Overall, important insights are provided in this well executed study.

Reply: Thank you. We know that compared to rodent model, pig model is less capable to provide full mechanistic insights, but on the other hand possesses higher translational values due to closer mimicking of body size, immune system and possibilities of using parenteral nutrition and repeated blood sampling.

6th May 2026

Dear Dr. Nguyen,

Thank you for submitting your revised study. We have now received the feedback from the referees, and as you will see below, they are satisfied with the revisions. I will therefore be able to accept your manuscript once the following minor editorial matters are addressed:

1/ Manuscript text:
- Accept the previous changes, and only keep in track changes mode any new modification.
- Please confirm that senior authorship in this case means corresponding authorship.
- Please correct the headings and order of the sections in the manuscript text to: Abstract / The Paper Explained / Introduction / Results / Discussion / Methods / Data Availability / Acknowledgements / Disclosure and Competing Interests Statement / References / Figure Legends / Expanded View Figure Legends.
- Methods:
o Remove the Reagent table from the manuscript text and upload it as a separate file. Please also list the cells used in the study.
o Clearly distinguish between experiments that have been performed and published previously, and experiments/methods new to your study.
o References to publish work: please check references for COPSAC cohort (ref #13 and 14) and for animal surgery (ref #19)
o Please indicate if the cells were tested for mycoplasma contamination and authenticated.
o Animals: please provide the housing and husbandry conditions, include a statement of compliance with ethical regulations.
- BioRender: remove from the figure legends and add a dedicated section to the Methods using this format:
Graphics:
(some of the... OR Figure #... OR synopsis) Graphics were created with BioRender.com
- Data availability section: please provide specifics URLs for the deposited data. Please remove "Any additional information required to reanalyze the data reported in this paper is available from the lead contact upon request."
- Acknowledgments: The list of funders provided in our system will be linked in published articles to PubMed; it is therefore important that the list in our system is complete and accurate. Should NNF20SA0064, Grant No. 9462283 and NNF23SA0084103 also be entered in the list of funders?
- Please remove Author contributions from the manuscript text, and provide instead CRediT (Contributor Role Taxonomy) terms in the submission system. These replace a narrative author contribution section in the manuscript.
- "Declaration of interests" should be renamed to "Disclosure and Competing Interests Statements".
- References: as noted above, inconsistencies were found in some references that did not seem to match the text/method in the manuscript, please check carefully. Reformat the references to alphabetical order, and remove DOIs for published articles.

2/ Figures:
- Please provide individual data points where possible.
- EV figures should be uploaded individually as high resolution figures in portrait orientation. Please remove the figure legends and move them to the manuscript text file, after the main figure legends and under the heading "Expanded View Figure Legends".
- You currently have two EV figures and 8 Appendix figures. We encourage you to make more Appendix figures into EV figures, as these are directly visible within the main manuscript.
- There are figure callouts for a Figure S2J and S4B in the manuscript text, please correct.
- Please divide the dataset files into 5 datasets, label them "Dataset EV1" etc. and add a legend to each dataset file, in a separate tab/worksheet.
- Appendix: add a table of contents with page numbers.
- Please address the queries from our data editors in the figure legends:
1. Please note that the exact p values are not provided in the legends of figures 1B, C, D, G; 2A-C; F-H, J-L; 3A, B; E-N; 6C, D, E, F, G, H, I, J, K, L; EV1 N; EV1 O; EV2 G-I, J-L
2. Please indicate the statistical test used for data analysis in the legends of figures 1B-G; 2A-C, F-L; 3A, B, E-N; 4A, C, D; 6C, D, E, F, G, H, I, J, K, L; 7B, EV1 G, O; EV2 K-M.
3. Please note that the box plots need to be defined in terms of minima, maxima, centre, bounds of box and whiskers, and percentile in the legends of figures 2F-H, J-L; 3E-N; 6C, D, E, F, G, H, I, J, K, L
4. Please note that information related to n is missing in the legends of figures 2A-C, F, G, H, J, K, L; 3A, B, E-N; EV1 G, O; EV2 J-L
5. Please note that the error bars are not defined in the legends of figures 1D, G; 2A-C; 3A, B; EV1 O
6. Please note that the measure of center for the error bars needs to be defined in the legends of figures 1B, EV1 G, EV2 J-L

3/ Please provide a complete author checklist, which you can download from our author guidelines. Please insert information in the checklist that is also reflected in the manuscript. The completed author checklist will also be part of the RPF. The file currently labeled Author checklist is the Source Data checklist.

4/ Source Data:
Please remove the file Figure 1A, which is empty.
Please check the raw data provided for Figure 1F (data for R848 and HDMAPP is identical).

5/ The paper explained:
Please carefully check the text for spelling and grammar and move the TPE to the main manuscript text file.

6/ Synopsis:
I introduced minor edits in your text to fit our style and format, please let me know if you agree or amend as you see fit:

The role of nutrition in regulating infection defense in preterm newborns remains poorly understood. The glycolysis-TCA cycle axis has been identified as a key modulator of infection outcomes, suggesting potential nutritional interventions to improve protection against neonatal sepsis.
- In a cohort of 700 infants, TCA cycle metabolites in early life were associated with decreased infection burden and systemic inflammation.
- The same metabolites were validated in infected preterm pigs, showing association of higher metabolite levels with infection survival.
- Using galactose instead of glucose in parenteral nutrition for infected preterm newborn piglets improved survival, enhanced mitochondrial metabolism, and promoted disease tolerance during infection.
- In the infant metabolome, TCA cycle intermediates correlate strongly with glucogenic amino acids, which in turn are associated with reduced infection burden and lower levels of inflammation.

Please upload the visual abstract as a separate png/jpeg/tiff file, 550 px wide and 300 px wide. Please make sure that the text remains legible.

7/ As part of the EMBO Publications transparent editorial process initiative (see our Editorial at http://embomolmed.embopress.org/content/2/9/329), EMBO Molecular Medicine will publish online a Review Process File (RPF) to accompany accepted manuscripts.
This file will be published in conjunction with your paper and will include the anonymous referee reports, your point-by-point response and all pertinent correspondence relating to the manuscript. Let us know whether you agree with the publication of the RPF.
Please note that the Authors checklist will be published at the end of the RPF.

I look forward to receiving your revised manuscript.

Yours sincerely,

Lise Roth

Lise Roth, PhD
Senior Editor
EMBO Molecular Medicine

***** Reviewer's comments *****

Referee #1 (Remarks for Author):

This revised paper has significantly improved and the authors have adequately replied to all questions of the reviewers, to my satisfaction.

Referee #2 (Remarks for Author):

The authors fully answered my comments.

Referee #3 (Comments on Novelty/Model System for Author):

The author addressed all queries.

The authors have addressed all minor editorial requests.

26th May 2026

Dear Dr. Nguyen,

Thank you for submitting your revised files. We are pleased to inform you that your manuscript is accepted for publication and is now being sent to our publisher to be included in the next available issue of EMBO Molecular Medicine.

Your manuscript will be processed for publication by EMBO Press. It will be copy edited and you will receive page proofs prior to publication. Please note that you will be contacted by Springer Nature Author Services to complete licensing and payment information.

You may qualify for financial assistance for your publication charges - either via a Springer Nature fully open access agreement or an EMBO initiative. Check your eligibility: https://link.springer.com/journal/44321/how-to-publish-with-us

Should you be planning a Press Release on your article, please notify the production team via this form in order to coordinate publication and release dates: https://forms.cloud.microsoft/e/eH4A8JVP6g

If you have any questions, please do not hesitate to contact the Editorial Office. Thank you for your contribution to EMBO Molecular Medicine.

Yours sincerely,

Lise Roth

Lise Roth, Ph.D
Senior Editor
EMBO Molecular Medicine

>>> Please note that it is EMBO Molecular Medicine policy for the transcript of the editorial process (containing referee reports and your response letter) to be published as an online supplement to each paper. If you do NOT want this, you will need to inform the Editorial Office via email immediately. More information is available here: https://link.springer.com/partners/embo-press/editorial-policies#Peer%20review

EMBO Press Author Checklist

Corresponding Author Name: Duc Ninh Nguyen	
Journal Submitted to: EMBO Molecular Medicine	
Manuscript Number: EMM-2026-23290	

USEFUL LINKS FOR COMPLETING THIS FORM
The EMBO Journal - Author Guidelines
EMBO Reports - Author Guidelines
Molecular Systems Biology - Author Guidelines
EMBO Molecular Medicine - Author Guidelines

Reporting Checklist for Life Science Articles (updated January
This checklist is adapted from Materials Design Analysis Reporting (MDAR) Checklist for Authors. MDAR establishes a minimum set of requirements in transparent reporting in the life sciences (see Statement of Task: 10.31222/osf.io/9sm4x). Please follow the journal's guidelines in preparing your
Please note that a copy of this checklist will be published alongside your article.

Abridged guidelines for figures
1. Data
The data shown in figures should satisfy the following conditions:
- the data were obtained and processed according to the field's best practice and are presented to reflect the results of the experiments in an accurate and unbiased manner.
- ideally, figure panels should include only measurements that are directly comparable to each other and obtained with the same assay.
- plots include clearly labeled error bars for independent experiments and sample sizes. Unless justified, error bars should not be shown for technical
- if n<5, the individual data points from each experiment should be plotted. Any statistical test employed should be justified.
- Source Data should be included to report the data underlying figures according to the guidelines set out in the authorship guidelines on Data

2. Captions
Each figure caption should contain the following information, for each panel where they are relevant:
- a specification of the experimental system investigated (eg cell line, species name).
- the assay(s) and method(s) used to carry out the reported observations and measurements.
- an explicit mention of the biological and chemical entity(ies) that are being measured.
- an explicit mention of the biological and chemical entity(ies) that are altered/varied/perturbed in a controlled manner.
- the exact sample size (n) for each experimental group/condition, given as a number, not a range;
- a description of the sample collection allowing the reader to understand whether the samples represent technical or biological replicates (including how many animals, litters, cultures, etc.).
- a statement of how many times the experiment shown was independently replicated in the laboratory.
- definitions of statistical methods and measures:
  - common tests, such as t-test (please specify whether paired vs. unpaired), simple χ2 tests, Wilcoxon and Mann-Whitney tests, can be unambiguously identified by name only, but more complex techniques should be described in the methods section;
  - are tests one-sided or two-sided?
  - are there adjustments for multiple comparisons?
  - exact statistical test results, e.g., P values = x but not P values < x;
  - definition of 'center values' as median or average;
  - definition of error bars as s.d. or s.e.m.

Please complete ALL of the questions below.
Select "Not Applicable" only when the requested information is not relevant for your study.

Materials

Newly Created Materials	Information included in the manuscript?	In which section is the information available? (Reagents and Tools Table, Materials and Methods, Figures, Data Availability Section)
New materials and reagents need to be available; do any restrictions apply?	Yes	Reagents and Tool Table

Antibodies	Information included in the manuscript?	In which section is the information available? (Reagents and Tools Table, Materials and Methods, Figures, Data Availability Section)
For **antibodies** provide the following information:<br>- Commercial antibodies: RRID (if possible) or supplier name, catalogue number and or/clone number<br>- Non-commercial: RRID or citation	Not Applicable	

DNA and RNA sequences	Information included in the manuscript?	In which section is the information available? (Reagents and Tools Table, Materials and Methods, Figures, Data Availability Section)
**Short novel DNA or RNA including primers, probes**: provide the sequences.	Yes	Dataset

Cell materials	Information included in the manuscript?	In which section is the information available? (Reagents and Tools Table, Materials and Methods, Figures, Data Availability Section)
**Cell lines:** Provide species information, strain. Provide accession number in repository **OR** supplier name, catalog number, clone number, and/**OR** RRID.	Yes	Reagents and Tools, Methods
**Primary cultures:** Provide species, strain, sex of origin, genetic modification status.	Not Applicable	
Report if the cell lines were recently **authenticated** (e.g., by STR profiling) and tested for mycoplasma contamination.	Not Applicable	Methods, and Reagents and Tools. The cell line was purchased directly from ATCC, and info from the provider mentioned they did perform STR profiling and confirm mycoplasma not detected.

Experimental animals	Information included in the manuscript?	In which section is the information available? (Reagents and Tools Table, Materials and Methods, Figures, Data Availability Section)
**Laboratory animals or Model organisms:** Provide species, strain, sex, age, genetic modification status. Provide accession number in repository **OR** supplier name, catalog number, clone number, **OR** RRID.	Yes	Methods
**Animal observed in or captured from the field:** Provide species, sex, and age where possible.	Not Applicable	
Please detail **housing and husbandry conditions**.	Yes	Methods

Plants and microbes	Information included in the manuscript?	In which section is the information available? (Reagents and Tools Table, Materials and Methods, Figures, Data Availability Section)
**Plants:** provide species and strain, ecotype and cultivar where relevant, unique accession number if available, and source (including location for collected wild specimens).	Not Applicable	
**Microbes:** provide species and strain, unique accession number if available, and source.	Yes	Methods

Human research participants	Information included in the manuscript?	In which section is the information available? (Reagents and Tools Table, Materials and Methods, Figures, Data Availability Section)
If collected and within the bounds of privacy constraints report on age, sex and gender or ethnicity for all study participants.	Yes	Methods

Core facilities	Information included in the manuscript?	In which section is the information available? (Reagents and Tools Table, Materials and Methods, Figures, Data Availability Section)
If your work benefited from core facilities, was their service mentioned in the acknowledgments section?	Yes	Acknowledgement

Design

Study protocol	Information included in the manuscript?	In which section is the information available? (Reagents and Tools Table, Materials and Methods, Figures, Data Availability Section)
If study protocol has been **pre-registered, provide DOI in the manuscript**. For clinical trials, provide the trial registration number **OR** cite DOI.	Yes	Methods
Report the **clinical trial registration number** (at ClinicalTrials.gov or equivalent), where applicable.	Yes	Methods

Laboratory protocol	Information included in the manuscript?	In which section is the information available? (Reagents and Tools Table, Materials and Methods, Figures, Data Availability Section)
Provide DOI OR other citation details if **external detailed step-by-step protocols** are available.	Not Applicable	

Experimental study design and statistics	Information included in the manuscript?	In which section is the information available? (Reagents and Tools Table, Materials and Methods, Figures, Data Availability Section)
Include a statement about **sample size** estimate even if no statistical methods were used.	Yes	Figure legends
Were any steps taken to minimize the effects of subjective bias when allocating animals/samples to treatment (e.g. **randomization procedure**)? If yes, have they been described?	Yes	Methods
Include a statement about **blinding** even if no blinding was done.	Yes	Methods
Describe **inclusion/exclusion criteria** if samples or animals were excluded from the analysis. Were the criteria pre-established?<br><br>If sample or data points were omitted from analysis, report if this was due to attrition or intentional exclusion and provide justification.	Yes	Methods
For every figure, are **statistical tests** justified as appropriate? Do the data meet the assumptions of the tests (e.g., normal distribution)? Describe any methods used to assess it. Is there an estimate of variation within each group of data? Is the variance similar between the groups that are being statistically compared?	Yes	Figure legends

Sample definition and in-laboratory replication	Information included in the manuscript?	In which section is the information available? (Reagents and Tools Table, Materials and Methods, Figures, Data Availability Section)
In the figure legends: state number of times the experiment was **replicated** in laboratory.	Yes	Figure legends and invidividual datapoints in the figures
In the figure legends: define whether data describe **technical or biological replicates**.	Yes	Figure legends

Ethics

Ethics	Information included in the manuscript?	In which section is the information available? (Reagents and Tools Table, Materials and Methods, Figures, Data Availability Section)
Studies involving **human participants**: State details of **authority granting ethics approval** (IRB or equivalent committee(s), provide reference number for approval.	Yes	Methods
Studies involving **human participants**: Include a statement confirming that **informed consent** was obtained from all subjects and that the experiments conformed to the principles set out in the WMA Declaration of Helsinki and the Department of Health and Human Services Belmont Report.	Yes	Methods
Studies involving **human participants:** For publication of **patient photos**, include a statement confirming that consent to publish was obtained.	Not Applicable	
Studies involving experimental **animals**: State details of **authority granting ethics approval** (IRB or equivalent committee(s), provide reference number for approval. Include a statement of compliance with ethical regulations.	Yes	Methods
Studies involving **specimen and field samples:** State if relevant **permits** obtained, provide details of authority approving study; if none were required, explain why.	Not Applicable	

Dual Use Research of Concern (DURC)	Information included in the manuscript?	In which section is the information available? (Reagents and Tools Table, Materials and Methods, Figures, Data Availability Section)
Could your study fall under dual use research restrictions? Please check biosecurity documents and list of **select agents and toxins** (CDC): https://www.selectagents.gov/sat/list.htm	Not Applicable	
If you used a select agent, is the security level of the lab appropriate and reported in the manuscript?	Not Applicable	
If a study is subject to dual use research of concern regulations, is the name of the **authority granting approval and reference number** for the regulatory approval provided in the manuscript?	Not Applicable	

Reporting

The MDAR framework recommends adoption of discipline-specific guidelines, established and endorsed through community initiatives. Journals have their own policy about requiring specific guidelines and recommendations to complement MDAR.

Adherence to community standards	Information included in the manuscript?	In which section is the information available? (Reagents and Tools Table, Materials and Methods, Figures, Data Availability Section)
State if relevant guidelines or checklists (e.g., **ICMJE, MIBBI, ARRIVE, PRISMA**) have been followed or provided.	Yes	Methods
For **tumor marker prognostic studies,** we recommend that you follow the **REMARK** reporting guidelines (see link list at top right). See author guidelines, under 'Reporting Guidelines'. Please confirm you have followed these guidelines.	Not Applicable	
For **phase II and III randomized controlled trials**, please refer to the **CONSORT** flow diagram (see link list at top right) and submit the CONSORT checklist (see link list at top right) with your submission. See author guidelines, under 'Reporting Guidelines'. Please confirm you have submitted this list.	Not Applicable	

Data Availability

Data availability	Information included in the manuscript?	In which section is the information available? (Reagents and Tools Table, Materials and Methods, Figures, Data Availability Section)
Have **primary datasets** been deposited according to the journal's guidelines (see 'Data Deposition' section) and the respective accession numbers provided in the Data Availability Section?	Yes	Data availablity
Were **human clinical and genomic datasets** deposited in a public access-controlled repository in accordance to ethical obligations to the patients and to the applicable consent agreement?	Not Applicable	
Are **computational models** that are central and integral to a study available without restrictions in a machine-readable form? Were the relevant accession numbers or links provided?	Not Applicable	
If publicly available data were reused, provide the respective **data citations in the reference list**.	Not Applicable	
